# A Novel Pseudogene Methylation Signature to Predict Temozolomide Outcome in Non-G-CIMP Glioblastomas

**DOI:** 10.1155/2022/6345160

**Published:** 2022-06-06

**Authors:** Bowen Li, Jiu Wang, Fangfang Liu, Rui Li, Weihong Hu, Amandine Etcheverry, Marc Aubry, Jean Mosser, Anan Yin, Xiang Zhang, Yuanming Wu, Kun Chen, Yalong He, Li Wang

**Affiliations:** ^1^Department of Biochemistry and Molecular Biology, Air Force Medical University, Xi'an, China; ^2^Department of Neurosurgery, Xijing Institute of Clinical Neuroscience, Xijing Hospital, Air Force Medical University, Xi'an, China; ^3^Institute of Neurosciences, Air Force Medical University, Xi'an, China; ^4^CNRS, UMR 6290, Institut de Génétique et Développement de Rennes (IGdR), Rennes F-35043, France; ^5^Department of Plastic and Reconstructive Surgery, Xijing Hospital, Air Force Medical University, Xi'an, China; ^6^Department of Anatomy, Histology and Embryology and K.K. Leung Brain Research Centre, Air Force Medical University, Xi'an, China; ^7^School of Aerospace Medicine, Air Force Medical University, Xi'an, China

## Abstract

**Objective:**

Alterations in the methylation state of pseudogenes may serve as clinically useful biomarkers of glioblastomas (GBMs) that do not have glioma-CpG island methylator phenotype (G-CIMP).

**Methods:**

Non-G-CIMP GBM datasets were included for evaluation, and a RISK-score signature was determined from the methylation state of pseudogene loci. Both bioinformatic and experimental analyses were performed for biological validation.

**Results:**

By integrating clinical information with DNA methylation microarray data, we screened a panel of eight CpGs from discovery cohorts of non-G-CIMP GBMs. Each CpG could accurately and independently predict the prognosis of patients under a treatment regime that combined radiotherapy (RT) and temozolomide (TMZ). The 8-CpG signature appeared to show opposite prognostic correlations between patients treated with RT/TMZ and those treated with RT monotherapy. The analyses further indicated that this signature had predictive value for TMZ efficacy because different survival benefits between RT/TMZ and RT therapies were observed in each risk subgroup. The incorporation of other risk factors, such as age and O-6-methylguanine-DNA methyltransferase (MGMT) promoter methylation status, with our pseudogene methylation signature could provide precise risk classification. In vitro experimental data revealed that two locus-specific pseudogenes (ZNF767P and CLEC4GP1) may modulate TMZ resistance via distinct mechanisms in GBM cells.

**Conclusion:**

The biologically and clinically relevant RISK-score signature, based on pseudogene methylation loci, may offer information for predicting TMZ responses of non-G-CIMP GBMs, that is independent from, but complementary to, MGMT-based approaches.

## 1. Introduction

Glioblastoma multiforme (GBM) with a glioma-CpGs island methylator phenotype (G-CIMP) are the most frequent and devastating glioma subtype [1]. Intra- or intertumoral molecular heterogeneity has been a major obstacle when developing treatment strategies against this deadly disease [2]. Identification of novel biologically and clinically relevant biomarkers may assist in the stratification of GBM subsets with distinct molecular features and allow for the development of precision medicines [2].

Compelling data have linked pseudogene alterations with glioma biology and response to treatment [3–5]. DNA methylation is a critical layer of control for the pseudogene transcriptome [6] and has long been regarded as an ideal cancer biomarker [7]; therefore, the identification of clinical relevant alterations in DNA methylation of pseudogenes is of great importance. In the present study, we integrate in silico and experimental approaches to examine the clinical and biological implications of pseudogene methylation in non-G-CIMP GBMs.

## 2. Methods

### 2.1. Patient Cohort from Rennes and Angers University Hospitals

A French patient cohort of seventy-seven primary non-G-CIMP GBMs from Rennes and Angers University Hospitals (RAUH) has previously been reported [7]. All patients underwent combination radiotherapy (RT) and concurrent and adjuvant temozolomide (TMZ). Snap-frozen surgical samples were profiled using Infinium Human Methylation450k BeadChip (Illumina Inc.) as described in Ref [7]. The G-CIMP subtype was determined by a K-means clustering algorithm [8], and the O-6-methylguanine-DNA methyltransferase (MGMT) promoter methylation status was calculated using DNA methylation data from two Illumina probes (cg12434587 and cg12981137) [9].

### 2.2. Patient Cohorts from Public Databases

Genomic DNA methylation and gene expression microarray data from 106 patients with an integrative diagnosis of non-G-CIMP GBM were downloaded from the Cancer Genome Atlas (TCGA) together with clinical annotations (RT/TMZ, *n* = 73; RT monotherapy, *n* = 13; and unknown regimens, *n* = 20) [10]. A further collection of 59 non-G-CIMP GBM samples with Illumina 450k DNA methylation microarray data was obtained from GSE60274 deposited in Gene Expression Omnibus (GEO; RT/TMZ, *n* = 32, and RT monotherapy, *n* = 27) [11]. Finally, Infinium450k DNA methylation microarray data from G-CIMP GBMs in TCGA [10] and nontumor brains (NTBs) in GSE63347 [12], together with RNA sequencing data from primary GBMs and NTBs in the Chinese Glioma Genome Atlas (CGGA) [13], were included for comparative analysis.

### 2.3. Probe Selection and RISK-Score Modeling

**Figure 1:**
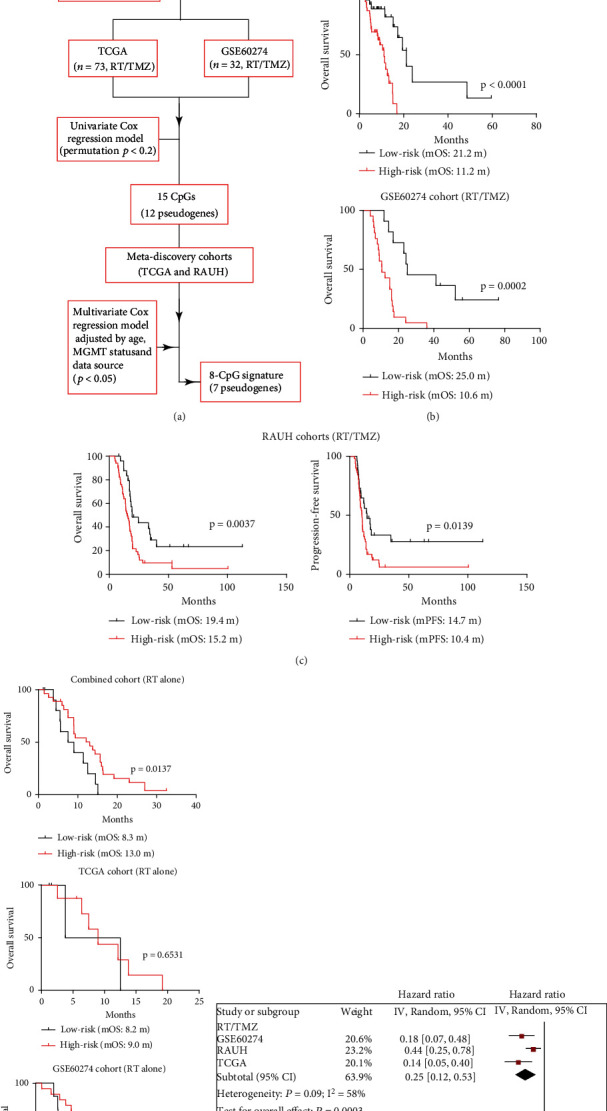
The discovery and validation of a pseudogene methylation signature for non-G-CIMP GBMs. (a) A multistep selection pipeline for identifying a clinically relevant pseudogene methylation signature; (b) risk classification by this signature in a combined discovery cohort of patients treated with RT/TMZ; (c) risk classification in an independent French cohort in term of OS and PFS; (d) risk classification in validation cohorts of patients treated with RT monotherapy; and (e) forest plots of comparison in OS: low-risk vs. high-risk tumors in patients with either RT/TMZ or RT alone.

A discovery-validation approach was employed to develop a multimarker prediction model. Data of patients treated with RT/TMZ were collected from TCGA and GSE60274 datasets for use during the discovery phase. Illumina 450k probes, that did not match regions with single-nucleotide polymorphisms and regions on X and Y chromosomes, were cross-matched with a list of 13603 pseudogenes downloaded from HGNC (HUGO Gene Nomenclature Committee;http://www.genenames.org/). 3210 CpGs located within the genomic regions of approximately 660 pseudogenes were identified and those with a standard deviation of inter value >0.1 from TCGA were further selected to correlate them with survival data using a univariate Cox regression model (Figure 1(a)). Inconsistent results from each discovery cohort were removed, and 15 overlapping candidates (permutation *P* < 0.2) were inputted into a multivariate Cox regression model that incorporated age, dataset source, and MGMT methylation status (Figure 1(a)). Finally, a panel of eight CpGs targeting seven pseudogenes was identified and combined using a RISK-score formula (Figure 1(a) and Table 1), which was calculated as the sum of *β* values of each CpG weighted by their multivariate Cox coefficients. The optimal cutoff for stratification of risk subgroups was determined using the maxstat R package [14]. Batch effect across datasets was adjusted using a nonparametric empirical Bayes approach (combat R package) [15].

### 2.4. Bioinformatic Analysis

Gene set enrichment analysis (GSEA) was performed on the gene sets of the gene ontology biological processes from molecular signature database (MSigDB) to evaluate the functional profiles of each risk subgroup [16]. Tumor mutation burden (TMB) was calculated using mutation annotation format (MAF) flies from TCGA and defined as the total amount of coding variants/the length of exons (38 million; maftools R package [17]).

### 2.5. Cell Culture and Drugs

The human GBM cell lines (A172, U251, U373, and DBTRG-05MG) were obtained from the American Type Culture Collection and were maintained in Dulbecco's modified Eagle's medium supplemented with 10% fetal bovine serum at 37°C in 5% CO_2_. TMZ (MedChemExpress) was reconstituted in dimethysulfoxide (DMSO, Sigma-Aldrich) at a concentration of 100 mM.

### 2.6. Quantitative Real-Time Polymerase Chain Reaction (qRT-PCR)

TRIzol reagent (Shanghai Pufei Biotech) was used for total RNA extraction. Total RNA was reverse transcribed using M-MLV RT kit (Promega). qRT-PCR was tested using SYBR Master Mixture (Takara) according to the manufacturers' protocol. The expression levels of targeted pesudogenes were normalized to GAPDH mRNA levels using the 2^−*ΔΔ*Ct^ method. The pesudogene-specific primers (Sangon Biotech) were listed as follows: GAPDH forward: 5′-TGACTTCAACAGCGACACCCA-3′; GAPDH reverse: 5′-CACCCTGTTGCTGTAGCCAAA-3′; ZNF767P forward: 5′-AAGCTGGCTGATTGCGAGAA-3′; ZNF767P reverse: 5′-GCAGTGGGAAAACCTCAGAGT-3′; CLEC4GP1 forward: 5′-CACTGGTTACAGGGGGAACG-3′; CLEC4GP1 reverse: 5′-TTGGCTAGGAGGAGAGGTGG-3′.

### 2.7. Cell Transfection

For in vitro knockdown of pseudogenes, small interfering (si) RNAs of CLEC4GP1 and ZNF767P as well as negative control were synthesized by Ribobio and transfected into GBM cells using X-tremeGENE siRNA Transfection Reagent (Roche) according to the manufacturers' protocol. The siRNA sequences were listed as follows: si-CLEC4GP1#1: CCACAGGTTAGACTCTAGA; si-CLEC4GP1#2: CCAGGCAATAAACAGGCTA; si-CLEC4GP1#3: ACACTAGTGCCCGTGAATA; si-ZNF767P#1: TCTCCTCTTTCCTCTAAAC; si-ZNF767P#2: GAATAGATGTCTCCCTATT; si-ZNF767P#3: CCATGTCTTGAATGTTTCT.

### 2.8. TMZ Cytotoxicity Assay

The chemosensitivity of TMZ was tested by the Cell Counting Kit-8 (CCK-8) kit. GBM cells were seeded in 96-cell plates at a density of 5 × 10^3^ cells per well and exposed at indicated concentrations of TMZ (7.5, 15, 30, 60, 120, 240, and 480 *μ*M) for 48 h. CCK-8 reagent was then added to wells (10 *μ*l/well) and incubated for 1 h at 37°C. The OD_450nm_ value was measured for calculating half maximal inhibitory concentration (IC50).

### 2.9. Western Blot Analysis

Cell lysates were performed in RIPA buffer contained protease inhibitor and phosphatase inhibitor (Roche). Cell lysates were subjected to sodium dodecyl sulfate (SDS)-polyacrylamide gel electrophoresis (PAGE) before transferring to polyvinylidene fluoride (PDVF) membranes (Millipore). Western blotting was performed with the following antibodies towards MGMT (Proteintech, 17195-1-AP), cleaved-PARP (CST, #5625), MPG (Proteintech, 11481-2-AP), p-p65 (CST, #3033), MLH1 (CST, #3515), MSH2 (CST, #2017), MSH6 (CST, #5424), and *β*-actin (CST, #3700). The expression of *β*-actin was used as the internal control. The intensity of bands was quantified using Quantity One software (Bio-Rad).

### 2.10. Statistical Analysis

Frequency data were examined using Fisher's exact or Chi-square test. Continuous data were examined using an unpaired *t* test or Mann–Whitney test. Time-to-event data (for example, overall survival (OS) or progression-free survival (PFS)) were compared using Kaplan-Meier curves and the log-rank test. The prognostic influence and independence of each variable were evaluated using univariate and multivariate Cox regression models. Results from each cohort or subgroup were pooled using meta-analysis, where an inverse-variance approach was applied using either fixed- or random effect models based on the heterogeneity test, with *P* < 0.1 or *I*^2^ > 50% considered to be statistically significant. Differences between subgroups were tested using subgroup analysis. The performance of risk variables was assessed using area under the curve (AUC) from a time-dependent receiver operating characteristic curve (survcomp R package) [18]. All statistical tests were done within SPSS (SPSS software Inc.) and R software. Statistical significance was defined as a two-sided *P* < 0.05.

## 3. Results

### 3.1. Identification of an 8-CpGs Signature Corresponding to Seven Pseudogenes in Non-G-CIMP GBMs

We screened a panel of eight CpGs within genomic regions of seven pesudogenes using a multistep selection strategy. Each locus predicted the OS of non-G-CIMP GBM patients treated with RT/TMZ, independent of age, dataset source, and MGMT promoter methylation status (Figure 1(a)). Genomic and clinical information from the identified CpGs and their methylation patterns in G-CIMP phenotype GBMs vs. NTBs is shown in Table 1 and Figure S1. A RISK-score signature was constructed for those eight CpGs as follows: RISK score = (1.584 × *β* value of cg18311708) + (1.909 × *β* value of cg22292345) + (1.635 × *β* value of cg24257776) + (−2.824 × *β* value of cg03534453) + (−2.306 × *β* value of cg07835270) + (−2.773 × *β* value of cg08409173) + (−2.604 × *β* value of cg19089383) + (−2.812 × *β* value of cg19500311). The optimal cutoff was calculated as -6.6904 for the combined discovery cohort.

### 3.2. The Performance of the Pseudogene Methylation Signature in Non-G-CIMP GBMs

Using the calculated cutoff above, we divided all patients from the discovery cohorts into low-risk and high-risk groups. Both pooled analysis of patient-level data and risk classification for each discovery cohort showed that OS of patients with high-risk tumors was significantly shorter than patients with low-risk tumors (Figure 1(b)). Similar results were observed for OS and PFS with an independent French cohort (Figure 1(c)). Cox regression analyses confirmed this signature as an independent risk indicator for RT/TMZ-treated patients (Table 2). Conversely, when RT monotherapy-treated cohorts were examined, we found that high-risk patients appeared to be associated with longer OS than low-risk patients (Figure 1(d)). Meta-analysis and Cox regression models both reported inverse prognostic correlations among patients with different treatments (Figure 1(e) and Table 2). These data suggested that our pseudogene methylation signature may not be a potent prognostic indicator for general GBM prognosis, but may be a predictive indicator for the survival benefits of additional TMZ treatment.

### 3.3. The Predictive Value of the Pseudogene Methylation Signature for TMZ Response

**Figure 2:**
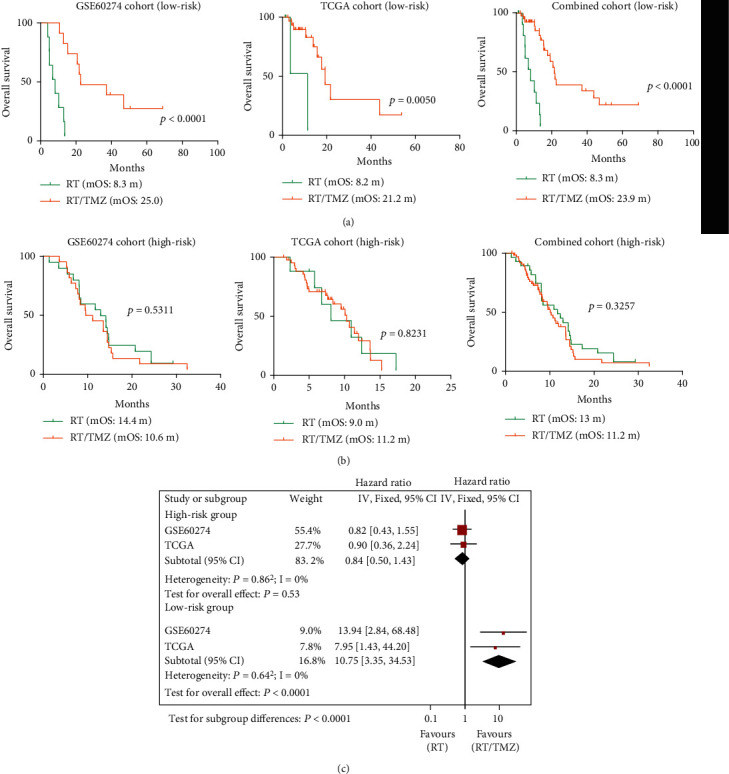
The performance of the pseudogene methylation signature for TMZ response. Survival comparison of patients treated with RT/TMZ vs. RT alone in (a) low-risk groups and (b) high-risk groups; and (c) forest plots of comparison in OS; RT/TMZ vs. RT monotherapy in patients with either low-risk or high-risk tumors.

To investigate whether our pseudogene methylation signature could provide predictive information about tumor response to TMZ, subgroup analyses were carried out between the risk and treatment subgroups. Patient baseline characteristics, such as age, gender, and MGMT methylation status, did not appear to differ from between above subgroups (data not shown). Subgroup analyses showed that low-risk patients benefited from RT/TMZ treatment over RT monotherapy (Figure 2(a)); however, OS differed very little between treatment types in high-risk patients (Figure 2(b)). Meta-analysis and Cox regression model confirmed those findings (Figure 2(c) and Table S1). The data indicated that our pseudogene methylation signature may predict TMZ efficacy and help identify patient subpopulations likely to benefit from TMZ treatment.

### 3.4. Patient Classification in Clinically and Molecularly Stratified Subcohorts

**Figure 3:**
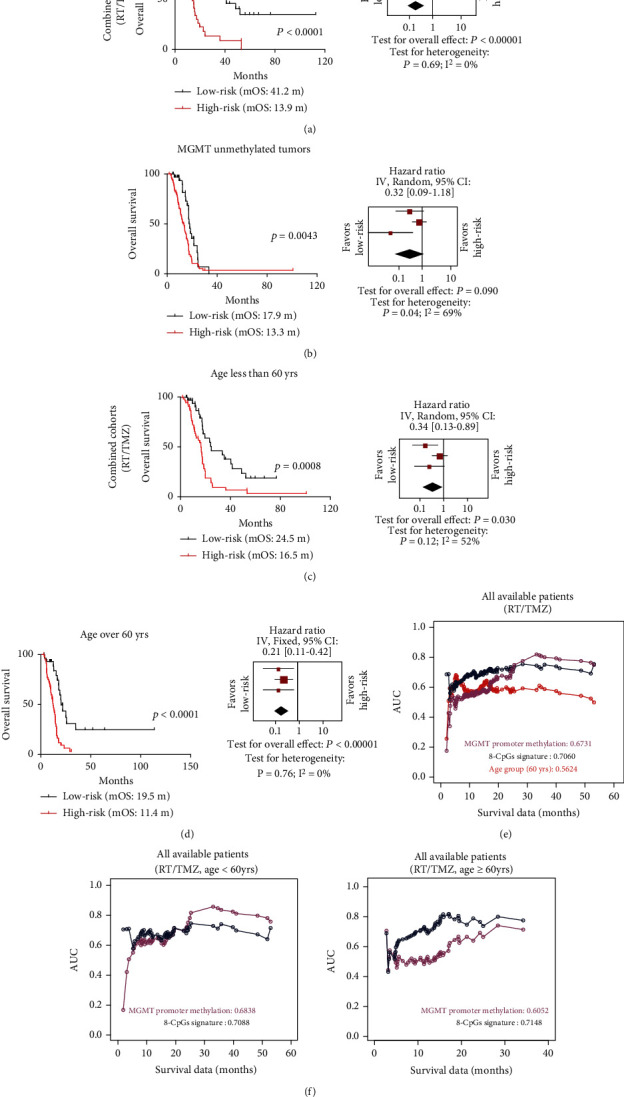
The risk classification of the pseudogene methylation signature in molecularly and clinically stratified cohorts. Risk classification and forest plots of comparison in OS for low-risk vs. high-risk patients from a combined cohort (TCGA, GSE60274, and RAUH collectively) who have (a) an MGMT methylated tumor, (b) an MGMT unmethylated tumor, (c) an age<60 years old or (d) an age ≥60 years old; and AUC comparison in (e) all available patients with RT/TMZ or in (f) all available RT/TMZ-treated patients with different ages.

To further evaluate the performance of the pseudogene methylation signature, we examined our risk classifications in a combined cohort from TCGA, GSE60274, and RAUH collectively, stratified by MGMT promoter methylation status and age. The 8-CpG signature was able to discriminate OS among patients with each MGMT promoter methylation status (Figures 3(a) and 3(b)) or within each age subgroup (Figures 3(c) and 3(d)). Moreover, AUC comparison showed that the signature had predictive value similar to MGMT promoter methylation status for patients of all ages treated with RT/TMZ and was superior to the MGMT-based approach for elderly populations (≥60 years; Figure 3(e)).

### 3.5. Clinical and Molecular Correlations of the Pseudogene Methylation Signature in TCGA Samples

**Figure 4:**
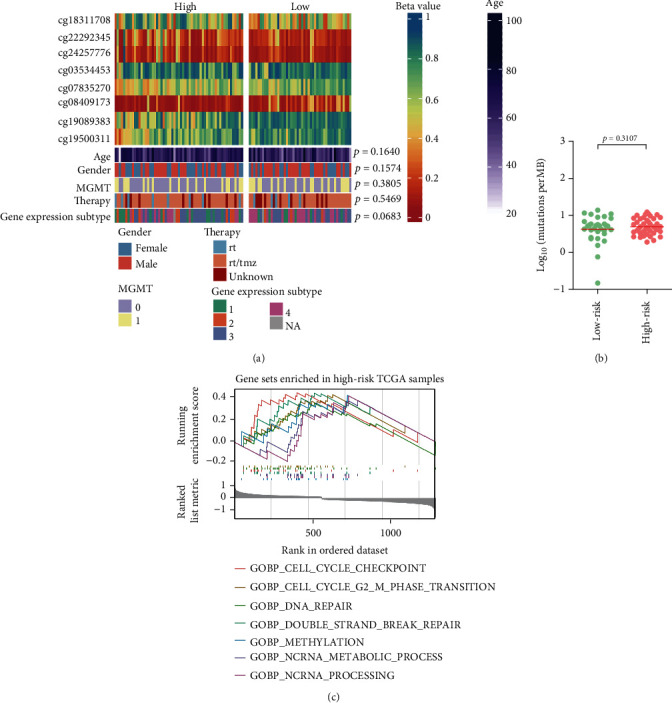
Molecular and clinical correlation of the pseudogene methylation signature in TCGA samples. (a) Heatmaps of clinical and molecular features; each row represented a feature, and each column represented a sample, which was ordered by the assigned risk scores; (b) comparison of TMB between low-risk vs. high-risk tumors; and (c) representative gene set highly enriched in high-risk tumors; statistical data for GSEA are presented in Table S2.

Correlation with known clinical and molecular features showed that the 8-CpG signature subgroups were not associated with age, gender, MGMT promoter methylation status, gene expression subtypes, and TMBs in TCGA samples (Figures 4(a) and 4(b)). GSEA on transcriptome data, however, revealed differential functional profiles between risk subgroups. In particular, high-risk tumors appeared to be more enriched for gene sets related to cell cycle regulation, DNA repair, and ncRNA processing (Figure 4(c) and Table S2).

### 3.6. A Preliminary Experimental Study of Two Pseudogenes on TMZ Resistance in GBM Cells

**Figure 5:**
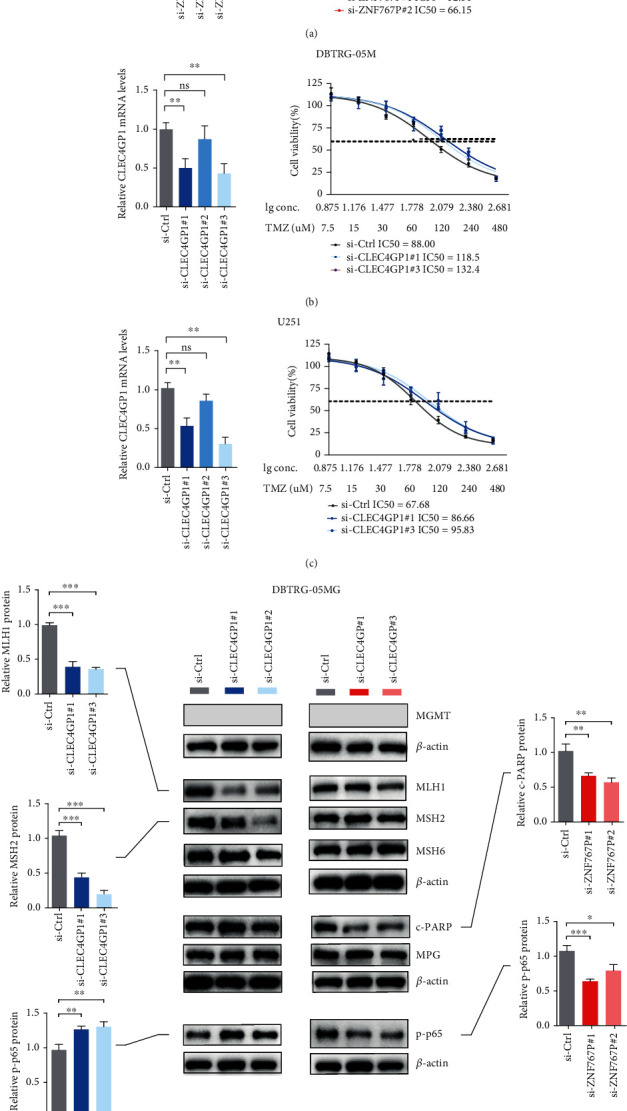
The impact of CLEC4GP1 and ZNF767P on TMZ resistance and relevant molecular alterations in GBM cells. IC50 of (a) DBTRG-05MG cells with siRNA knockdown of ZNF767P under the treatment of TMZ; IC50 of (b) DBTRG-05MG and (c) U251 cells with siRNA knockdown of CLEC4GP1 under the treatment of TMZ; and (d) protein levels of key components in different DNA repair pathways in DBTRG-05MG cells with siRNA knockdown of CLEC4GP1 and ZNF767P, respectively; ∗*P* < 0.05; ∗∗*P* < 0.01; ∗∗∗*P* < 0.001.

CGGA RNA sequencing data showed that two locus-specific pseudogenes were differentially expressed between GBMs with the wild-type isocitrate dehydrogenase gene (IDHwt), a surrogate marker for the G-CIMP phenotype, and NTBs; specifically, ZNF767P was found to be upregulated and CLEC4GP1 downregulated in IDHwt GBMs (Figure S2). Functional assays on these two pseudogenes showed that ZNF767P was relatively highly expressed in DBTRG-05MG cells and CLEC4GP1 in both DBTRG-05MG and U251 cells (Figure S3). TMZ cytotoxicity assays showed that siRNA knockdown of ZNF767P increased sensitivity to TMZ in DBTRG-05MG cells, while CLEC4GP1 knockdown decreased sensitivity to TMZ in both DBTRG-05MG and U251 cells (Figures 5(a)–5(c)). Western blot analysis showed that ZNF767P knockdown was associated with reduced activation of PAPR, a key mediator of base excision repair (BER) [19], while CLEC4GP1 knockdown resulted in decreased levels of mismatch repair (MMR) proteins such as MLH1 and MSH2 [19] (Figure 5(d)). Finally both pseudogenes had significant impacts on NF-*κ*B activation in DBTRG-05MG cells (Figure 5(d)). These data show that disruption of these two pseudogenes led to a disruption of the molecular mechanisms associated with TMZ resistance.

## 4. Discussion

Pseudogenes, characteristic by high sequence similarity to functional parent genes, have long been regarded as “junk DNA” due to the presence of a variety of disabling mutations (e.g., insertions, deletions, and stop codons) that result in loss of function [20]. The advent of large-scale, pan-cancer studies has prompted many to reexamine the function of pseudogenes, highlighting their multifaceted roles in cancer biology [3–5]. Integrative analysis of multiomics data showed that pseduogenes can be transcribed and translated, and that pseudogenic RNA and protein can regulate the function of key cancer genes, including their parent genes [3–5]. An increasing number of transcribed or translated pseudogenes have been discovered with diagnostic, prognostic, and predictive potentials in cancers [3–5]. Those pesudogene biomarkers have many disadvantages, namely, over standard biomarkers, due to issues inherent with the examination of expression data, such as unreliable RNA sampling and unstable altered patterns [21], and issues specific to the detection of pseudogene expression, such as complexities in designing reliable expression profiling approaches to distinguish the expression of parent gene with high sequence similarity, difficulties in defining which pseudogenes are transcribed, and a high dependence on the quality of reference genome sequences and annotation^3-5^.

Epigenetic marks and DNA methylation, in particular, represent critical layers of control of pseudogene transcription [22]. DNA methylation can, therefore, provide information about pseudogene transcription while avoiding issues with genetic sequence similarities. Pseudogene methylation information has many advantages over expression information, since DNA sampling is reliable, patterns are stable, and drug-induced changes are reversible [23]. Biomarkers that take advantage of pseduogene methylation patterns can often, therefore, be more practical and informative. In the present study, we constructed a RISK-score signature based on the methylation pattern of eight pseudogene-related CpGs by employing a multistep selection pipeline. The pseudogene methylation signature was found to exhibit opposite prognostic correlations for patients treated with RT/TMZ vs. RT monotherapy. Specifically, a high-risk score may be indicative of a poor outcome in RT/TMZ-treated patients, but a better outcome in those treated with RT alone. Subgroup analyses highlighted a predictive potential when making decisions about TMZ usage; the addition of TMZ appeared to be beneficial for low-risk but not high-risk counterparts. Correlation analyses indicated that the pseudogene methylation signature may not be an alternative manifestation of known clinical or molecular characteristics. Together, these data suggest that our pseudogene methylation signature may serve as a novel and promising predictive biomarker for TMZ response in non-G-CIMP GBMs, rather than as a general, treatment-independent prognostic biomarker [24].

The most informative biomarker for predicting TMZ outcome is the promoter methylation status of MGMT [25]. However, it is unlikely that TMZ would be withheld in patients with unmethylated MGMT promoters, since there is a lack of effective alternative therapies, and this agent has some benefits for this subpopulation [26]. Therefore, the use of MGMT promoter methylation as a biomarker for TMZ response may have limited value. The use of TMZ is also controversial for elderly subpopulations which are characterized by general poor health, reduced tolerance to anticancer therapy, and increased expectation for a better quality of life [27]. In fact, TMZ may not be a cost-effective option for GBM in health resource-limited countries, such as China [28]. The overuse of TMZ could result in overconsumption of health resources, raise medical cost to families and caregivers, and increase risk of drug toxicity. Selecting patients that are likely to respond well to TMZ and have favorable prognostic biomarkers may represent an effective approach for optimizing TMZ usage and increasing cost-effectiveness of treatment [26, 27]. The data reported here show that the pseudogene methylation signature could provide more refined risk classification in subpopulations determined by age or MGMT methylation status and may be helpful in identifying subsets of elderly patients, or patients with unmethylated tumors, most likely to benefit from TMZ treatment. We have shown that the pseudogene methylation signature has a better predictive power than the standard MGMT-based approach in elderly subpopulations. Taken together, our data highlights that the pseudogene methylation signature could be of use when optimizing patient selection, guiding treatment choice, and refining risk classification for non-G-CIMP GBMs. Despite the encouraging findings, the current signature biomarker is based on microarray data, and is yet not ready for routine clinical use due to the inaccessibility of high-throughput detection devices in daily clinical practice. Future studies are needed to exploit the microarray information in a common detection method, such as pyrosequencing.

Few studies have explored the biological implications of the pseudogenes harboring the identified CpGs: MT1DP, encoding metallothionein 1 (sub) isoforms [29], has been reported to have tumor suppressor roles in liver and lung cancers via RNA-RNA interactions [30]. PCDHB17P is highly expressed in breast cancers and promotes cancer by competing with endogenous RNA [31]. ZNF767P, a pseudogene on chromosome 7, has been reported to be differentially expressed in human cancers and to be translated when fused with oncogenic genes (e.g., BRAF) [32, 33]. CLEC4GP1 is a pseudogene of CLEC4G with unclear functions; its mRNA expression, however, has been reported to significantly correlate with the survival of GBM patients [34]. Little is known about the roles of the other three pseudogenes (NRADDP, ADCY10P1, and BMS1P4) in cancer.

Although DNA methylation is deemed to be a crucial regulator of pseudogene transcription, its epigenetic mechanisms and impact remain largely unclear, due to the complexity and specificity of pseudogenes in the genome [35]. DNA methylation may regulate the transcription of pseudogenes or genes adjacent to regions of pseudogene insertion [35]. Unfortunately, due to a lack of paired epigenetic and transcriptomic data, no direct evidence linking the pseudogene methylation panel and specific transcriptional alterations could be provided. Instead, two differentially expressed pseudogenes were selected for functional studies. ZNF767P was found to be upregulated and CLEC4GP1 downregulated in CGGA GBM samples. TMZ cytotoxicity increased the following ZNF767P knockdown in GBM cells, while knockdown of CLEC4GP1 decreased TMZ sensitivity. It is known that activation of NF-*κ*B signaling is one of the major molecular events associated with the TMZ resistance of GBM cells [36]. In addition, chemoresistance is also induced by dysfunctions of multiple DNA repair pathways, including MGMT, MMR, and BER [19]. Our data showed that both of the two pseudogenes affected NF-*κ*B activation, where each resulted in altered proteins involved in distinct DNA repair pathways, indicating that they may use different molecular mechanisms to modulate TMZ resistance. However, the current experimental data are too preliminary to draw firm conclusions. Future studies should explore the impacts of DNA methylation on the expression of these pseudogenes and examine more comprehensively the role played by these pseudogenes in glioma biology.

The following limitations should be noted when interpreting the findings of this study: (1) the Illumina 450k platform provides limited genomic coverage of pesudogenes; (2) the predictive value of our model has yet to be prospectively or retrospectively justified in a randomized setting; and (3) there are potential patient selection biases inherent in the retrospective study design, together with a small sample size for RT monotherapy-treated patients, the presence of nonstandard regimens, and incomplete clinical data.

In summary, we presented a preliminary report describing DNA methylation-based pesudogene signature as a biomarker in cancer. The multimarker signature may be useful for providing predictive information for outcome of TMZ in non-G-CIMP GBMs, independent of and complementary to the current MGMT-based approach.

## Figures and Tables

**Table 1:** Characteristics of the eight CpGs corresponding to seven pseudogenes.

Probe ID	Relevant pseudogene symbol	Chr.	Relation to gene region	Relation to CpGs island^a^	Multivariate Cox coefficients^a^
cg18311708	ZNF767P	7	TSS1500	Shore	1.584
cg22292345	NRADDP	3	TSS1500	Island	1.909
cg24257776	NRADDP	3	TSS1500	Island	1.635
cg03534453	PCDHB17P	5	Body	Shore	-2.824
cg07835270	MT1DP	16	TSS1500	Shore	-2.306
cg08409173	CLEC4GP1	19	Body	Island	-2.773
cg19089383	ADCY10P1	6	Body	Open Sea	-2.604
cg19500311	BMS1P4	10	Body	Shelf	-2.812

Chr = chromosome; TSS = transcriptional start site. ^a^Cox coefficients were calculated from multivariate analysis incorporating age, MGMT methylation status, and cohort source in meta-discovery cohorts of TCGA and GSE60274. ^b^Shore, shelf, and open sea referred to regions away from relevant CpGs islands less than 2000 base pairs, 2000~4000 base pairs, and more than 4000 base pairs, respectively.

**Table 2:** Univariate and multivariate Cox regression analyses in non-G-CIMP GBMs with RT/TMZ or RT monotherapy.

Variables	Univariate Cox model				Multivariate Cox model	
	HR	95% CI	*P* value	HR	95% CI	*P* value
Combined discovery cohorts (RT/TMZ)						
Patient age (increasing years)	1.037	1.011-1.063	*0.005*	1.037	1.012-1.063	*0.003*
The RISK-score signature (low vs. high)	0.199	0.106-0.372	*<0.001*	0.180	0.092-0.350	*<0.001*
MGMT methylation status (unmethylated vs. methylated)	2.203	1.232-3.938	*0.008*	1.826	0.994-3.355	0.052
Gene expression subtypes (nonproneural vs. proneural)	1.276	0.659-2.472	0.469			
Dataset source (TCGA vs. GSE60274)	1.223	0.729-2.052	0.446			
Combined discovery cohorts (RT monotherapy)						
Patient age (increasing years)	1.025	0.989-1.063	0.175			
The RISK-score signature (low vs. high)	2.325	1.047-5.166	*0.038*			
MGMT methylation status (unmethylated vs. methylated)	1.274	0.650-2.497	0.480			
Gene expression subtypes (nonproneural vs. proneural)	1.033	0.416-2.569	0.944			
Dataset source (TCGA vs. GSE60274)	1.689	0.773-3.693	0.189			
RAUH cohort (RT/TMZ)						
Patient age (increasing years)	1.032	1.003-1.062	*0.029*	1.035	1.002-1.069	*0.039*
Pre-adjuvant therapy KPS (≤ 70 vs. >70)	1.319	0.602-2.887	0.489			
Extent of surgery (biopsy vs. partial vs. total)	1.034	0.689-1.550	0.872			
The RISK-score signature (low vs. high)	0.441	0.249-0.779	*0.005*	0.528	0.285-0.981	*0.043*
TERT promoter mutation (no vs. yes)	0.367	0.144-0.932	*0.035*	0.500	0.178-1.404	0.188
MGMT methylation status (unmethylated vs. methylated)	2.423	1.334-4.401	*0.004*	2.685	1.366-5.277	*0.004*
Gene expression subtypes (nonproneural vs. proneural)	1.040	0.569-1.898	0.889			

RAUH = Rennes and Angers University Hospitals; TCGA = the Cancer Genome Atlas; G-CIMP = glioma-CpGs island methylator phenotype; MGMT = the O-6-methylguanine-DNA methyltransferase; GBM = glioblastoma; KPS = Karnofsky performance score; TMZ = temozolomide; RT = radiotherapy; TERT = telomerase reverse tranase. Italics were significant results.

## Data Availability

The datasets used and/or analyzed during the current study are available from the corresponding author on reasonable request or public databases; TCGA: https://tcga-data.nci.nih.gov; CGGA: http://www.cgga.org.cn/; GEO: https://www.ncbi.nlm.nih.gov/geo/.

## Abbreviations

AUC: Area under the curve
BER: Base excision repair
CCK-8: Cell counting Kit-8
CCGA: Chinese Glioma Genome Atlas
DMSO: Dimethysulfoxide
GBMs: Glioblastomas
G-CIMP: Glioma-CpGs island methylator phenotype
GEO: Gene expression omnibus
GSEA: Gene set enrichment analysis
IDHwt: Wild-type isocitrate dehydrogenase
MAF: Mutation annotation format
MMR: Mismatch repair
MGMT: O-6-methylguanine-DNA methyltransferase
MSigDB: Molecular signatures database
NTBs: Nontumor brains
OS: Overall survival
PAGE: Polyacrylamide gel electrophoresis
PDVF: Polyvinylidene fluoride
PFS: Progression-free survival
qRT-PCR: Quantitative real-time polymerase chain reaction
RAUH: Rennes and Angers University Hospitals
RT: Radiotherapy
SDS: Sodium dodecyl sulfate
TCGA: The Cancer Genome Atlas
TERT: Telomerase reverse tranase
TMB: Tumor mutation burden
TMZ: Temozolomide.

## Supplementary Materials

Supplementary Materials Supplementary Materials Figure S1: Comparison of the methylation states of the 8 CpGs between GBMs of each G-CIMP phenotype and NTBs; NTBs were obtained from GSE63347 and GBMs of each G-CIMP phenotype from TCGA; NTB = nontumor brains; GBM = glioblastoma; and G-CIMP = glioma CpGs island methylator phenotype. Figure S2: Comparison of the expression states of the 5 available pseudogenes between GBMs of each G-CIMP phenotype and NTBs from CGGA; GBM = glioblastoma; G-CIMP = glioma CpGs island methylator phenotype; and CGGA = China Glioma Genome Atlas. Figure S3: Transcriptional levels of CLEC4GP1 and ZNF767P in common GBM cell lines. Table S1: Univariate and multivariate Cox regression analyses in low-risk group of non-G-CIMP GBMs with RT/TMZ or RT alone. Table S2: GSEA analysis of low-risk and high-risk non-CIMP GBMs from TCGA.
