# Supplementary material for: A Novel Pseudogene Methylation Signature to Predict Temozolomide Outcome in Non-G-CIMP Glioblastomas
Source: J Oncol. 2022 Jun 6;2022:6345160. doi: 10.1155/2022/6345160 (PMC9194959; doi:10.1155/2022/6345160)
Supplement: Supplementary Materials — Figure S1: Comparison of the methylation states of the 8 CpGs between GBMs of each G-CIMP phenotype and NTBs; NTBs were obtained from GSE63347 and GBMs of each G-CIMP phenotype from TCGA; NTB = nontumor brains; GBM = glioblastoma; and G-CIMP = glioma CpGs island methylator phenotype. Figure S2: Comparison of the expression states of the 5 available pseudogenes between GBMs of each G-CIMP phenotype and NTBs from CGGA; GBM = glioblastoma; G-CIMP = glioma CpGs island methylator phenotype; and CGGA = China Glioma Genome Atlas. Figure S3: Transcriptional levels of CLEC4GP1 and ZNF767P in common GBM cell lines. Table S1: Univariate and multivariate Cox regression analyses in low-risk group of non-G-CIMP GBMs with RT/TMZ or RT alone. Table S2: GSEA analysis of low-risk and high-risk non-CIMP GBMs from TCGA. [file 6345160.f5.zip › 6345160.f1.docx]

| **Table S1: Univariate and multivariate Cox regression analyses in each risk group of non-G-CIMP GBMs** | | | | | | | |
| --- | --- | --- | --- | --- | --- | --- | --- |
| Variables | Univariate Cox model | | |  | Multivariate Cox model | | |
|  | HR | 95% CI | P value | | HR | 95% CI | P value |
| **Combined discovery cohorts (low-risk)** |  |  |  |  |  |  |  |
| Patient age (increasing years) | 1.059 | 1.012-1.107 | **0.01** |  | 1.054 | 1.004-1.107 | **0.034** |
| *MGMT* methylation status (unmethylated vs. methylated) | 1.571 | 0.688-3.587 | 0.284 |  |  |  |  |
| Treatment (RT vs. RT+TMZ) | 10.294 | 3.660-28.953 | **<0.001** |  | 9.271 | 3.250-26.449 | **<0.001** |
| Gene expression subtypes (Non-proneural vs. Proneural) | 1.301 | 0.482-3.515 | 0.603 |  |  |  |  |
| Dataset source (TCGA vs. GSE60274) | 0.864 | 0.400-1.869 | 0.711 |  |  |  |  |
| **Combined discovery cohorts (high-risk)** |  |  |  |  |  |  |  |
| Patient age (increasing years) | 1.03 | 1.007-1.053 | **0.010** |  |  |  |  |
| *MGMT* methylation status (unmethylated vs. methylated) | 1.61 | 0.959-2.704 | 0.072 |  |  |  |  |
| Treatment (RT vs. RT+TMZ) | 0.778 | 0.470-1.289 | 0.331 |  |  |  |  |
| Gene expression subtypes (Non-proneural vs. Proneural) | 1.157 | 0.615-2.178 | 0.651 |  |  |  |  |
| Dataset source (TCGA vs. GSE60274) | 1.661 | 0.992-2.781 | 0.054 |  |  |  |  |

TCGA=The Cancer Genome Atlas; G-CIMP=glioma-CpGs island methylator phenotype; MGMT= the O-6-methylguanine-DNA methyltransferase; GBM=glioblastoma; TMZ=temozolomide; RT=radiotherapy;

**In bold were significant results**
