# Supplementary material for: A Novel Pseudogene Methylation Signature to Predict Temozolomide Outcome in Non-G-CIMP Glioblastomas
Source: J Oncol. 2022 Jun 6;2022:6345160. doi: 10.1155/2022/6345160 (PMC9194959; doi:10.1155/2022/6345160)
Supplement: Supplementary Materials — Figure S1: Comparison of the methylation states of the 8 CpGs between GBMs of each G-CIMP phenotype and NTBs; NTBs were obtained from GSE63347 and GBMs of each G-CIMP phenotype from TCGA; NTB = nontumor brains; GBM = glioblastoma; and G-CIMP = glioma CpGs island methylator phenotype. Figure S2: Comparison of the expression states of the 5 available pseudogenes between GBMs of each G-CIMP phenotype and NTBs from CGGA; GBM = glioblastoma; G-CIMP = glioma CpGs island methylator phenotype; and CGGA = China Glioma Genome Atlas. Figure S3: Transcriptional levels of CLEC4GP1 and ZNF767P in common GBM cell lines. Table S1: Univariate and multivariate Cox regression analyses in low-risk group of non-G-CIMP GBMs with RT/TMZ or RT alone. Table S2: GSEA analysis of low-risk and high-risk non-CIMP GBMs from TCGA. [file 6345160.f5.zip › 6345160.f5.pdf]

Gene sets	setSize	enrichmentScore	NES	pvalue	p.adjust	qvalues	rank
GOBP_EXTERNAL_ENCAPSULATING_STRUCTURE_ORGANIZATION	62.00	-0.60	-3.03	0.00	0.00	0.00	449.00
GOBP_VASCULATURE_DEVELOPMENT	104.00	-0.51	-2.84	0.00	0.00	0.00	526.00
GOBP_BLOOD_VESSEL_MORPHOGENESIS	95.00	-0.51	-2.80	0.00	0.00	0.00	526.00
GOBP_ANATOMICAL_STRUCTURE_FORMATION_INVOLVED_IN_MORPHOGENESIS	125.00	-0.47	-2.71	0.00	0.00	0.00	416.00
GOBP_CELL_MIGRATION	152.00	-0.45	-2.69	0.00	0.00	0.00	544.00
GOBP_CIRCULATORY_SYSTEM_PROCESS	75.00	-0.51	-2.69	0.00	0.00	0.00	444.00
GOBP_CIRCULATORY_SYSTEM_DEVELOPMENT	137.00	-0.46	-2.68	0.00	0.00	0.00	526.00
GOBP_LOCOMOTION	172.00	-0.44	-2.65	0.00	0.00	0.00	552.00
GOBP_RESPONSE_TO_BACTERIUM	68.00	-0.51	-2.65	0.00	0.00	0.00	477.00
GOBP_BIOLOGICAL_ADHESION	179.00	-0.43	-2.64	0.00	0.00	0.00	552.00
GOBP_TUBE_MORPHOGENESIS	110.00	-0.46	-2.62	0.00	0.00	0.00	526.00
GOBP_REGULATION_OF_VASCULATURE_DEVELOPMENT	46.00	-0.56	-2.60	0.00	0.00	0.00	526.00
GOBP_POSITIVE_REGULATION_OF_LOCOMOTION	68.00	-0.50	-2.59	0.00	0.00	0.00	544.00
GOBP_REGULATION_OF_BLOOD_PRESSURE	20.00	-0.69	-2.56	0.00	0.00	0.00	244.00
GOBP_TUBE_DEVELOPMENT	125.00	-0.44	-2.54	0.00	0.00	0.00	526.00
GOBP_EPITHELIAL_CELL_PROLIFERATION	51.00	-0.53	-2.52	0.00	0.00	0.00	411.00
GOBP_POSITIVE_REGULATION_OF_CELL_POPULATION_PROLIFERATION	100.00	-0.45	-2.51	0.00	0.00	0.00	416.00
GOBP_REGULATION_OF_CELLULAR_COMPONENT_MOVEMENT	109.00	-0.44	-2.47	0.00	0.00	0.00	544.00
GOBP_CELL_SUBSTRATE_ADHESION	54.00	-0.51	-2.46	0.00	0.00	0.00	416.00
GOBP_VASCULAR_PROCESS_IN_CIRCULATORY_SYSTEM	33.00	-0.57	-2.45	0.00	0.00	0.00	347.00
GOBP_INFLAMMATORY_RESPONSE	94.00	-0.44	-2.44	0.00	0.00	0.00	419.00
GOBP_REGULATION_OF_CELL_ADHESION	91.00	-0.44	-2.43	0.00	0.00	0.00	575.00
GOBP_REGULATION_OF_TUBE_SIZE	18.00	-0.66	-2.36	0.00	0.00	0.00	347.00
GOBP_DEFENSE_RESPONSE	181.00	-0.38	-2.35	0.00	0.00	0.00	454.00
GOBP_LEUKOCYTE_MIGRATION	59.00	-0.47	-2.35	0.00	0.00	0.00	411.00
GOBP_AMEBOIDAL_TYPE_CELL_MIGRATION	50.00	-0.50	-2.35	0.00	0.00	0.00	534.00
GOBP_CELL_POPULATION_PROLIFERATION	202.00	-0.38	-2.35	0.00	0.00	0.00	529.00
GOBP_CELL_MATRIX_ADHESION	36.00	-0.53	-2.34	0.00	0.00	0.00	411.00
GOBP_RESPONSE_TO_BIOTIC_STIMULUS	151.00	-0.39	-2.33	0.00	0.00	0.00	531.00
GOBP_REGULATION_OF_CELL_DEATH	166.00	-0.38	-2.31	0.00	0.00	0.00	424.00
GOBP_NEGATIVE_REGULATION_OF_EXTRINSIC_APOPTOTIC_SIGNALING_PATHWAY	14.00	-0.70	-2.30	0.00	0.00	0.00	356.00
GOBP_SECRETION	147.00	-0.39	-2.30	0.00	0.00	0.00	449.00
GOBP_RESPONSE_TO_MOLECULE_OF_BACTERIAL_ORIGIN	40.00	-0.51	-2.29	0.00	0.00	0.00	492.00
GOBP_RESPONSE_TO_WOUNDING	74.00	-0.43	-2.29	0.00	0.00	0.00	508.00
GOBP_POSITIVE_REGULATION_OF_MULTICELLULAR_ORGANISMAL_PROCESS	154.00	-0.38	-2.29	0.00	0.00	0.00	526.00
GOBP_TAXIS	66.00	-0.45	-2.28	0.00	0.00	0.00	357.00
GOBP_TISSUE_MIGRATION	41.00	-0.50	-2.28	0.00	0.00	0.00	526.00
GOBP_WOUND_HEALING	63.00	-0.45	-2.27	0.00	0.00	0.00	508.00
GOBP_ACUTE_INFLAMMATORY_RESPONSE	22.00	-0.60	-2.27	0.00	0.00	0.00	189.00
GOBP_REGULATION_OF_EXTRINSIC_APOPTOTIC_SIGNALING_PATHWAY	20.00	-0.61	-2.26	0.00	0.00	0.00	306.00
GOBP_POSITIVE_REGULATION_OF_CELL_ADHESION	56.00	-0.46	-2.26	0.00	0.00	0.00	570.00

GOBP_CYTOKINE_PRODUCTION	82.00	-0.42	-2.26	0.00	0.00	0.00	526.00
GOBP_ENDOTHELIAL_CELL_MIGRATION	34.00	-0.52	-2.25	0.00	0.00	0.00	498.00
GOBP_POSITIVE_REGULATION_OF_EPITHELIAL_CELL_PROLIFERATION	21.00	-0.60	-2.24	0.00	0.00	0.00	411.00
GOBP_PEPTIDE_SECRETION	34.00	-0.51	-2.21	0.00	0.00	0.00	354.00
GOBP_VASOCONSTRICTION	12.00	-0.70	-2.21	0.00	0.00	0.00	347.00
GOBP_GRANULOCYTE_CHEMOTAXIS	16.00	-0.64	-2.21	0.00	0.00	0.00	174.00
GOBP_TRANSMEMBRANE_RECEPTOR_PROTEIN_TYROSINE_KINASE_SIGNALING_PATHWAY	71.00	-0.42	-2.20	0.00	0.00	0.00	550.00
GOBP_ENDOTHELIAL_CELL_PROLIFERATION	26.00	-0.55	-2.20	0.00	0.00	0.00	355.00
GOBP_MYELOID_LEUKOCYTE_ACTIVATION	80.00	-0.41	-2.20	0.00	0.00	0.00	512.00
GOBP_NEGATIVE_REGULATION_OF_PEPTIDASE_ACTIVITY	19.00	-0.61	-2.20	0.00	0.00	0.00	340.00
GOBP_NEGATIVE_REGULATION_OF_VASCULATURE_DEVELOPMENT	19.00	-0.61	-2.20	0.00	0.00	0.00	547.00
GOBP_RESPONSE_TO_CYTOKINE	133.00	-0.37	-2.19	0.00	0.00	0.00	653.00
GOBP_MUSCLE_SYSTEM_PROCESS	49.00	-0.46	-2.19	0.00	0.00	0.00	488.00
GOBP_MYELOID_LEUKOCYTE_MEDIATED_IMMUNITY	66.00	-0.43	-2.19	0.00	0.00	0.00	447.00
GOBP_POSITIVE_REGULATION_OF_VASCULATURE_DEVELOPMENT	27.00	-0.54	-2.17	0.00	0.00	0.00	526.00
GOBP_CELL_ADHESION_MEDIATED_BY_INTEGRIN	13.00	-0.67	-2.17	0.00	0.00	0.00	409.00
GOBP_REGULATION_OF_MULTICELLULAR_ORGANISMAL_DEVELOPMENT	141.00	-0.37	-2.16	0.00	0.00	0.00	574.00
GOBP_CELL_ACTIVATION	164.00	-0.36	-2.16	0.00	0.00	0.00	576.00
GOBP_IMMUNE_EFFECTOR_PROCESS	128.00	-0.37	-2.15	0.00	0.00	0.00	454.00
GOBP_NEGATIVE_REGULATION_OF_APOPTOTIC_SIGNALING_PATHWAY	24.00	-0.55	-2.14	0.00	0.01	0.00	306.00
GOBP_LEUKOCYTE_CHEMOTAXIS	28.00	-0.53	-2.14	0.00	0.00	0.00	357.00
GOBP_GRANULOCYTE_MIGRATION	19.00	-0.59	-2.14	0.00	0.00	0.00	174.00
GOBP_EXTRINSIC_APOPTOTIC_SIGNALING_PATHWAY	35.00	-0.49	-2.13	0.00	0.00	0.00	378.00
GOBP_MUSCLE_CONTRACTION	45.00	-0.46	-2.13	0.00	0.00	0.00	488.00
GOBP_ACUTE_PHASE_RESPONSE	10.00	-0.72	-2.13	0.00	0.00	0.00	85.00
GOBP_APOPTOTIC_PROCESS	178.00	-0.35	-2.12	0.00	0.00	0.00	424.00
GOBP_REGULATION_OF_PEPTIDYL_TYROSINE_PHOSPHORYLATION	31.00	-0.50	-2.12	0.00	0.00	0.00	540.00
GOBP_HUMORAL_IMMUNE_RESPONSE	24.00	-0.55	-2.12	0.00	0.01	0.00	242.00
GOBP_CYTOKINE_MEDIATED_SIGNALING_PATHWAY	99.00	-0.38	-2.11	0.00	0.00	0.00	667.00
GOBP_NEGATIVE_REGULATION_OF_CELL_POPULATION_PROLIFERATION	76.00	-0.40	-2.11	0.00	0.00	0.00	286.00
GOBP_SENSORY_ORGAN_MORPHOGENESIS	21.00	-0.56	-2.11	0.00	0.00	0.00	345.00
GOBP_SMOOTH_MUSCLE_CELL_PROLIFERATION	23.00	-0.54	-2.10	0.00	0.01	0.00	507.00
GOBP_MYELOID_LEUKOCYTE_MIGRATION	28.00	-0.52	-2.10	0.00	0.00	0.00	357.00
GOBP_POSITIVE_REGULATION_OF_DEVELOPMENTAL_PROCESS	127.00	-0.36	-2.10	0.00	0.00	0.00	550.00
GOBP_MULTICELLULAR_ORGANISMAL_HOMEOSTASIS	52.00	-0.44	-2.10	0.00	0.00	0.00	444.00
GOBP_PHAGOCYTOSIS	28.00	-0.52	-2.10	0.00	0.00	0.00	396.00
GOBP_CELL_CHEMOTAXIS	38.00	-0.47	-2.09	0.00	0.00	0.00	357.00
GOBP_ENZYME_LINKED_RECEPTOR_PROTEIN_SIGNALING_PATHWAY	108.00	-0.37	-2.09	0.00	0.00	0.00	541.00
GOBP_NEGATIVE_REGULATION_OF_CELL_DEATH	104.00	-0.37	-2.09	0.00	0.00	0.00	422.00
GOBP_LEUKOCYTE_APOPTOTIC_PROCESS	14.00	-0.63	-2.09	0.00	0.01	0.01	409.00
GOBP_REGULATION_OF_EPITHELIAL_CELL_MIGRATION	32.00	-0.49	-2.08	0.00	0.00	0.00	526.00
GOBP_REGULATION_OF_ANATOMICAL_STRUCTURE_MORPHOGENESIS	99.00	-0.37	-2.08	0.00	0.00	0.00	550.00

GOBP_REGULATION_OF_APOPTOTIC_SIGNALING_PATHWAY	33.00	-0.49	-2.08	0.00	0.00	0.00	306.00
GOBP_POSITIVE_REGULATION_OF_CHEMOTAXIS	20.00	-0.56	-2.07	0.00	0.01	0.01	507.00
GOBP_EPITHELIAL_CELL_APOPTOTIC_PROCESS	17.00	-0.59	-2.07	0.00	0.01	0.01	574.00
GOBP_NEGATIVE_REGULATION_OF_EPITHELIAL_CELL_PROLIFERATION	17.00	-0.58	-2.06	0.00	0.01	0.01	344.00
GOBP_REGULATION_OF_LEUKOCYTE_APOPTOTIC_PROCESS	12.00	-0.66	-2.06	0.00	0.01	0.01	409.00
GOBP_ACTIN_FILAMENT_BASED_PROCESS	85.00	-0.38	-2.06	0.00	0.00	0.00	429.00
GOBP_NEUTROPHIL_CHEMOTAXIS	14.00	-0.62	-2.06	0.00	0.01	0.01	63.00
GOBP_FORMATION_OF_PRIMARY_GERM_LAYER	13.00	-0.63	-2.05	0.00	0.01	0.00	408.00
GOBP_DEFENSE_RESPONSE_TO_OTHER_ORGANISM	114.00	-0.36	-2.05	0.00	0.00	0.00	531.00
GOBP_EMBRYONIC_MORPHOGENESIS	57.00	-0.41	-2.05	0.00	0.00	0.00	312.00
GOBP_CELLULAR_RESPONSE_TO_MOLECULE_OF_BACTERIAL_ORIGIN	24.00	-0.53	-2.05	0.00	0.01	0.01	526.00
GOBP_EPITHELIAL_CELL_DIFFERENTIATION	57.00	-0.41	-2.05	0.00	0.00	0.00	540.00
GOBP_REGULATION_OF_ENDOTHELIAL_CELL_MIGRATION	25.00	-0.52	-2.05	0.00	0.01	0.01	526.00
GOBP_POSITIVE_REGULATION_OF_PEPTIDYL_TYROSINE_PHOSPHORYLATION	25.00	-0.52	-2.04	0.00	0.01	0.01	454.00
GOBP_DEFENSE_RESPONSE_TO_BACTERIUM	25.00	-0.52	-2.04	0.00	0.01	0.01	407.00
GOBP_REGULATION_OF_PEPTIDE_SECRETION	25.00	-0.52	-2.04	0.00	0.01	0.01	436.00
GOBP_VASCULAR_ENDOTHELIAL_GROWTH_FACTOR_RECEPTOR_SIGNALING_PATHWAY	12.00	-0.65	-2.04	0.00	0.01	0.01	408.00
GOBP_SUPRAMOLECULAR_FIBER_ORGANIZATION	65.00	-0.40	-2.03	0.00	0.00	0.00	541.00
GOBP_MULTI_MULTICELLULAR_ORGANISM_PROCESS	17.00	-0.58	-2.03	0.00	0.01	0.01	183.00
GOBP_NEGATIVE_REGULATION_OF_MULTICELLULAR_ORGANISMAL_PROCESS	108.00	-0.36	-2.03	0.00	0.00	0.00	547.00
GOBP_NEUTROPHIL_MIGRATION	17.00	-0.57	-2.02	0.00	0.01	0.01	63.00
GOBP_SENSORY_ORGAN_DEVELOPMENT	66.00	-0.40	-2.02	0.00	0.00	0.00	399.00
GOBP_EPITHELIAL_CELL_DEVELOPMENT	29.00	-0.50	-2.02	0.00	0.01	0.01	540.00
GOBP_POSITIVE_REGULATION_OF_INTRACELLULAR_SIGNAL_TRANSDUCTION	102.00	-0.36	-2.01	0.00	0.00	0.00	586.00
GOBP_REGULATION_OF_EPITHELIAL_CELL_APOPTOTIC_PROCESS	16.00	-0.59	-2.01	0.00	0.01	0.01	574.00
GOBP_ACTIN_FILAMENT_BASED_MOVEMENT	20.00	-0.54	-2.01	0.00	0.01	0.01	429.00
GOBP_POSITIVE_REGULATION_OF_CYTOKINE_PRODUCTION	51.00	-0.42	-2.01	0.00	0.01	0.01	616.00
GOBP_CELL_ACTIVATION_INVOLVED_IN_IMMUNE_RESPONSE	84.00	-0.37	-2.00	0.00	0.00	0.00	454.00
GOBP_NEGATIVE_REGULATION_OF_DEVELOPMENTAL_PROCESS	73.00	-0.38	-2.00	0.00	0.00	0.00	551.00
GOBP_SMOOTH_MUSCLE_CONTRACTION	17.00	-0.57	-2.00	0.00	0.02	0.01	347.00
GOBP_CELLULAR_RESPONSE_TO_BIOTIC_STIMULUS	27.00	-0.50	-2.00	0.00	0.01	0.01	526.00
GOBP_REGULATION_OF_PEPTIDASE_ACTIVITY	42.00	-0.44	-2.00	0.00	0.01	0.01	443.00
GOBP_NEGATIVE_REGULATION_OF_RESPONSE_TO_STIMULUS	162.00	-0.33	-1.99	0.00	0.00	0.00	551.00
GOBP_NEGATIVE_REGULATION_OF_SECRETION	15.00	-0.58	-1.98	0.00	0.03	0.02	449.00
GOBP_SKIN_DEVELOPMENT	24.00	-0.51	-1.96	0.00	0.02	0.02	192.00
GOBP_LEUKOCYTE_MEDIATED_IMMUNITY	91.00	-0.36	-1.96	0.00	0.00	0.00	454.00
GOBP_HOMEOSTATIC_PROCESS	171.00	-0.32	-1.95	0.00	0.00	0.00	405.00
GOBP_POSITIVE_REGULATION_OF_SIGNALING	165.00	-0.32	-1.95	0.00	0.00	0.00	416.00
GOBP_POSITIVE_REGULATION_OF_ENDOTHELIAL_CELL_PROLIFERATION	14.00	-0.59	-1.95	0.00	0.02	0.02	411.00
GOBP_BLOOD_VESSEL_ENDOTHELIAL_CELL_MIGRATION	21.00	-0.52	-1.95	0.00	0.01	0.01	498.00
GOBP_ANIMAL_ORGAN_MORPHOGENESIS	111.00	-0.34	-1.95	0.00	0.00	0.00	355.00

GOBP_MUSCLE_CELL_PROLIFERATION	29.00	-0.48	-1.94	0.00	0.01	0.01	507.00
GOBP_EPITHELIUM_DEVELOPMENT	108.00	-0.34	-1.94	0.00	0.00	0.00	359.00
GOBP_POSITIVE_REGULATION_OF_CELL_CELL_ADHESION	37.00	-0.44	-1.94	0.00	0.02	0.01	570.00
GOBP_CELL_SUBSTRATE_JUNCTION_ORGANIZATION	17.00	-0.55	-1.94	0.00	0.02	0.02	466.00
GOBP_POSITIVE_REGULATION_OF_GENE_EXPRESSION	95.00	-0.35	-1.93	0.00	0.00	0.00	526.00
GOBP_ANTIMICROBIAL_HUMORAL_RESPONSE	11.00	-0.64	-1.93	0.00	0.02	0.01	329.00
GOBP_PEPTIDYL_TYROSINE_MODIFICATION	44.00	-0.42	-1.93	0.00	0.01	0.00	540.00
GOBP_POSITIVE_REGULATION_OF_EPITHELIAL_CELL_MIGRATION	21.00	-0.51	-1.92	0.00	0.02	0.01	526.00
GOBP_AGING	41.00	-0.42	-1.92	0.00	0.03	0.02	183.00
GOBP_POSITIVE_REGULATION_OF_SMOOTH_MUSCLE_CELL_PROLIFERATION	11.00	-0.63	-1.92	0.00	0.02	0.01	507.00
GOBP_VIRAL_LIFE_CYCLE	33.00	-0.45	-1.92	0.00	0.02	0.01	434.00
GOBP_REGULATION_OF_ANATOMICAL_STRUCTURE_SIZE	54.00	-0.39	-1.91	0.00	0.01	0.01	406.00
GOBP_POSITIVE_REGULATION_OF_IMMUNE_SYSTEM_PROCESS	101.00	-0.34	-1.91	0.00	0.01	0.00	617.00
GOBP_SENSORY_SYSTEM_DEVELOPMENT	51.00	-0.40	-1.91	0.00	0.01	0.01	394.00
GOBP_RESPONSE_TO_INTERFERON_GAMMA	27.00	-0.47	-1.90	0.00	0.02	0.01	650.00
GOBP_REGULATION_OF_CHEMOTAXIS	28.00	-0.47	-1.90	0.00	0.02	0.02	357.00
GOBP_REGULATION_OF_VASCULAR_ASSOCIATED_SMOOTH_MUSCLE_CELL_PROLIFERATION	12.00	-0.61	-1.90	0.00	0.03	0.02	275.00
GOBP_CAMERA_TYPE_EYE_DEVELOPMENT	43.00	-0.41	-1.90	0.00	0.02	0.01	351.00
GOBP_EXOCYTOSIS	102.00	-0.34	-1.90	0.00	0.01	0.00	447.00
GOBP_REGULATION_OF_ALPHA_BETA_T_CELL_ACTIVATION	15.00	-0.56	-1.90	0.01	0.04	0.03	568.00
GOBP_ALPHA_BETA_T_CELL_ACTIVATION	23.00	-0.49	-1.90	0.00	0.03	0.02	568.00
GOBP_SPROUTING_ANGIOGENESIS	18.00	-0.53	-1.89	0.00	0.03	0.02	411.00
GOBP_REGULATION_OF_CELL_SUBSTRATE_ADHESION	25.00	-0.48	-1.89	0.00	0.02	0.02	550.00
GOBP_EMBRYO_DEVELOPMENT	103.00	-0.34	-1.88	0.00	0.01	0.01	316.00
GOBP_RESPONSE_TO_ACTIVITY	10.00	-0.64	-1.88	0.00	0.03	0.02	292.00
GOBP_EPIDERMIS_DEVELOPMENT	24.00	-0.48	-1.88	0.01	0.04	0.03	399.00
GOBP_EYE_MORPHOGENESIS	15.00	-0.55	-1.88	0.01	0.05	0.03	345.00
GOBP_TISSUE_REMODELING	23.00	-0.49	-1.87	0.00	0.03	0.02	612.00
GOBP_ENDOTHELIAL_CELL_APOPTOTIC_PROCESS	11.00	-0.62	-1.87	0.00	0.02	0.02	454.00
GOBP_GASTRULATION	18.00	-0.52	-1.87	0.00	0.03	0.02	537.00
GOBP_NEGATIVE_REGULATION_OF_SIGNALING	125.00	-0.32	-1.87	0.00	0.00	0.00	551.00
GOBP_POSITIVE_REGULATION_OF_MAPK_CASCADE	55.00	-0.38	-1.87	0.00	0.02	0.01	278.00
GOBP_POSITIVE_REGULATION_OF_LEUKOCYTE_CELL_CELL_ADHESION	33.00	-0.44	-1.86	0.00	0.02	0.02	597.00
GOBP_CELL_CELL_ADHESION	97.00	-0.34	-1.86	0.00	0.01	0.01	575.00
GOBP_ACTIN_FILAMENT_ORGANIZATION	41.00	-0.41	-1.86	0.01	0.04	0.03	429.00
GOBP_RESPONSE_TO_OXYGEN_CONTAINING_COMPOUND	154.00	-0.31	-1.86	0.00	0.01	0.00	542.00
GOBP_POSITIVE_REGULATION_OF_CELL_DEATH	68.00	-0.36	-1.86	0.00	0.02	0.01	547.00
GOBP_REGULATION_OF_IMMUNE_SYSTEM_PROCESS	140.00	-0.32	-1.86	0.00	0.00	0.00	653.00
GOBP_RESPONSE_TO_OXYGEN_LEVELS	47.00	-0.40	-1.86	0.00	0.02	0.01	316.00
GOBP_PLATELET_DEGRANULATION	20.00	-0.50	-1.86	0.01	0.04	0.03	498.00
GOBP_REGULATION_OF_LEUKOCYTE_MIGRATION	26.00	-0.47	-1.86	0.01	0.04	0.03	392.00
GOBP_TEMPERATURE_HOMEOSTASIS	18.00	-0.52	-1.86	0.01	0.03	0.02	444.00
GOBP_REGULATION_OF_CYTOSOLIC_CALCIUM_ION_CONCENTRATION	35.00	-0.43	-1.85	0.00	0.03	0.02	426.00

GOBP_ENTRY_INTO_HOST	18.00	-0.52	-1.85	0.01	0.04	0.03	408.00
GOBP_MUSCLE_CELL_MIGRATION	15.00	-0.55	-1.85	0.01	0.06	0.04	198.00
GOBP_INTEGRIN_MEDIATED_SIGNALING_PATHWAY	17.00	-0.52	-1.85	0.01	0.04	0.03	440.00
GOBP_REGULATION_OF_BLOOD_CIRCULATION	36.00	-0.42	-1.85	0.00	0.02	0.02	436.00
GOBP_HEART_PROCESS	36.00	-0.42	-1.84	0.00	0.02	0.02	436.00
GOBP_POSITIVE_REGULATION_OF_CELL_DIFFERENTIATION	84.00	-0.34	-1.84	0.00	0.02	0.01	443.00
GOBP_REGULATION_OF_CELL_CELL_ADHESION	53.00	-0.38	-1.84	0.00	0.02	0.02	575.00
GOBP_EMBRYONIC_ORGAN_DEVELOPMENT	46.00	-0.39	-1.84	0.00	0.03	0.02	504.00
GOBP_NEURAL_CREST_CELL_DIFFERENTIATION	10.00	-0.62	-1.84	0.01	0.04	0.03	378.00
GOBP_REGENERATION	15.00	-0.54	-1.83	0.01	0.06	0.04	504.00
GOBP_CARDIAC_CHAMBER_MORPHOGENESIS	19.00	-0.51	-1.83	0.01	0.04	0.03	351.00
GOBP_REGULATION_OF_BODY_FLUID_LEVELS	54.00	-0.38	-1.83	0.00	0.02	0.02	416.00
GOBP_APOPTOTIC_SIGNALING_PATHWAY	58.00	-0.37	-1.83	0.00	0.03	0.02	379.00
GOBP_NEGATIVE_REGULATION_OF_CYTOKINE_PRODUCTION	32.00	-0.43	-1.82	0.00	0.03	0.02	526.00
GOBP_LEUKOCYTE_CELL_CELL_ADHESION	47.00	-0.39	-1.82	0.00	0.02	0.02	597.00
GOBP_REGULATION_OF_INTRACELLULAR_SIGNAL_TRANSDUCTION	174.00	-0.30	-1.82	0.00	0.00	0.00	586.00
GOBP_ADENYLATE_CYCLASE_MODULATING_G_PROTEIN_COUPLED_RECEPTOR_SIGNALING_PATHWAY	22.00	-0.48	-1.82	0.01	0.04	0.03	416.00
GOBP_REACTIVE_OXYGEN_SPECIES_BIOSYNTHETIC_PROCESS	15.00	-0.54	-1.82	0.01	0.07	0.05	163.00
GOBP_TISSUE_HOMEOSTASIS	30.00	-0.44	-1.81	0.01	0.04	0.03	441.00
GOBP_REGULATION_OF_CELL_DIFFERENTIATION	152.00	-0.30	-1.81	0.00	0.01	0.00	504.00
GOBP_RECEPTOR_MEDIATED_ENDOCYTOSIS	28.00	-0.44	-1.80	0.01	0.04	0.03	509.00
GOBP_COLLAGEN_METABOLIC_PROCESS	12.00	-0.58	-1.80	0.01	0.05	0.04	517.00
GOBP_NEGATIVE_REGULATION_OF_CELL_ADHESION	32.00	-0.42	-1.80	0.00	0.03	0.02	575.00
GOBP_BIOLOGICAL_PROCESS_INVOLVED_IN_INTERACTION_WITH_HOST	22.00	-0.47	-1.80	0.01	0.05	0.03	408.00
GOBP_LYMPHOCYTE_MIGRATION	13.00	-0.55	-1.79	0.01	0.05	0.04	199.00
GOBP_SMOOTH_MUSCLE_CELL_MIGRATION	13.00	-0.55	-1.79	0.01	0.05	0.04	575.00
GOBP_REGULATION_OF_LEUKOCYTE_CHEMOTAXIS	13.00	-0.55	-1.79	0.01	0.05	0.04	178.00
GOBP_ADAPTIVE_THERMOGENESIS	14.00	-0.54	-1.79	0.01	0.05	0.04	402.00
GOBP_ARTERY_MORPHOGENESIS	11.00	-0.59	-1.79	0.01	0.04	0.03	507.00
GOBP_REGULATION_OF_RESPONSE_TO_WOUNDING	21.00	-0.48	-1.79	0.01	0.04	0.03	498.00
GOBP_MOVEMENT_IN_HOST_ENVIRONMENT	19.00	-0.49	-1.79	0.01	0.05	0.04	408.00
GOBP_RESPONSE_TO_GROWTH_FACTOR	87.00	-0.33	-1.79	0.00	0.01	0.01	354.00
GOBP_DIGESTION	10.00	-0.60	-1.78	0.01	0.06	0.04	260.00
GOBP_RESPONSE_TO_LIPID	89.00	-0.33	-1.78	0.00	0.01	0.01	619.00
GOBP_POSITIVE_REGULATION_OF_PHAGOCYTOSIS	11.00	-0.59	-1.78	0.01	0.04	0.03	296.00
GOBP_ORGANIC_ANION_TRANSPORT	31.00	-0.42	-1.77	0.01	0.05	0.04	511.00
GOBP_VASCULAR_TRANSPORT	11.00	-0.58	-1.77	0.01	0.05	0.03	304.00
GOBP_LEUKOCYTE_HOMEOSTASIS	10.00	-0.60	-1.76	0.01	0.06	0.04	378.00
GOBP_NEGATIVE_REGULATION_OF_HYDROLASE_ACTIVITY	40.00	-0.39	-1.76	0.01	0.05	0.04	340.00
GOBP_NEGATIVE_REGULATION_OF_PROTEOLYSIS	27.00	-0.44	-1.76	0.01	0.05	0.04	234.00
GOBP_POSITIVE_REGULATION_OF_LEUKOCYTE_MIGRATION	19.00	-0.49	-1.76	0.01	0.06	0.04	392.00
GOBP_HORMONE_TRANSPORT	24.00	-0.45	-1.76	0.02	0.08	0.06	436.00
GOBP_CAMERA_TYPE_EYE_MORPHOGENESIS	11.00	-0.58	-1.75	0.01	0.05	0.04	345.00
GOBP_REGULATION_OF_HEMOPOIESIS	39.00	-0.39	-1.75	0.01	0.04	0.03	574.00
GOBP_LYMPHOCYTE_APOPTOTIC_PROCESS	10.00	-0.59	-1.75	0.01	0.06	0.04	409.00
GOBP_T_CELL_APOPTOTIC_PROCESS	10.00	-0.59	-1.75	0.01	0.06	0.04	409.00

GOBP_CHEMICAL_HOMEOSTASIS	106.00	-0.31	-1.75	0.00	0.02	0.02	413.00
GOBP_POSITIVE_REGULATION_OF_PEPTIDE_SECRETION	11.00	-0.58	-1.75	0.01	0.05	0.04	292.00
GOBP_NEGATIVE_REGULATION_OF_TRANSPORT	45.00	-0.38	-1.75	0.01	0.06	0.04	186.00
GOBP_LEUKOCYTE_DIFFERENTIATION	59.00	-0.35	-1.75	0.01	0.04	0.03	612.00
GOBP_UROGENITAL_SYSTEM_DEVELOPMENT	43.00	-0.38	-1.74	0.01	0.04	0.03	252.00
GOBP_MONONUCLEAR_CELL_MIGRATION	24.00	-0.45	-1.74	0.02	0.09	0.06	392.00
GOBP_INNATE_IMMUNE_RESPONSE	88.00	-0.32	-1.74	0.00	0.02	0.01	513.00
GOBP_RENAL_SYSTEM_PROCESS	15.00	-0.51	-1.74	0.02	0.09	0.07	209.00
GOBP_BRANCHING_MORPHOGENESIS_OF_AN_EPITHELIAL_TUBE	16.00	-0.51	-1.74	0.02	0.07	0.05	525.00
GOBP_IMMUNE_SYSTEM_DEVELOPMENT	103.00	-0.31	-1.74	0.00	0.03	0.02	583.00
GOBP_B_CELL_DIFFERENTIATION	16.00	-0.50	-1.73	0.02	0.07	0.05	601.00
GOBP_DETOXIFICATION	10.00	-0.58	-1.73	0.01	0.07	0.05	189.00
GOBP_G_PROTEIN_COUPLED_RECEPTOR_SIGNALING_PATHWAY	82.00	-0.32	-1.73	0.00	0.03	0.02	329.00
GOBP_POSITIVE_REGULATION_OF_ENDOTHELIAL_CELL_MIGRATION	16.00	-0.50	-1.73	0.02	0.08	0.05	526.00
GOBP_EXTRINSIC_APOPTOTIC_SIGNALING_PATHWAY_VIA_DEATH_DOMAIN_RECEPTORS	13.00	-0.53	-1.73	0.01	0.06	0.05	500.00
GOBP_ENDOCYTOSIS	56.00	-0.35	-1.72	0.01	0.06	0.04	542.00
GOBP_RESPONSE_TO_ESTRADIOL	12.00	-0.55	-1.72	0.02	0.08	0.06	246.00
GOBP_GROWTH	84.00	-0.32	-1.72	0.01	0.04	0.03	355.00
GOBP_POSITIVE_REGULATION_OF_LYMPHOCYTE_DIFFERENTIATION	14.00	-0.52	-1.72	0.02	0.07	0.05	568.00
GOBP_POSITIVE_REGULATION_OF_PHOSPHATIDYLINOSITOL_3_KINASE_SIGNALING	15.00	-0.51	-1.72	0.03	0.10	0.07	507.00
GOBP_ADENYLATE_CYCLASE_INHIBITING_G_PROTEIN_COUPLED_RECEPTOR_SIGNALING_PATHWAY	11.00	-0.57	-1.72	0.01	0.06	0.04	416.00
GOBP_REGULATION_OF_MUSCLE_SYSTEM_PROCESS	25.00	-0.44	-1.72	0.02	0.07	0.05	621.00
GOBP_POSITIVE_REGULATION_OF_ALPHA_BETA_T_CELL_ACTIVATION	12.00	-0.55	-1.72	0.02	0.08	0.06	568.00
GOBP_OUTFLOW_TRACT_MORPHOGENESIS	10.00	-0.58	-1.72	0.02	0.07	0.05	504.00
GOBP_RESPONSE_TO_ENDOGENOUS_STIMULUS	145.00	-0.29	-1.72	0.00	0.02	0.01	422.00
GOBP_CELL_MORPHOGENESIS	104.00	-0.30	-1.71	0.00	0.02	0.01	467.00
GOBP_FOCAL_ADHESION_ASSEMBLY	14.00	-0.52	-1.71	0.02	0.07	0.05	466.00
GOBP_ARTERY_DEVELOPMENT	12.00	-0.55	-1.71	0.02	0.08	0.06	507.00
GOBP_SIGNAL_TRANSDUCTION_IN_ABSENCE_OF_LIGAND	11.00	-0.56	-1.71	0.01	0.06	0.05	286.00
GOBP_B_CELL_ACTIVATION	24.00	-0.44	-1.71	0.03	0.10	0.07	454.00
GOBP_POSITIVE_REGULATION_OF_CELL_DIVISION	11.00	-0.56	-1.71	0.01	0.06	0.05	275.00
GOBP_ACTIN_MEDIATED_CELL_CONTRACTION	15.00	-0.50	-1.71	0.03	0.11	0.08	416.00
GOBP_RESPONSE_TO_TUMOR_NECROSIS_FACTOR	40.00	-0.38	-1.70	0.02	0.07	0.05	632.00
GOBP_NEGATIVE_REGULATION_OF_IMMUNE_SYSTEM_PROCESS	41.00	-0.37	-1.70	0.02	0.08	0.06	275.00
GOBP_TISSUE_MORPHOGENESIS	62.00	-0.33	-1.70	0.02	0.07	0.05	416.00
GOBP_REGULATION_OF_T_CELL_ACTIVATION	40.00	-0.38	-1.70	0.02	0.07	0.05	570.00
GOBP_REGULATION_OF_MUSCLE_CONTRACTION	22.00	-0.44	-1.70	0.02	0.07	0.05	621.00
GOBP_CARDIAC_VENTRICLE_MORPHOGENESIS	12.00	-0.54	-1.70	0.02	0.09	0.07	334.00
GOBP_NEURON_APOPTOTIC_PROCESS	26.00	-0.43	-1.70	0.02	0.07	0.05	379.00
GOBP_PEPTIDE_HORMONE_SECRETION	19.00	-0.47	-1.69	0.02	0.07	0.05	436.00
GOBP_SUBSTRATE_ADHESION_DEPENDENT_CELL_SPREADING	15.00	-0.50	-1.69	0.03	0.12	0.09	416.00
GOBP_CD4_POSITIVE_ALPHA_BETA_T_CELL_ACTIVATION	15.00	-0.50	-1.69	0.03	0.12	0.09	526.00
GOBP_HEPATICOBILIARY_SYSTEM_DEVELOPMENT	12.00	-0.54	-1.68	0.03	0.10	0.07	437.00

GOBP_REGULATION_OF_WOUND_HEALING	18.00	-0.47	-1.68	0.03	0.10	0.07	388.00
GOBP_REGULATION_OF_CELL_MATRIX_ADHESION	14.00	-0.51	-1.68	0.02	0.08	0.06	478.00
GOBP_DEVELOPMENTAL_GROWTH	58.00	-0.34	-1.68	0.01	0.07	0.05	351.00
GOBP_METAL_ION_HOMEOSTASIS	67.00	-0.33	-1.68	0.01	0.05	0.03	456.00
GOBP_PROTEIN_PROCESSING	25.00	-0.43	-1.68	0.02	0.08	0.06	355.00
GOBP_GLAND_MORPHOGENESIS	14.00	-0.51	-1.67	0.02	0.08	0.06	300.00
GOBP_REGULATION_OF_REACTIVE_OXYGEN_SPECIES_BIOSYNTHETIC_PROCESS	13.00	-0.51	-1.67	0.02	0.08	0.06	104.00
GOBP_RESPONSE_TO_MECHANICAL_STIMULUS	22.00	-0.44	-1.67	0.04	0.13	0.10	224.00
GOBP_POSITIVE_REGULATION_OF_COLD_INDUCED_THERMOGENESIS	11.00	-0.55	-1.67	0.02	0.08	0.06	402.00
GOBP_NEGATIVE_REGULATION_OF_LOCOMOTION	33.00	-0.39	-1.66	0.02	0.08	0.06	479.00
GOBP_CELL_JUNCTION_ORGANIZATION	69.00	-0.32	-1.66	0.01	0.07	0.05	466.00
GOBP_HEART_MORPHOGENESIS	32.00	-0.39	-1.66	0.02	0.07	0.05	441.00
GOBP_NEGATIVE_REGULATION_OF_MOLECULAR_FUNCTION	98.00	-0.30	-1.66	0.01	0.05	0.03	296.00
GOBP_POSITIVE_REGULATION_OF_ENDOCYTOSIS	12.00	-0.53	-1.66	0.03	0.11	0.08	396.00
GOBP_CELLULAR_RESPONSE_TO_OXYGEN_CONTAINING_COMPOUND	100.00	-0.30	-1.66	0.01	0.04	0.03	498.00
GOBP_MUSCLE_STRUCTURE_DEVELOPMENT	51.00	-0.35	-1.66	0.02	0.07	0.05	596.00
GOBP_HEART_DEVELOPMENT	63.00	-0.33	-1.66	0.01	0.07	0.05	534.00
GOBP_REGULATION_OF_PROTEIN_PHOSPHORYLATION	110.00	-0.29	-1.65	0.01	0.06	0.04	540.00
GOBP_REGULATION_OF_LIPID_METABOLIC_PROCESS	36.00	-0.38	-1.65	0.02	0.08	0.06	213.00
GOBP_POSITIVE_REGULATION_OF_REACTIVE_OXYGEN_SPECIES_METABOLIC_PROCESS	18.00	-0.46	-1.65	0.04	0.14	0.10	526.00
GOBP_MORPHOGENESIS_OF_AN_EPITHELIUM	51.00	-0.35	-1.65	0.02	0.07	0.05	416.00
GOBP_APPENDAGE_MORPHOGENESIS	11.00	-0.54	-1.65	0.02	0.09	0.06	300.00
GOBP_RESPONSE_TO_KETONE	18.00	-0.46	-1.65	0.04	0.14	0.10	400.00
GOBP_REGULATION_OF_SMOOTH_MUSCLE_CONTRACTION	10.00	-0.56	-1.65	0.04	0.14	0.10	471.00
GOBP_REGULATION_OF_ALPHA_BETA_T_CELL_DIFFERENTIATION	11.00	-0.54	-1.65	0.02	0.09	0.07	568.00
GOBP_REGULATION_OF_PHAGOCYTOSIS	13.00	-0.51	-1.64	0.02	0.09	0.07	435.00
GOBP_NEGATIVE_REGULATION_OF_SMOOTH_MUSCLE_CELL_PROLIFERATION	12.00	-0.52	-1.64	0.03	0.12	0.09	275.00
GOBP_RESPONSE_TO_FIBROBLAST_GROWTH_FACTOR	14.00	-0.50	-1.64	0.02	0.09	0.07	174.00
GOBP_ION_HOMEOSTASIS	79.00	-0.31	-1.64	0.01	0.06	0.04	426.00
GOBP_CELL_CELL_SIGNALING	144.00	-0.28	-1.64	0.00	0.03	0.02	422.00
GOBP_REGULATION_OF_LYMPHOCYTE_DIFFERENTIATION	22.00	-0.43	-1.64	0.05	0.15	0.11	574.00
GOBP_T_CELL_ACTIVATION	62.00	-0.32	-1.64	0.03	0.10	0.07	574.00
GOBP_ANATOMICAL_STRUCTURE_HOMEOSTASIS	47.00	-0.35	-1.64	0.01	0.06	0.04	405.00
GOBP_ENDOTHELIUM_DEVELOPMENT	27.00	-0.41	-1.64	0.02	0.10	0.07	593.00
GOBP_TUMOR_NECROSIS_FACTOR_SUPERFAMILY_CYTOKINE_PRODUCTION	19.00	-0.45	-1.64	0.04	0.14	0.10	281.00
GOBP_RESPONSE_TO_ORGANIC_CYCLIC_COMPOUND	87.00	-0.30	-1.64	0.01	0.04	0.03	507.00
GOBP_REGULATION_OF_PEPTIDE_HORMONE_SECRETION	18.00	-0.46	-1.64	0.04	0.14	0.10	436.00
GOBP_NEGATIVE_REGULATION_OF_PROTEIN_METABOLIC_PROCESS	75.00	-0.31	-1.64	0.01	0.07	0.05	537.00
GOBP_LYMPHOCYTE_ACTIVATION	80.00	-0.31	-1.64	0.02	0.07	0.05	607.00
GOBP_POSITIVE_REGULATION_OF_CELLULAR_COMPONENT_ORGANIZATION	103.00	-0.29	-1.64	0.01	0.07	0.05	416.00
GOBP_REPRODUCTIVE_SYSTEM_DEVELOPMENT	50.00	-0.35	-1.64	0.01	0.06	0.04	550.00

GOBP_RECEPTOR_SIGNALING_PATHWAY_VIA_STAT	16.00	-0.48	-1.63	0.04	0.14	0.10	542.00
GOBP_CELL_RECOGNITION	21.00	-0.44	-1.63	0.05	0.15	0.11	226.00
GOBP_REGULATION_OF_CELL_ACTIVATION	67.00	-0.32	-1.63	0.01	0.06	0.04	575.00
GOBP_ODONTOGENESIS	14.00	-0.49	-1.63	0.03	0.10	0.07	335.00
GOBP_REGULATION_OF_RESPONSE_TO_EXTERNAL_STIMULUS	114.00	-0.29	-1.63	0.01	0.06	0.04	513.00
GOBP_HOMEOSTASIS_OF_NUMBER_OF_CELLS	27.00	-0.40	-1.63	0.03	0.10	0.07	397.00
GOBP_RESPONSE_TO_MONOSACCHARIDE	20.00	-0.44	-1.63	0.04	0.14	0.10	400.00
GOBP_REGULATION_OF_PHOSPHATIDYLINOSITOL_3_KINASE_SIGNALING	17.00	-0.46	-1.62	0.05	0.16	0.12	278.00
GOBP_CELL_MIGRATION_INVOLVED_IN_SPROUTING_ANGIOGENESIS	10.00	-0.55	-1.62	0.05	0.15	0.11	351.00
GOBP_EPITHELIAL_TUBE_MORPHOGENESIS	31.00	-0.38	-1.62	0.03	0.11	0.08	355.00
GOBP_REGULATION_OF_HORMONE_SECRETION	20.00	-0.44	-1.62	0.04	0.14	0.10	436.00
GOBP_POSITIVE_REGULATION_OF_NF_KAPPAB_TRANSCRIPTION_FACTOR_ACTIVITY	13.00	-0.50	-1.62	0.04	0.14	0.10	688.00
GOBP_REGULATION_OF_ACTIN_FILAMENT_ORGANIZATION	28.00	-0.40	-1.62	0.02	0.09	0.07	416.00
GOBP_RETINA_DEVELOPMENT_IN_CAMERA_TYPE_EYE	25.00	-0.41	-1.62	0.04	0.13	0.09	351.00
GOBP_CELLULAR_RESPONSE_TO_LIPID	54.00	-0.33	-1.61	0.02	0.08	0.05	706.00
GOBP_ADAPTIVE_IMMUNE_RESPONSE_BASED_ON_SOMATIC_RECOMBINATION_OF_IMMUNE_RECEPTORS_BUILT_FROM_IMMUNOGLOBULIN_SUPERFAMILY_DOMAINS	37.00	-0.36	-1.61	0.04	0.13	0.09	547.00
GOBP_REGULATION_OF_SUPRAMOLECULAR_FIBER_ORGANIZATION	33.00	-0.38	-1.61	0.02	0.09	0.07	416.00
GOBP_EAR_DEVELOPMENT	20.00	-0.44	-1.61	0.05	0.15	0.11	399.00
GOBP_NEGATIVE_REGULATION_OF_CELL_DIFFERENTIATION	46.00	-0.34	-1.61	0.02	0.09	0.07	547.00
GOBP_MORPHOGENESIS_OF_A_BRANCHING_STRUCTURE	22.00	-0.42	-1.61	0.05	0.16	0.11	355.00
GOBP_MONONUCLEAR_CELL_DIFFERENTIATION	47.00	-0.34	-1.61	0.01	0.07	0.05	574.00
GOBP_REGULATION_OF_HEART_RATE	12.00	-0.51	-1.60	0.04	0.14	0.10	426.00
GOBP_CYTOKINE_PRODUCTION_INVOLVED_IN_IMMUNE_RESPONSE	12.00	-0.51	-1.60	0.04	0.14	0.10	547.00
GOBP_COAGULATION	41.00	-0.35	-1.60	0.03	0.12	0.09	582.00
GOBP_REGULATION_OF_T_CELL_DIFFERENTIATION	18.00	-0.45	-1.60	0.05	0.16	0.12	570.00
GOBP_MORPHOGENESIS_OF_EMBRYONIC_EPITHELIUM	11.00	-0.53	-1.60	0.05	0.15	0.11	292.00
GOBP_RESPONSE_TO_INORGANIC_SUBSTANCE	56.00	-0.32	-1.60	0.03	0.11	0.08	335.00
GOBP_GLAND_DEVELOPMENT	43.00	-0.35	-1.60	0.03	0.11	0.08	305.00
GOBP_NEGATIVE_REGULATION_OF_CELL_ACTIVATION	22.00	-0.42	-1.60	0.05	0.16	0.11	575.00
GOBP_REGULATION_OF_HEART_CONTRACTION	32.00	-0.38	-1.60	0.04	0.12	0.09	436.00
GOBP_REGULATION_OF_HORMONE_LEVELS	33.00	-0.37	-1.60	0.03	0.10	0.07	436.00
GOBP_REGULATION_OF_HYDROLASE_ACTIVITY	119.00	-0.28	-1.60	0.01	0.06	0.04	632.00
GOBP_REGULATION_OF_CELLULAR_RESPONSE_TO_GROWTH_FACTOR_STIMULUS	29.00	-0.39	-1.60	0.04	0.13	0.09	550.00
GOBP_REACTIVE_OXYGEN_SPECIES_METABOLIC_PROCESS	32.00	-0.38	-1.59	0.04	0.13	0.09	526.00
GOBP_POSITIVE_REGULATION_OF_ALPHA_BETA_T_CELL_DIFFERENTIATION	10.00	-0.54	-1.59	0.06	0.16	0.12	568.00
GOBP_CELL_JUNCTION_ASSEMBLY	43.00	-0.35	-1.59	0.04	0.13	0.09	466.00
GOBP_LEUKOCYTE_PROLIFERATION	36.00	-0.36	-1.59	0.04	0.12	0.09	570.00
GOBP_REGULATION_OF_REACTIVE_OXYGEN_SPECIES_METABOLIC_PROCESS	29.00	-0.39	-1.59	0.04	0.14	0.10	526.00
GOBP_REGULATION_OF_MAPK_CASCADE	69.00	-0.30	-1.58	0.03	0.10	0.07	278.00

GOBP_INSULIN_SECRETION	16.00	-0.46	-1.58	0.05	0.16	0.12	436.00
GOBP_POSITIVE_REGULATION_OF_HEMOPOIESIS	20.00	-0.43	-1.58	0.06	0.18	0.13	612.00
GOBP_CARBOHYDRATE_HOMEOSTASIS	16.00	-0.46	-1.58	0.05	0.16	0.12	344.00
GOBP_POSITIVE_REGULATION_OF_NEURON_DEATH	10.00	-0.53	-1.58	0.06	0.17	0.12	494.00
GOBP_INTERFERON_GAMMA_MEDIATED_SIGNALING_PATHWAY	16.00	-0.46	-1.58	0.05	0.16	0.12	650.00
GOBP_NEGATIVE_REGULATION_OF_CATALYTIC_ACTIVITY	69.00	-0.30	-1.58	0.03	0.11	0.08	296.00
GOBP_PROTEIN_MATURATION	28.00	-0.39	-1.57	0.05	0.15	0.11	313.00
GOBP_ALPHA_BETA_T_CELL_DIFFERENTIATION	17.00	-0.45	-1.57	0.06	0.18	0.13	568.00
GOBP_MESENCHYMAL_CELL_DIFFERENTIATION	24.00	-0.40	-1.57	0.05	0.16	0.12	416.00
GOBP_MODULATION_OF_PROCESS_OF_OTHER_ORGANISM	13.00	-0.48	-1.57	0.05	0.16	0.12	422.00
GOBP_NEGATIVE_REGULATION_OF_NEURON_APOPTOTIC_PROCESS	14.00	-0.48	-1.57	0.06	0.17	0.12	292.00
GOBP_STRIATED_MUSCLE_CONTRACTION	23.00	-0.41	-1.57	0.06	0.18	0.13	590.00
GOBP_NEGATIVE_REGULATION_OF_TRANSMEMBRANE_TRANSPORT	13.00	-0.48	-1.57	0.05	0.16	0.12	186.00
GOBP_RESPONSE_TO_NUTRIENT	26.00	-0.39	-1.56	0.05	0.16	0.12	155.00
GOBP_REGULATION_OF_PRODUCTION_OF_MOLECULAR_MEDIATOR_OF_IMMUNE_RESPONSE	14.00	-0.47	-1.56	0.06	0.17	0.12	547.00
GOBP_POSITIVE_REGULATION_OF_CYTOSKELETON_ORGANIZATION	23.00	-0.40	-1.56	0.07	0.18	0.13	416.00
GOBP_CARDIAC_CHAMBER_DEVELOPMENT	25.00	-0.40	-1.56	0.05	0.16	0.12	351.00
GOBP_ORGANIC_HYDROXY_COMPOUND_TRANSPORT	23.00	-0.40	-1.56	0.07	0.18	0.13	203.00
GOBP_ADAPTIVE_IMMUNE_RESPONSE	52.00	-0.33	-1.56	0.04	0.13	0.09	526.00
GOBP_GLOMERULUS_DEVELOPMENT	15.00	-0.46	-1.56	0.06	0.18	0.13	540.00
GOBP_CELLULAR_HOMEOSTASIS	87.00	-0.29	-1.56	0.01	0.06	0.04	426.00
GOBP_RESPONSE_TO_CARBOHYDRATE	21.00	-0.42	-1.55	0.06	0.18	0.13	400.00
GOBP_CELL_MORPHOGENESIS_INVOLVED_IN_DIFFERENTIATION	67.00	-0.30	-1.55	0.02	0.08	0.06	416.00
GOBP_HEART_VALVE_DEVELOPMENT	11.00	-0.51	-1.55	0.05	0.16	0.12	525.00
GOBP_REGULATION_OF_PROTEOLYSIS	66.00	-0.30	-1.55	0.02	0.09	0.06	424.00
GOBP_REGULATION_OF_LEUKOCYTE_DIFFERENTIATION	30.00	-0.37	-1.55	0.05	0.16	0.12	664.00
GOBP_POSITIVE_REGULATION_OF_ERK1_AND_ERK2_CASCADE	19.00	-0.43	-1.55	0.07	0.19	0.14	664.00
GOBP_POSITIVE_REGULATION_OF_MAP_KINASE_ACTIVITY	30.00	-0.37	-1.54	0.05	0.16	0.12	550.00
GOBP_RESPONSE_TO_HYDROGEN_PEROXIDE	13.00	-0.48	-1.54	0.06	0.17	0.12	272.00
GOBP_MYOTUBE_DIFFERENTIATION	11.00	-0.51	-1.54	0.06	0.17	0.12	596.00
GOBP_PRODUCTION_OF_MOLECULAR_MEDIATOR_OF_IMMUNE_RESPONSE	18.00	-0.43	-1.54	0.07	0.19	0.14	547.00
GOBP_NEGATIVE_REGULATION_OF_LYMPHOCYTE_ACTIVATION	14.00	-0.47	-1.54	0.07	0.19	0.14	547.00
GOBP_BONE_MORPHOGENESIS	13.00	-0.47	-1.54	0.06	0.18	0.13	533.00
GOBP_CELLULAR_GLUCOSE_HOMEOSTASIS	12.00	-0.49	-1.54	0.06	0.17	0.12	292.00
GOBP_AMINOGLYCAN_METABOLIC_PROCESS	21.00	-0.41	-1.54	0.07	0.20	0.14	192.00
GOBP_POSITIVE_REGULATION_OF_PROTEIN_CONTAINING_COMPLEX_ASSEMBLY	21.00	-0.41	-1.54	0.07	0.20	0.14	296.00
GOBP_RESPONSE_TO_TOXIC_SUBSTANCE	22.00	-0.40	-1.53	0.08	0.20	0.14	304.00
GOBP_CARDIAC_MUSCLE_CONTRACTION	19.00	-0.42	-1.53	0.07	0.20	0.14	426.00
GOBP_NEGATIVE_REGULATION_OF_LIPID_METABOLIC_PROCESS	11.00	-0.51	-1.53	0.06	0.18	0.13	614.00
GOBP_ADENYLATE_CYCLASE_ACTIVATING_G_PROTEIN_COUPLED_RECEPTOR_SIGNALING_PATHWAY	11.00	-0.50	-1.53	0.06	0.18	0.13	310.00

GOBP_POSITIVE_REGULATION_OF_PHOSPHORUS_METABOLIC_PROCESS	94.00	-0.28	-1.53	0.02	0.08	0.06	525.00
GOBP_EPIDERMAL_GROWTH_FACTOR_RECEPTOR_SIGNALING_PATHWAY	13.00	-0.47	-1.53	0.06	0.18	0.13	337.00
GOBP_POSITIVE_REGULATION_OF_RESPONSE_TO_WOUNDING	10.00	-0.52	-1.53	0.08	0.20	0.14	489.00
GOBP_REGULATION_OF_IMMUNE_RESPONSE	86.00	-0.28	-1.53	0.02	0.09	0.07	531.00
GOBP_APICAL_JUNCTION_ASSEMBLY	11.00	-0.50	-1.52	0.07	0.19	0.14	441.00
GOBP_POSITIVE_REGULATION_OF_RESPONSE_TO_EXTERNAL_STIMULUS	63.00	-0.30	-1.52	0.04	0.13	0.09	617.00
GOBP_EPIDERMAL_CELL_DIFFERENTIATION	17.00	-0.43	-1.52	0.08	0.20	0.14	399.00
GOBP_PHOSPHATIDYLINOSITOL_3_KINASE_SIGNALING	19.00	-0.42	-1.52	0.07	0.20	0.14	278.00
GOBP_RESPONSE_TO_CORTICOSTEROID	13.00	-0.47	-1.52	0.07	0.19	0.14	228.00
GOBP_REGULATION_OF_ERBB_SIGNALING_PATHWAY	11.00	-0.50	-1.52	0.07	0.19	0.14	337.00
GOBP_MAPK_CASCADE	85.00	-0.28	-1.52	0.03	0.12	0.09	278.00
GOBP_NEGATIVE_REGULATION_OF_CELL_GROWTH	13.00	-0.47	-1.52	0.07	0.20	0.14	209.00
GOBP_DIVALENT_INORGANIC_CATION_HOMEOSTASIS	51.00	-0.32	-1.52	0.04	0.14	0.10	456.00
GOBP_CELLULAR_ION_HOMEOSTASIS	68.00	-0.29	-1.51	0.04	0.13	0.09	456.00
GOBP_REGULATION_OF_ACTIN_FILAMENT_BASED_PROCESS	44.00	-0.33	-1.51	0.05	0.16	0.11	541.00
GOBP_EMBRYONIC_APPENDAGE_MORPHOGENESIS	10.00	-0.51	-1.51	0.08	0.20	0.15	300.00
GOBP_POSITIVE_REGULATION_OF_CELL_ACTIVATION	44.00	-0.33	-1.51	0.05	0.16	0.12	570.00
GOBP_HEMATOPOIETIC_STEM_CELL_DIFFERENTIATION	11.00	-0.50	-1.51	0.07	0.20	0.14	192.00
GOBP_POSITIVE_REGULATION_OF_LEUKOCYTE_CHEMOTAXIS	10.00	-0.51	-1.51	0.08	0.21	0.15	357.00
GOBP_NEURON_DEATH	38.00	-0.34	-1.51	0.05	0.16	0.11	379.00
GOBP_CARDIAC_MUSCLE_CELL_ACTION_POTENTIAL_INVOLVED_IN_CONTRACTION	10.00	-0.51	-1.51	0.08	0.21	0.15	416.00
GOBP_CARDIAC_MUSCLE_CELL_CONTRACTION	10.00	-0.51	-1.51	0.08	0.21	0.15	416.00
GOBP_RESPONSE_TO_TYPE_I_INTERFERON	14.00	-0.46	-1.51	0.07	0.20	0.14	578.00
GOBP_MAINTENANCE_OF_LOCATION_IN_CELL	19.00	-0.42	-1.51	0.08	0.21	0.15	611.00
GOBP_APPENDAGE_DEVELOPMENT	15.00	-0.44	-1.50	0.08	0.20	0.14	300.00
GOBP_PALLIUM_DEVELOPMENT	11.00	-0.49	-1.50	0.08	0.21	0.15	432.00
GOBP_RESPONSE_TO_VITAMIN	16.00	-0.44	-1.50	0.08	0.21	0.15	226.00
GOBP_T_CELL_PROLIFERATION	25.00	-0.38	-1.50	0.08	0.20	0.14	713.00
GOBP_REGULATION_OF_TRANSMEMBRANE_TRANSPORT	47.00	-0.32	-1.50	0.05	0.16	0.11	438.00
GOBP_RESPONSE_TO_OXIDATIVE_STRESS	39.00	-0.34	-1.50	0.05	0.16	0.12	355.00
GOBP_MYELOID_CELL_DIFFERENTIATION	40.00	-0.33	-1.49	0.05	0.16	0.11	612.00
GOBP_ORGAN_GROWTH	17.00	-0.42	-1.49	0.09	0.22	0.16	593.00
GOBP_RESPONSE_TO_VIRUS	31.00	-0.35	-1.49	0.07	0.19	0.14	526.00
GOBP_STEM_CELL_DIFFERENTIATION	28.00	-0.37	-1.49	0.08	0.20	0.14	378.00
GOBP_PROTEOLYSIS	149.00	-0.25	-1.48	0.03	0.10	0.07	447.00
GOBP_CELLULAR_RESPONSE_TO_EXTERNAL_STIMULUS	37.00	-0.33	-1.48	0.07	0.19	0.13	256.00
GOBP_RESPONSE_TO_TRANSFORMING_GROWTH_FACTOR_BETA	35.00	-0.34	-1.48	0.06	0.16	0.12	287.00
GOBP_REGULATION_OF_CELL_DIVISION	16.00	-0.43	-1.48	0.09	0.22	0.16	178.00
GOBP_REGIONALIZATION	25.00	-0.38	-1.48	0.09	0.22	0.15	28.00
GOBP_REGULATION_OF_ION_TRANSPORT	115.00	-0.26	-1.48	0.03	0.12	0.09	438.00
GOBP_POSITIVE_REGULATION_OF_PROTEIN_PHOSPHORYLATION	84.00	-0.27	-1.48	0.04	0.14	0.10	508.00

GOBP_REGULATION_OF_MAP_KINASE_ACTIVITY	34.00	-0.34	-1.48	0.06	0.16	0.12	550.00
GOBP_REGULATION_OF_TRANSPORT	159.00	-0.24	-1.47	0.03	0.10	0.07	440.00
GOBP_REGULATION_OF_PROTEIN_POLYMERIZATION	18.00	-0.41	-1.47	0.09	0.22	0.16	406.00
GOBP_RESPONSE_TO_REACTIVE_OXYGEN_SPECIES	18.00	-0.41	-1.47	0.09	0.22	0.16	292.00
GOBP_RESPONSE_TO_HORMONE	74.00	-0.28	-1.47	0.05	0.15	0.11	422.00
GOBP_CELL_FATE_COMMITMENT	20.00	-0.40	-1.47	0.10	0.23	0.17	357.00
GOBP_NEGATIVE_REGULATION_OF_NEURON_DEATH	25.00	-0.37	-1.47	0.09	0.22	0.16	320.00
GOBP_NEGATIVE_REGULATION_OF_EPITHELIAL_CELL_MIGRATION	12.00	-0.47	-1.46	0.09	0.21	0.15	343.00
GOBP_REGULATION_OF_STRIATED_MUSCLE_CONTRACTION	10.00	-0.49	-1.46	0.10	0.23	0.17	590.00
GOBP_MYELOID_LEUKOCYTE_DIFFERENTIATION	23.00	-0.38	-1.46	0.09	0.22	0.16	664.00
GOBP_PLACENTA_DEVELOPMENT	16.00	-0.43	-1.46	0.09	0.22	0.16	574.00
GOBP_MESENCHYME_DEVELOPMENT	29.00	-0.36	-1.46	0.09	0.22	0.16	507.00
GOBP_POSITIVE_REGULATION_OF_GROWTH	23.00	-0.38	-1.46	0.09	0.22	0.16	504.00
GOBP_NEURON_PROJECTION_GUIDANCE	24.00	-0.38	-1.46	0.09	0.22	0.16	351.00
GOBP_PLATELET_ACTIVATION	22.00	-0.38	-1.46	0.10	0.24	0.17	576.00
GOBP_EMBRYONIC_PLACENTA_DEVELOPMENT	12.00	-0.47	-1.46	0.09	0.22	0.16	574.00
GOBP_CD4_POSITIVE_ALPHA_BETA_T_CELL_DIFFERENTIATION	12.00	-0.46	-1.46	0.09	0.22	0.16	526.00
GOBP_POSITIVE_REGULATION_OF_LIPID_LOCALIZATION	11.00	-0.48	-1.45	0.09	0.22	0.16	614.00
GOBP_ACTIN_CYTOSKELETON_REORGANIZATION	18.00	-0.41	-1.45	0.10	0.23	0.16	523.00
GOBP_POSITIVE_REGULATION_OF_PROTEIN_KINASE_B_SIGNALING	18.00	-0.41	-1.45	0.10	0.23	0.16	535.00
GOBP_REGULATION_OF_SECRETION	53.00	-0.30	-1.45	0.06	0.16	0.12	449.00
GOBP_SENSORY_PERCEPTION_OF_MECHANICAL_STIMULUS	16.00	-0.42	-1.45	0.10	0.24	0.17	224.00
GOBP_RHYTHMIC_PROCESS	33.00	-0.34	-1.45	0.07	0.20	0.14	287.00
GOBP_REGULATION_OF_LYMPHOCYTE_ACTIVATION	50.00	-0.31	-1.45	0.06	0.18	0.13	574.00
GOBP_REGULATION_OF_ANION_TRANSPORT	75.00	-0.27	-1.45	0.05	0.16	0.12	436.00
GOBP_MEMBRANE_INVAGINATION	10.00	-0.49	-1.44	0.11	0.25	0.18	644.00
GOBP_CALCIUM_ION_TRANSPORT_INTO_CYTOSOL	12.00	-0.46	-1.44	0.09	0.22	0.16	571.00
GOBP_POSITIVE_REGULATION_OF_ION_TRANSPORT	62.00	-0.28	-1.44	0.06	0.18	0.13	416.00
GOBP_CARBOXYLIC_ACID_TRANSPORT	25.00	-0.37	-1.44	0.09	0.22	0.16	511.00
GOBP_RESPONSE_TO_ETHANOL	19.00	-0.40	-1.44	0.10	0.24	0.17	477.00
GOBP_RESPONSE_TO_ABIOTIC_STIMULUS	118.00	-0.25	-1.44	0.05	0.16	0.11	475.00
GOBP_REGULATION_OF_PHOSPHORUS_METABOLIC_PROCESS	139.00	-0.25	-1.44	0.04	0.13	0.10	540.00
GOBP_MACROPHAGE_ACTIVATION	11.00	-0.47	-1.44	0.09	0.22	0.16	526.00
GOBP_GLIOGENESIS	25.00	-0.37	-1.44	0.10	0.23	0.16	376.00
GOBP_POSITIVE_REGULATION_OF_TRANSMEMBRANE_TRANSPORT	21.00	-0.38	-1.43	0.11	0.25	0.18	416.00
GOBP_REGULATION_OF_CYTOSKELETON_ORGANIZATION	46.00	-0.31	-1.43	0.08	0.21	0.15	541.00
GOBP_ERBB_SIGNALING_PATHWAY	15.00	-0.42	-1.43	0.10	0.23	0.17	337.00
GOBP_RESPONSE_TO_TEMPERATURE_STIMULUS	22.00	-0.37	-1.43	0.11	0.25	0.18	475.00
GOBP_NEGATIVE_REGULATION_OF_RESPONSE_TO_EXTERNAL_STIMULUS	52.00	-0.30	-1.43	0.07	0.19	0.14	513.00
GOBP_POSITIVE_REGULATION_OF_TRANSPORT	90.00	-0.26	-1.43	0.06	0.17	0.12	416.00
GOBP_CELLULAR_COMPONENT_DISASSEMBLY	47.00	-0.30	-1.43	0.07	0.20	0.14	406.00
GOBP_TRANSITION_METAL_ION_HOMEOSTASIS	14.00	-0.43	-1.43	0.11	0.25	0.18	316.00
GOBP_I_KAPPAB_KINASE_NF_KAPPAB_SIGNALING	38.00	-0.32	-1.43	0.08	0.21	0.15	706.00
GOBP_INOSITOL_LIPID_MEDIATED_SIGNALING	21.00	-0.38	-1.43	0.11	0.26	0.18	144.00
GOBP_MAINTENANCE_OF_LOCATION	34.00	-0.33	-1.42	0.08	0.20	0.14	664.00

GOBP_POSITIVE_REGULATION_OF_CELL_SUBSTRATE_ADHESION	14.00	-0.43	-1.42	0.11	0.25	0.18	540.00
GOBP_NEGATIVE_REGULATION_OF_ION_TRANSPORT	32.00	-0.34	-1.42	0.10	0.23	0.16	186.00
GOBP_POSITIVE_REGULATION_OF_DEVELOPMENTAL_GROWTH	15.00	-0.42	-1.42	0.10	0.24	0.17	504.00
GOBP_POSITIVE_REGULATION_OF_PROTEIN_SERINE_THREONINE_KINASE_ACTIVITY	37.00	-0.32	-1.42	0.08	0.21	0.15	550.00
GOBP_SIGNAL_RELEASE	40.00	-0.31	-1.42	0.07	0.19	0.14	163.00
GOBP_ENDOTHELIAL_CELL_DEVELOPMENT	15.00	-0.42	-1.42	0.11	0.25	0.18	593.00
GOBP_NEGATIVE_REGULATION_OF_LEUKOCYTE_CELL_CELL_ADHESION	14.00	-0.43	-1.42	0.11	0.25	0.18	713.00
GOBP_REGULATION_OF_CALCIUM_ION_TRANSMEMBRANE_TRANSPORT	13.00	-0.44	-1.42	0.11	0.24	0.17	553.00
GOBP_RESPONSE_TO_NITROGEN_COMPOUND	91.00	-0.26	-1.41	0.06	0.18	0.13	416.00
GOBP_RESPONSE_TO_METAL_ION	39.00	-0.32	-1.41	0.08	0.21	0.15	335.00
GOBP_CYTOSKELETON_ORGANIZATION	124.00	-0.25	-1.41	0.05	0.16	0.12	545.00
GOBP_INTERFERON_GAMMA_PRODUCTION	12.00	-0.45	-1.41	0.11	0.24	0.17	281.00
GOBP_REGULATION_OF_DNA_BINDING_TRANSCRIPTION_FACTOR_ACTIVITY	46.00	-0.30	-1.41	0.09	0.22	0.16	663.00
GOBP_CELL_AGING	13.00	-0.43	-1.41	0.11	0.25	0.18	147.00
GOBP_INTRINSIC_APOPTOTIC_SIGNALING_PATHWAY_IN_RESPONSE_TO_DNA_DAMAGE	12.00	-0.45	-1.41	0.11	0.25	0.18	551.00
GOBP_REGULATION_OF_PROTEIN_MODIFICATION_PROCESS	133.00	-0.24	-1.41	0.05	0.16	0.12	540.00
GOBP_CALCIUM_ION_TRANSPORT	35.00	-0.32	-1.40	0.09	0.22	0.16	571.00
GOBP_LYMPHOCYTE_MEDIATED_IMMUNITY	32.00	-0.33	-1.40	0.11	0.25	0.18	242.00
GOBP_MULTICELLULAR_ORGANISMAL_SIGNALING	21.00	-0.37	-1.39	0.13	0.27	0.20	534.00
GOBP_REGULATION_OF_PROTEIN_SERINE_THREONINE_KINASE_ACTIVITY	51.00	-0.29	-1.39	0.09	0.22	0.16	593.00
GOBP_REGULATION_OF_IMMUNE_EFFECTOR_PROCESS	42.00	-0.30	-1.39	0.09	0.22	0.16	547.00
GOBP_ACTIVATION_OF_MAPK_ACTIVITY	18.00	-0.39	-1.39	0.12	0.27	0.19	275.00
GOBP_INTERLEUKIN_6_PRODUCTION	15.00	-0.41	-1.39	0.13	0.28	0.20	526.00
GOBP_CELLULAR_RESPONSE_TO_REACTIVE_OXYGEN_SPECIES	12.00	-0.44	-1.39	0.12	0.26	0.19	136.00
GOBP_REGULATION_OF_RESPONSE_TO_STRESS	130.00	-0.24	-1.39	0.07	0.19	0.14	252.00
GOBP_MUSCLE_ORGAN_DEVELOPMENT	24.00	-0.36	-1.38	0.11	0.26	0.18	334.00
GOBP_POSITIVE_REGULATION_OF_PROTEIN_METABOLIC_PROCESS	147.00	-0.23	-1.38	0.06	0.18	0.13	508.00
GOBP_MYELOID_CELL_HOMEOSTASIS	16.00	-0.40	-1.38	0.13	0.28	0.20	601.00
GOBP_REGULATION_OF_PROTEIN_KINASE_ACTIVITY	74.00	-0.26	-1.38	0.07	0.20	0.14	560.00
GOBP_POSITIVE_REGULATION_OF_SMALL_MOLECULE_METABOLIC_PROCESS	10.00	-0.47	-1.38	0.13	0.28	0.20	400.00
GOBP_NEGATIVE_REGULATION_OF_GENE_EXPRESSION	90.00	-0.25	-1.38	0.07	0.20	0.14	702.00
GOBP_CYTOSOLIC_CALCIUM_ION_TRANSPORT	14.00	-0.42	-1.38	0.13	0.28	0.20	571.00
GOBP_POSITIVE_REGULATION_OF_DNA_BINDING_TRANSCRIPTION_FACTOR_ACTIVITY	21.00	-0.37	-1.38	0.13	0.27	0.20	749.00
GOBP_CARDIAC_VENTRICLE_DEVELOPMENT	19.00	-0.38	-1.37	0.13	0.27	0.20	334.00
GOBP_REGULATION_OF_LEUKOCYTE_PROLIFERATION	25.00	-0.35	-1.37	0.12	0.27	0.19	570.00
GOBP_POSITIVE_REGULATION_OF_IMMUNE_RESPONSE	63.00	-0.27	-1.37	0.08	0.21	0.15	547.00
GOBP_SEQUESTERING_OF_CALCIUM_ION	11.00	-0.45	-1.37	0.13	0.28	0.20	571.00
GOBP_POSITIVE_REGULATION_OF_INFLAMMATORY_RESPONSE	15.00	-0.40	-1.37	0.14	0.29	0.21	505.00
GOBP_REGULATION_OF_MONONUCLEAR_CELL_MIGRATION	12.00	-0.44	-1.36	0.13	0.28	0.20	174.00

GOBP_REGULATION_OF_PROTEIN_TYROSINE_KINASE_ACTIVITY	10.00	-0.46	-1.36	0.14	0.30	0.22	523.00
GOBP_POSITIVE_REGULATION_OF_TUMOR_NECROSIS_FACTOR_SUPERFAMILY_CYTOKINE_PRODUCTION	10.00	-0.46	-1.36	0.15	0.31	0.22	494.00
GOBP_POSITIVE_REGULATION_OF_ANION_TRANSPORT	38.00	-0.31	-1.36	0.11	0.25	0.18	526.00
GOBP_ACTION_POTENTIAL	16.00	-0.40	-1.36	0.14	0.29	0.21	212.00
GOBP_PHOSPHATIDYLINOSITOL_METABOLIC_PROCESS	16.00	-0.40	-1.36	0.14	0.30	0.21	549.00
GOBP_POSITIVE_REGULATION_OF_SYNAPTIC_TRANSMISSION	10.00	-0.46	-1.35	0.15	0.31	0.22	285.00
GOBP_BONE_DEVELOPMENT	20.00	-0.37	-1.35	0.14	0.30	0.21	678.00
GOBP_POSITIVE_REGULATION_OF_LIPID_METABOLIC_PROCESS	12.00	-0.43	-1.35	0.14	0.29	0.21	507.00
GOBP_REGULATION_OF_PROTEIN_CONTAINING_COMPLEX_ASSEMBLY	34.00	-0.31	-1.35	0.12	0.27	0.19	406.00
GOBP_ANTIGEN_RECEPTOR_MEDIATED_SIGNALING_PATHWAY	21.00	-0.36	-1.35	0.14	0.29	0.21	700.00
GOBP_SMALL_GTPASE_MEDIATED_SIGNAL_TRANSDUCTION	55.00	-0.28	-1.35	0.11	0.25	0.18	613.00
GOBP_RAS_PROTEIN_SIGNAL_TRANSDUCTION	32.00	-0.32	-1.35	0.14	0.30	0.21	613.00
GOBP_BIOLOGICAL_PROCESS_INVOLVED_IN_SYMBIOTIC_INTERACTION	87.00	-0.25	-1.35	0.10	0.23	0.17	475.00
GOBP_ANION_TRANSMEMBRANE_TRANSPORT	44.00	-0.29	-1.35	0.12	0.26	0.19	524.00
GOBP_REGULATION_OF_ENDOCYTOSIS	20.00	-0.36	-1.35	0.15	0.30	0.22	396.00
GOBP_RESPONSE_TO_DRUG	41.00	-0.30	-1.35	0.11	0.25	0.18	422.00
GOBP_METAL_ION_TRANSPORT	46.00	-0.29	-1.35	0.12	0.26	0.19	426.00
GOBP_T_CELL_DIFFERENTIATION	31.00	-0.32	-1.34	0.16	0.32	0.23	574.00
GOBP_POSITIVE_REGULATION_OF_CELLULAR_COMPONENT_BIOGENESIS	47.00	-0.29	-1.34	0.11	0.25	0.18	441.00
GOBP_CELLULAR_SENESCENCE	10.00	-0.45	-1.34	0.16	0.32	0.23	551.00
GOBP_REGULATION_OF_PEPTIDE_TRANSPORT	52.00	-0.28	-1.34	0.12	0.26	0.19	75.00
GOBP_DEVELOPMENT_OF_PRIMARY_FEMALE_SEXUAL_CHARACTERISTICS	15.00	-0.39	-1.34	0.16	0.32	0.23	378.00
GOBP_FEMALE_SEX_DIFFERENTIATION	15.00	-0.39	-1.34	0.16	0.32	0.23	378.00
GOBP_T_CELL_ACTIVATION_INVOLVED_IN_IMMUNE_RESPONSE	12.00	-0.43	-1.34	0.15	0.31	0.22	526.00
GOBP_NEGATIVE_REGULATION_OF_CELL_CELL_ADHESION	21.00	-0.36	-1.33	0.15	0.30	0.22	575.00
GOBP_ERK1_AND_ERK2_CASCADE	27.00	-0.33	-1.33	0.15	0.31	0.22	629.00
GOBP_POSITIVE_REGULATION_OF_PROTEIN_KINASE_ACTIVITY	58.00	-0.27	-1.33	0.12	0.26	0.19	550.00
GOBP_POSITIVE_REGULATION_OF_GTPASE_ACTIVITY	33.00	-0.31	-1.33	0.15	0.31	0.23	572.00
GOBP_LOCOMOTORY_BEHAVIOR	14.00	-0.40	-1.32	0.16	0.32	0.23	64.00
GOBP_REGULATION_OF_ANION_TRANSMEMBRANE_TRANSPORT	10.00	-0.45	-1.32	0.17	0.33	0.24	259.00
GOBP_CELLULAR_RESPONSE_TO_VASCULAR_ENDOTHELIAL_GROWTH_FACTOR_STIMULUS	10.00	-0.45	-1.32	0.17	0.33	0.24	351.00
GOBP_RESPONSE_TO_EXTRACELLULAR_STIMULUS	50.00	-0.28	-1.32	0.13	0.28	0.20	226.00
GOBP_ORGANIC_ACID_TRANSPORT	25.00	-0.34	-1.32	0.15	0.31	0.22	594.00
GOBP_POSITIVE_REGULATION_OF_CATALYTIC_ACTIVITY	135.00	-0.23	-1.32	0.11	0.24	0.17	508.00
GOBP_CONNECTIVE_TISSUE_DEVELOPMENT	24.00	-0.34	-1.32	0.15	0.31	0.22	533.00
GOBP_SENSORY_PERCEPTION_OF_PAIN	11.00	-0.43	-1.32	0.16	0.33	0.23	234.00
GOBP_OVULATION_CYCLE	10.00	-0.44	-1.32	0.17	0.33	0.24	275.00
GOBP_OSSIFICATION	46.00	-0.28	-1.31	0.13	0.28	0.20	422.00
GOBP_REGULATION_OF_LIPID_BIOSYNTHETIC_PROCESS	25.00	-0.33	-1.31	0.16	0.32	0.23	213.00
GOBP_POSITIVE_REGULATION_OF_NEUROGENESIS	21.00	-0.35	-1.31	0.16	0.32	0.23	402.00

GOBP_REGULATION_OF_CELL_SHAPE	15.00	-0.39	-1.31	0.17	0.34	0.24	667.00
GOBP_T_CELL_MEDIATED_IMMUNITY	15.00	-0.39	-1.31	0.17	0.34	0.24	242.00
GOBP_NEURON_DEVELOPMENT	97.00	-0.24	-1.31	0.11	0.24	0.18	416.00
GOBP_REGULATION_OF_SYSTEM_PROCESS	62.00	-0.26	-1.31	0.13	0.28	0.20	498.00
GOBP_PROTEIN_PHOSPHORYLATION	160.00	-0.22	-1.31	0.09	0.22	0.16	540.00
GOBP_REGULATION_OF_RESPONSE_TO_OXIDATIVE_STRESS	11.00	-0.43	-1.31	0.17	0.34	0.24	422.00
GOBP_EPITHELIAL_TO_MESENCHYMAL_TRANSITION	18.00	-0.36	-1.30	0.17	0.33	0.23	292.00
GOBP_REGULATION_OF_G_PROTEIN_COUPLED_RECEPTOR_SIGNALING_PATHWAY	10.00	-0.44	-1.30	0.18	0.34	0.25	75.00
GOBP_REGULATION_OF_GROWTH	58.00	-0.26	-1.30	0.14	0.29	0.21	355.00
GOBP_DEVELOPMENT_OF_PRIMARY_SEXUAL_CHARACTERISTICS	27.00	-0.32	-1.30	0.17	0.33	0.24	416.00
GOBP_ACTIN_POLYMERIZATION_OR_DEPOLYMERIZATION	18.00	-0.36	-1.30	0.17	0.33	0.24	406.00
GOBP_REGULATION_OF_ACTIN_FILAMENT_LENGTH	18.00	-0.36	-1.30	0.17	0.33	0.24	406.00
GOBP_CELL_CELL_ADHESION_VIA_PLASMA_MEMBRANE_ADHESION_MOLECULES	26.00	-0.32	-1.29	0.18	0.34	0.25	458.00
GOBP_RESPONSE_TO_STEROID_HORMONE	26.00	-0.32	-1.29	0.18	0.35	0.25	631.00
GOBP_TELENCEPHALON_DEVELOPMENT	17.00	-0.36	-1.29	0.19	0.35	0.25	432.00
GOBP_HOMOPHILIC_CELL_ADHESION_VIA_PLASMA_MEMBRANE_ADHESION_MOLECULES	13.00	-0.40	-1.29	0.19	0.35	0.25	388.00
GOBP_CELLULAR_RESPONSE_TO_MECHANICAL_STIMULUS	12.00	-0.41	-1.29	0.18	0.35	0.25	454.00
GOBP_REGULATION_OF_NOTCH_SIGNALING_PATHWAY	12.00	-0.41	-1.28	0.18	0.35	0.25	475.00
GOBP_BONE_REMODELING	12.00	-0.41	-1.28	0.18	0.35	0.25	612.00
GOBP_PATTERN_SPECIFICATION_PROCESS	36.00	-0.29	-1.28	0.17	0.33	0.24	28.00
GOBP_REGULATION_OF_BIOLOGICAL_PROCESS_INVOLVED_IN_SYMBIOTIC_INTERACTION	15.00	-0.38	-1.28	0.19	0.36	0.26	288.00
GOBP_POSITIVE_REGULATION_OF_PROTEIN_MODIFICATION_PROCESS	97.00	-0.23	-1.28	0.12	0.27	0.19	526.00
GOBP_NEGATIVE_REGULATION_OF_RESPONSE_TO_WOUNDING	12.00	-0.41	-1.28	0.19	0.36	0.26	230.00
GOBP_NEGATIVE_REGULATION_OF_WOUND_HEALING	12.00	-0.41	-1.28	0.19	0.36	0.26	230.00
GOBP_CARDIAC_CONDUCTION	18.00	-0.36	-1.28	0.18	0.34	0.25	534.00
GOBP_PROTEIN_KINASE_B_SIGNALING	26.00	-0.32	-1.28	0.19	0.36	0.26	574.00
GOBP_SKELETAL_SYSTEM_DEVELOPMENT	43.00	-0.28	-1.28	0.16	0.32	0.23	231.00
GOBP_STEROL_TRANSPORT	10.00	-0.43	-1.28	0.20	0.36	0.26	177.00
GOBP_NEURON_DIFFERENTIATION	117.00	-0.22	-1.27	0.14	0.29	0.21	467.00
GOBP_CARDIAC_MUSCLE_CELL_ACTION_POTENTIAL	13.00	-0.39	-1.27	0.19	0.36	0.26	212.00
GOBP_T_CELL_DIFFERENTIATION_INVOLVED_IN_IMMUNE_RESPONSE	10.00	-0.43	-1.27	0.20	0.36	0.26	526.00
GOBP_B_CELL_MEDIATED_IMMUNITY	16.00	-0.37	-1.27	0.20	0.37	0.26	242.00
GOBP_ACTIVATION_OF_PROTEIN_KINASE_ACTIVITY	32.00	-0.30	-1.27	0.19	0.36	0.26	396.00
GOBP_REGULATION_OF_PHOSPHATASE_ACTIVITY	13.00	-0.39	-1.27	0.20	0.36	0.26	627.00
GOBP_RESPONSE_TO_PEPTIDE	45.00	-0.27	-1.27	0.16	0.32	0.23	540.00
GOBP_POSITIVE_REGULATION_OF_HYDROLASE_ACTIVITY	75.00	-0.24	-1.27	0.14	0.30	0.21	469.00
GOBP_NEUROGENESIS	138.00	-0.22	-1.27	0.12	0.27	0.19	364.00
GOBP_CELLULAR_RESPONSE_TO_EXTRACELLULAR_STIMULUS	25.00	-0.32	-1.27	0.19	0.35	0.25	256.00
GOBP_POSITIVE_REGULATION_OF_CELL_DEVELOPMENT	30.00	-0.31	-1.27	0.19	0.36	0.26	441.00
GOBP_LIPID_LOCALIZATION	47.00	-0.27	-1.27	0.16	0.33	0.23	631.00
GOBP_RESPONSE_TO_ACID_CHEMICAL	13.00	-0.39	-1.27	0.20	0.36	0.26	168.00

GOBP_RESPONSE_TO_ORGANOPHOSPHORUS	15.00	-0.37	-1.26	0.21	0.37	0.27	411.00
GOBP_NEGATIVE_REGULATION_OF_IMMUNE_EFFECTOR_PROCESS	21.00	-0.34	-1.26	0.19	0.35	0.25	547.00
GOBP_REGULATION_OF_CELLULAR_RESPONSE_TO_STRESS	55.00	-0.26	-1.26	0.18	0.35	0.25	136.00
GOBP_RESPONSE_TO_INTERLEUKIN_1	20.00	-0.34	-1.26	0.19	0.36	0.26	696.00
GOBP_GLIAL_CELL_DEVELOPMENT	11.00	-0.41	-1.26	0.21	0.38	0.28	494.00
GOBP_BEHAVIOR	42.00	-0.28	-1.26	0.19	0.35	0.25	441.00
GOBP_REGULATION_OF_METAL_ION_TRANSPORT	29.00	-0.31	-1.25	0.20	0.37	0.26	553.00
GOBP_CELL_PART_MORPHOGENESIS	61.00	-0.25	-1.25	0.17	0.33	0.23	364.00
GOBP_CELL_CELL_JUNCTION_ASSEMBLY	17.00	-0.35	-1.25	0.22	0.39	0.28	262.00
GOBP_REGULATION_OF_COAGULATION	12.00	-0.40	-1.25	0.22	0.39	0.28	310.00
GOBP_RECEPTOR_INTERNALIZATION	14.00	-0.38	-1.25	0.21	0.38	0.27	396.00
GOBP_DIGESTIVE_SYSTEM_DEVELOPMENT	15.00	-0.37	-1.25	0.22	0.39	0.28	504.00
GOBP_CELLULAR_RESPONSE_TO_ORGANIC_CYCLIC_COMPOUND	45.00	-0.27	-1.25	0.18	0.34	0.25	631.00
GOBP_KERATINOCYTE_DIFFERENTIATION	13.00	-0.39	-1.25	0.21	0.38	0.27	359.00
GOBP_POSITIVE_REGULATION_OF_CATION_TRANSMEMBRANE_TRANSPORT	16.00	-0.36	-1.25	0.21	0.37	0.27	416.00
GOBP_ESTABLISHMENT_OF_ENDOTHELIAL_BARRIER	11.00	-0.41	-1.25	0.22	0.39	0.28	537.00
GOBP_CELLULAR_TRANSITION_METAL_ION_HOMEOSTASIS	12.00	-0.40	-1.25	0.22	0.39	0.28	612.00
GOBP_REGULATION_OF_SMALL_GTPASE_MEDIATED_SIGNAL_TRANSDUCTION	40.00	-0.28	-1.25	0.20	0.36	0.26	613.00
GOBP_RESPONSE_TO_ALCOHOL	26.00	-0.31	-1.25	0.22	0.39	0.28	504.00
GOBP_POSITIVE_REGULATION_OF_I_KAPPAB_KINASE_NF_KAPPAB_SIGNALING	20.00	-0.34	-1.25	0.21	0.38	0.27	694.00
GOBP_REGULATION_OF_LIPID_LOCALIZATION	15.00	-0.37	-1.24	0.22	0.39	0.28	614.00
GOBP_POSITIVE_REGULATION_OF_TRANSCRIPTION_BY_RNA_POLYMERASE_II	86.00	-0.23	-1.24	0.15	0.31	0.23	636.00
GOBP_CARBOHYDRATE_DERIVATIVE_CATABOLIC_PROCESS	18.00	-0.35	-1.24	0.21	0.38	0.27	279.00
GOBP_CELLULAR_RESPONSE_TO_CHEMICAL_STRESS	35.00	-0.29	-1.24	0.22	0.39	0.28	344.00
GOBP_CARDIAC_SEPTUM_DEVELOPMENT	13.00	-0.38	-1.24	0.23	0.39	0.28	666.00
GOBP_PATTERN_RECOGNITION_RECEPTOR_SIGNALING_PATHWAY	16.00	-0.36	-1.24	0.22	0.39	0.28	756.00
GOBP_TRANSMEMBRANE_RECEPTOR_PROTEIN_SERINE_THREONINE_KINASE_SIGNALING_PATHWAY	39.00	-0.28	-1.23	0.21	0.37	0.27	541.00
GOBP_DEVELOPMENTAL_GROWTH_INVOLVED_IN_MORPHOGENESIS	25.00	-0.31	-1.23	0.22	0.39	0.28	540.00
GOBP_REGULATION_OF_CELL_DEVELOPMENT	46.00	-0.26	-1.23	0.19	0.36	0.26	449.00
GOBP_REGULATION_OF_SIGNALING_RECEPTOR_ACTIVITY	13.00	-0.38	-1.23	0.23	0.39	0.28	91.00
GOBP_RESPONSE_TO_CAMP	11.00	-0.41	-1.23	0.24	0.40	0.29	574.00
GOBP_REGULATION_OF_RECEPTOR_MEDIATED_ENDOCYTOSIS	14.00	-0.37	-1.23	0.23	0.40	0.29	75.00
GOBP_REGULATION_OF_CYCLIN_DEPENDENT_PROTEIN_KINASE_ACTIVITY	10.00	-0.42	-1.23	0.22	0.39	0.28	560.00
GOBP_NEGATIVE_REGULATION_OF_NF_KAPPAB_TRANSCRIPTION_FACTOR_ACTIVITY	13.00	-0.38	-1.23	0.23	0.39	0.28	714.00
GOBP_DEFENSE_RESPONSE_TO_VIRUS	22.00	-0.32	-1.23	0.22	0.39	0.28	454.00
GOBP_REGULATION_OF_INFLAMMATORY_RESPONSE	37.00	-0.28	-1.23	0.22	0.39	0.28	505.00
GOBP_REGULATION_OF_DEPHOSPHORYLATION	15.00	-0.36	-1.23	0.22	0.39	0.28	627.00
GOBP_ERYTHROCYTE_HOMEOSTASIS	11.00	-0.40	-1.23	0.24	0.40	0.29	720.00
GOBP_REGULATION_OF_CELLULAR_COMPONENT_SIZE	36.00	-0.28	-1.23	0.22	0.39	0.28	406.00
GOBP_REGULATION_OF_MYELOID_CELL_DIFFERENTIATION	21.00	-0.33	-1.23	0.21	0.38	0.27	664.00

GOBP_CELL_CELL_JUNCTION_ORGANIZATION	25.00	-0.31	-1.22	0.23	0.40	0.29	453.00
GOBP_PLASMA_MEMBRANE_ORGANIZATION	12.00	-0.39	-1.22	0.26	0.42	0.30	556.00
GOBP_TOLL_LIKE_RECEPTOR_SIGNALING_PATHWAY	12.00	-0.39	-1.22	0.26	0.42	0.30	756.00
GOBP_ODONTOGENESIS_OF_DENTIN_CONTAINING_TOOTH	11.00	-0.40	-1.22	0.24	0.40	0.29	335.00
GOBP_HEART_GROWTH	10.00	-0.41	-1.22	0.23	0.40	0.29	300.00
GOBP_POSITIVE_REGULATION_OF_MOLECULAR_FUNCTION	162.00	-0.20	-1.22	0.16	0.32	0.23	508.00
GOBP_REGULATION_OF_CALCIUM_ION_TRANSMEMBRANE_TRANSPORTER_ACTIVITY	10.00	-0.41	-1.22	0.23	0.40	0.29	426.00
GOBP_CELL_PROJECTION_ORGANIZATION	127.00	-0.21	-1.22	0.18	0.35	0.25	416.00
GOBP_POSITIVE_REGULATION_OF_ACTIN_FILAMENT_POLYMERIZATION	10.00	-0.41	-1.22	0.24	0.40	0.29	296.00
GOBP_POSITIVE_REGULATION_OF_PROTEIN_POLYMERIZATION	10.00	-0.41	-1.22	0.24	0.40	0.29	296.00
GOBP_AXON_DEVELOPMENT	45.00	-0.26	-1.21	0.22	0.39	0.28	416.00
GOBP_MUSCLE_CELL_DIFFERENTIATION	30.00	-0.29	-1.21	0.25	0.42	0.30	553.00
GOBP_EMBRYONIC_ORGAN_MORPHOGENESIS	27.00	-0.30	-1.21	0.23	0.40	0.29	504.00
GOBP_TRANSMEMBRANE_TRANSPORT	130.00	-0.21	-1.21	0.18	0.35	0.25	464.00
GOBP_CELL_GROWTH	38.00	-0.27	-1.21	0.23	0.40	0.29	355.00
GOBP_RESPONSE_TO_CHEMOKINE	14.00	-0.37	-1.21	0.25	0.41	0.30	329.00
GOBP_RHO_PROTEIN_SIGNAL_TRANSDUCTION	16.00	-0.35	-1.21	0.24	0.40	0.29	613.00
GOBP_CELLULAR_RESPONSE_TO_ABIOTIC_STIMULUS	31.00	-0.29	-1.21	0.26	0.43	0.31	512.00
GOBP_CALCIUM_ION_TRANSMEMBRANE_TRANSPORT	21.00	-0.32	-1.21	0.23	0.40	0.29	571.00
GOBP_MONOCARBOXYLIC_ACID_TRANSPORT	18.00	-0.34	-1.21	0.23	0.40	0.29	511.00
GOBP_ROOF_OF_MOUTH_DEVELOPMENT	13.00	-0.37	-1.20	0.25	0.41	0.30	504.00
GOBP_GLYCEROLIPID_BIOSYNTHETIC_PROCESS	19.00	-0.33	-1.20	0.24	0.40	0.29	573.00
GOBP_REGULATION_OF_CELLULAR_COMPONENT_BIOGENESIS	91.00	-0.22	-1.20	0.20	0.37	0.26	449.00
GOBP_REGULATION_OF_CATION_TRANSMEMBRANE_TRANSPORT	29.00	-0.30	-1.20	0.25	0.42	0.30	426.00
GOBP_DEVELOPMENTAL_PROCESS_INVOLVED_IN_REPRODUCTION	75.00	-0.23	-1.20	0.21	0.38	0.27	250.00
GOBP_RESPONSE_TO_RETINOIC_ACID	15.00	-0.35	-1.20	0.24	0.41	0.29	550.00
GOBP_REPRODUCTION	110.00	-0.21	-1.20	0.20	0.36	0.26	378.00
GOBP_REGULATION_OF_OSSIFICATION	12.00	-0.38	-1.20	0.28	0.44	0.32	347.00
GOBP_ALPHA_AMINO_ACID_METABOLIC_PROCESS	14.00	-0.36	-1.20	0.26	0.42	0.30	154.00
GOBP_NEGATIVE_REGULATION_OF_DNA_BINDING_TRANSCRIPTION_FACTOR_ACTIVITY	23.00	-0.31	-1.20	0.24	0.41	0.29	663.00
GOBP_CELLULAR_COMPONENT_MORPHOGENESIS	68.00	-0.23	-1.20	0.22	0.39	0.28	364.00
GOBP_CELL_CYCLE_ARREST	16.00	-0.35	-1.19	0.25	0.42	0.30	287.00
GOBP_POSITIVE_REGULATION_OF_TRANSLATION	12.00	-0.38	-1.19	0.28	0.45	0.32	387.00
GOBP_ORGANIC_ACID_BIOSYNTHETIC_PROCESS	18.00	-0.33	-1.19	0.24	0.40	0.29	210.00
GOBP_CATION_TRANSPORT	95.00	-0.22	-1.19	0.21	0.38	0.27	507.00
GOBP_NEURAL_PRECURSOR_CELL_PROLIFERATION	15.00	-0.35	-1.19	0.26	0.43	0.31	521.00
GOBP_REGULATION_OF_EPITHELIAL_CELL_DIFFERENTIATION	13.00	-0.36	-1.18	0.27	0.44	0.31	463.00
GOBP_PROTEIN_POLYMERIZATION	23.00	-0.31	-1.18	0.25	0.42	0.30	406.00
GOBP_NEGATIVE_REGULATION_OF_INTRACELLULAR_SIGNAL_TRANSDUCTION	46.00	-0.25	-1.18	0.25	0.41	0.30	645.00
GOBP_MULTI_ORGANISM_PROCESS	75.00	-0.22	-1.18	0.23	0.40	0.29	424.00
GOBP_NEGATIVE_REGULATION_OF_ANION_TRANSPORT	20.00	-0.32	-1.18	0.26	0.43	0.31	174.00
GOBP_CARDIAC_MUSCLE_CELL_MEMBRANE_REPOLARIZATION	10.00	-0.40	-1.17	0.27	0.43	0.31	416.00

GOBP_MEMBRANE_REPOLARIZATION	10.00	-0.40	-1.17	0.27	0.43	0.31	416.00
GOBP_MEMBRANE_REPOLARIZATION_DURING_ACTION_POTENTIAL	10.00	-0.40	-1.17	0.27	0.43	0.31	416.00
GOBP_CELLULAR_RESPONSE_TO_CARBOHYDRATE_STIMULUS	10.00	-0.40	-1.17	0.27	0.43	0.31	292.00
GOBP_GLYCOPROTEIN_METABOLIC_PROCESS	38.00	-0.26	-1.17	0.27	0.44	0.31	305.00
GOBP_POSITIVE_REGULATION_OF_LEUKOCYTE_PROLIFERATION	16.00	-0.34	-1.17	0.27	0.44	0.32	570.00
GOBP_POSITIVE_REGULATION_OF_CELLULAR_AMIDE_METABOLIC_PROCESS	16.00	-0.34	-1.16	0.28	0.45	0.32	494.00
GOBP_FC_RECEPTOR_SIGNALING_PATHWAY	15.00	-0.34	-1.16	0.28	0.45	0.32	614.00
GOBP_ACTIN_FILAMENT_BUNDLE_ORGANIZATION	16.00	-0.34	-1.16	0.28	0.45	0.32	416.00
GOBP_NEGATIVE_REGULATION_OF_COAGULATION	10.00	-0.39	-1.16	0.28	0.45	0.32	230.00
GOBP_RESPONSE_TO_BMP	16.00	-0.34	-1.16	0.29	0.45	0.33	340.00
GOBP_INORGANIC_ANION_TRANSPORT	17.00	-0.33	-1.15	0.29	0.46	0.33	511.00
GOBP_REGULATION_OF_ORGAN_GROWTH	10.00	-0.39	-1.15	0.28	0.45	0.32	300.00
GOBP_SENSORY_PERCEPTION	66.00	-0.23	-1.15	0.27	0.43	0.31	234.00
GOBP_POSITIVE_REGULATION_OF_T_CELL_PROLIFERATION	13.00	-0.36	-1.15	0.30	0.47	0.34	699.00
GOBP_TUMOR_NECROSIS_FACTOR_MEDIATED_SIGNALING_PATHWAY	25.00	-0.29	-1.15	0.29	0.46	0.33	970.00
GOBP_CELLULAR_RESPONSE_TO_NITROGEN_COMPOUND	50.00	-0.24	-1.15	0.26	0.43	0.31	416.00
GOBP_POSITIVE_REGULATION_OF_CELL_PROJECTION_ORGANIZATION	30.00	-0.28	-1.14	0.31	0.49	0.35	550.00
GOBP_CIRCADIAN_RHYTHM	21.00	-0.31	-1.14	0.30	0.47	0.34	287.00
GOBP_REGULATION_OF_PEPTIDYL_SERINE_PHOSPHORYLATION	11.00	-0.38	-1.14	0.31	0.48	0.35	426.00
GOBP_REGULATION_OF_CELL_PROJECTION_ORGANIZATION	55.00	-0.23	-1.14	0.28	0.45	0.32	416.00
GOBP_REGULATION_OF_CELLULAR_KETONE_METABOLIC_PROCESS	13.00	-0.35	-1.13	0.32	0.49	0.35	210.00
GOBP_NEPHRON_DEVELOPMENT	23.00	-0.29	-1.13	0.30	0.48	0.34	252.00
GOBP_IRON_ION_TRANSPORT	12.00	-0.36	-1.13	0.33	0.50	0.36	381.00
GOBP_RESPONSE_TO_HEAT	14.00	-0.34	-1.13	0.32	0.49	0.35	281.00
GOBP_STEROID_BIOSYNTHETIC_PROCESS	17.00	-0.32	-1.13	0.32	0.49	0.35	177.00
GOBP_POSITIVE_REGULATION_OF_CALCIUM_ION_TRANSPORT	17.00	-0.32	-1.13	0.32	0.49	0.35	553.00
GOBP_CARBOHYDRATE_CATABOLIC_PROCESS	19.00	-0.31	-1.13	0.30	0.47	0.34	348.00
GOBP_NEURON_PROJECTION_EXTENSION	17.00	-0.32	-1.13	0.32	0.49	0.35	402.00
GOBP_POSITIVE_REGULATION_OF_TRANSFERASE_ACTIVITY	68.00	-0.22	-1.13	0.30	0.47	0.34	550.00
GOBP_POSITIVE_REGULATION_OF_NERVOUS_SYSTEM_DEVELOPMENT	25.00	-0.29	-1.13	0.32	0.49	0.35	402.00
GOBP_REGULATION_OF_VESICLE_MEDIATED_TRANSPORT	57.00	-0.23	-1.12	0.31	0.49	0.35	101.00
GOBP_REGULATION_OF_STEROID_BIOSYNTHETIC_PROCESS	15.00	-0.33	-1.12	0.32	0.49	0.35	121.00
GOBP_REGULATION_OF_STEROID_METABOLIC_PROCESS	15.00	-0.33	-1.12	0.32	0.49	0.35	121.00
GOBP_PROTEIN_LOCALIZATION_TO_ENDOPLASMIC_RETICULUM	12.00	-0.36	-1.12	0.34	0.51	0.36	43.00
GOBP_REGULATION_OF_NEURON_PROJECTION_DEVELOPMENT	36.00	-0.26	-1.12	0.32	0.49	0.35	416.00
GOBP_T_CELL_RECEPTOR_SIGNALING_PATHWAY	17.00	-0.32	-1.12	0.33	0.50	0.36	700.00
GOBP_CELLULAR_BIOGENIC_AMINE_METABOLIC_PROCESS	12.00	-0.36	-1.12	0.34	0.51	0.37	379.00
GOBP_REGULATION_OF_ADAPTIVE_IMMUNE_RESPONSE	23.00	-0.29	-1.12	0.32	0.49	0.35	547.00

GOBP_POSITIVE_REGULATION_OF_ADAPTIVE_IMMUNE_RESPONSE	13.00	-0.34	-1.12	0.33	0.50	0.36	664.00
GOBP_POSITIVE_REGULATION_OF_PLASMA_MEMBRANE_BOUNDED_CELL_PROJECTION_ASSEMBLY	10.00	-0.38	-1.12	0.32	0.49	0.35	384.00
GOBP_NEGATIVE_REGULATION_OF_GROWTH	19.00	-0.31	-1.12	0.31	0.49	0.35	209.00
GOBP_CARBOHYDRATE_TRANSPORT	10.00	-0.38	-1.12	0.32	0.49	0.35	500.00
GOBP_MODULATION_OF_PROCESS_OF_OTHER_ORGANISM_INVOLVED_IN_SYMBIOTIC_INTERACTION	10.00	-0.38	-1.12	0.32	0.49	0.35	526.00
GOBP_RESPONSE_TO_PURINE_CONTAINING_COMPOUND	16.00	-0.32	-1.12	0.33	0.50	0.36	411.00
GOBP_REGULATION_OF_TRANSPORTER_ACTIVITY	22.00	-0.29	-1.11	0.32	0.49	0.35	239.00
GOBP_REGULATION_OF_CYSTEINE_TYPE_ENDOPEPTIDASE_ACTIVITY	25.00	-0.28	-1.11	0.34	0.51	0.37	551.00
GOBP_OSTEOBLAST_DIFFERENTIATION	20.00	-0.30	-1.11	0.32	0.49	0.35	357.00
GOBP_REGULATION_OF_NIK_NF_KAPPAB_SIGNALING	12.00	-0.36	-1.11	0.35	0.51	0.37	645.00
GOBP_ENSHEATHMENT_OF_NEURONS	11.00	-0.37	-1.11	0.34	0.51	0.37	494.00
GOBP_REGULATION_OF_CELL_CYCLE_G1_S_PHASE_TRANSITION	12.00	-0.35	-1.11	0.35	0.51	0.37	336.00
GOBP_VASCULOGENESIS	15.00	-0.33	-1.11	0.34	0.51	0.37	613.00
GOBP_PRODUCTION_OF_MOLECULAR_MEDIATOR_INVOLVED_IN_INFLAMMATORY_RESPONSE	13.00	-0.34	-1.11	0.34	0.51	0.37	699.00
GOBP_IMPORT_ACROSS_PLASMA_MEMBRANE	21.00	-0.30	-1.11	0.32	0.49	0.35	450.00
GOBP_CATION_TRANSMEMBRANE_TRANSPORT	64.00	-0.22	-1.11	0.34	0.51	0.37	457.00
GOBP_REGULATION_OF_NEUROGENESIS	31.00	-0.26	-1.10	0.36	0.53	0.38	422.00
GOBP_SEX_DIFFERENTIATION	30.00	-0.27	-1.10	0.35	0.51	0.37	416.00
GOBP_NEGATIVE_REGULATION_OF_INFLAMMATORY_RESPONSE	21.00	-0.29	-1.10	0.33	0.50	0.36	449.00
GOBP_EMBRYO_DEVELOPMENT_ENDING_IN_BIRTH_OR_EGG_HATCHING	56.00	-0.22	-1.10	0.33	0.50	0.36	316.00
GOBP_REGULATION_OF_STEM_CELL_DIFFERENTIATION	12.00	-0.35	-1.10	0.36	0.52	0.38	192.00
GOBP_REGULATION_OF_CELL_MORPHOGENESIS	29.00	-0.27	-1.10	0.34	0.51	0.37	550.00
GOBP_REGULATION_OF_B_CELL_ACTIVATION	10.00	-0.37	-1.10	0.34	0.51	0.37	574.00
GOBP_POSITIVE_REGULATION_OF_ORGANELLE_ORGANIZATION	58.00	-0.22	-1.09	0.34	0.51	0.37	416.00
GOBP_NEGATIVE_REGULATION_OF_WNT_SIGNALING_PATHWAY	16.00	-0.32	-1.09	0.34	0.51	0.37	343.00
GOBP_SECOND_MESSENGER_MEDIATED_SIGNALING	24.00	-0.28	-1.09	0.35	0.51	0.37	426.00
GOBP_BONE_MINERALIZATION	10.00	-0.37	-1.09	0.35	0.51	0.37	555.00
GOBP_REGULATION_OF_BONE_MINERALIZATION	10.00	-0.37	-1.09	0.35	0.51	0.37	555.00
GOBP_POSITIVE_REGULATION_OF_APOPTOTIC_SIGNALING_PATHWAY	13.00	-0.34	-1.09	0.35	0.52	0.37	356.00
GOBP_POSITIVE_REGULATION_OF_PEPTIDASE_ACTIVITY	23.00	-0.28	-1.09	0.34	0.51	0.37	443.00
GOBP_REGULATION_OF_GTPASE_ACTIVITY	44.00	-0.23	-1.09	0.35	0.51	0.37	629.00
GOBP_PROTEIN_DEPHOSPHORYLATION	23.00	-0.28	-1.09	0.34	0.51	0.37	139.00
GOBP_REGULATION_OF_TRANSMEMBRANE_RECEPTOR_PROTEIN_SERINE_THREONINE_KINASE_SIGNALING_PATHWAY	27.00	-0.27	-1.08	0.37	0.54	0.39	354.00
GOBP_CELL_MORPHOGENESIS_INVOLVED_IN_NEURON_DIFFERENTIATION	48.00	-0.23	-1.08	0.34	0.51	0.37	409.00
GOBP_REGULATION_OF_NERVOUS_SYSTEM_DEVELOPMENT	36.00	-0.25	-1.08	0.37	0.54	0.39	422.00
GOBP_REGULATION_OF_STRESS_ACTIVATED_PROTEIN_KINASE_SIGNALING_CASCADE	15.00	-0.32	-1.08	0.37	0.54	0.39	248.00
GOBP_CELL_KILLING	20.00	-0.29	-1.08	0.35	0.51	0.37	242.00
GOBP_REGULATION_OF_LIPASE_ACTIVITY	12.00	-0.34	-1.08	0.38	0.55	0.39	324.00

GOBP_REGULATION_OF_RESPONSE_TO_DNA_DAMAGE_STIMULUS	12.00	-0.34	-1.08	0.38	0.55	0.39	103.00
GOBP_REGULATION_OF_RHO_PROTEIN_SIGNAL_TRANSDUCTION	12.00	-0.34	-1.08	0.38	0.55	0.40	613.00
GOBP_RESPIRATORY_SYSTEM_DEVELOPMENT	21.00	-0.29	-1.08	0.35	0.52	0.37	538.00
GOBP_MAMMARY_GLAND_DEVELOPMENT	14.00	-0.32	-1.07	0.38	0.55	0.40	504.00
GOBP_REGULATION_OF_ACTOMYOSIN_STRUCTURE_ORGANIZATION	11.00	-0.35	-1.07	0.39	0.55	0.40	351.00
GOBP_INORGANIC_ION_TRANSMEMBRANE_TRANSPORT	66.00	-0.21	-1.07	0.37	0.54	0.39	457.00
GOBP_RECEPTOR_METABOLIC_PROCESS	18.00	-0.30	-1.07	0.39	0.55	0.40	75.00
GOBP_ACTOMYOSIN_STRUCTURE_ORGANIZATION	24.00	-0.28	-1.07	0.39	0.56	0.40	351.00
GOBP_NEGATIVE_REGULATION_OF_LEUKOCYTE_MEDIATED_IMMUNITY	10.00	-0.36	-1.07	0.39	0.55	0.40	242.00
GOBP_SENSORY_PERCEPTION_OF_LIGHT_STIMULUS	28.00	-0.26	-1.07	0.38	0.55	0.40	423.00
GOBP_RESPONSE_TO_LEUKEMIA_INHIBITORY_FACTOR	10.00	-0.36	-1.07	0.39	0.56	0.40	284.00
GOBP_REGULATION_OF_ACTIN_CYTOSKELETON_REORGANIZATION	11.00	-0.35	-1.07	0.39	0.56	0.40	509.00
GOBP_REGULATION_OF_DEVELOPMENTAL_GROWTH	30.00	-0.26	-1.06	0.40	0.57	0.41	504.00
GOBP_HORMONE_METABOLIC_PROCESS	12.00	-0.34	-1.06	0.39	0.56	0.40	292.00
GOBP_CELLULAR_RESPONSE_TO_HORMONE_STIMULUS	42.00	-0.23	-1.06	0.39	0.56	0.40	117.00
GOBP_REGULATION_OF_ACTIN_FILAMENT_BUNDLE_ASSEMBLY	12.00	-0.34	-1.06	0.39	0.56	0.40	416.00
GOBP_CELLULAR_RESPONSE_TO_HEAT	11.00	-0.35	-1.06	0.40	0.56	0.41	174.00
GOBP_POSITIVE_REGULATION_OF_NEURON_PROJECTION_DEVELOPMENT	14.00	-0.32	-1.06	0.40	0.56	0.41	228.00
GOBP_REGULATION_OF_CELL_MORPHOGENESIS_INVOLVED_IN_DIFFERENTIATION	11.00	-0.35	-1.06	0.40	0.56	0.41	4.00
GOBP_POSITIVE_REGULATION_OF_DEFENSE_RESPONSE	44.00	-0.23	-1.06	0.40	0.56	0.41	616.00
GOBP_POSITIVE_REGULATION_OF_SECRETION	28.00	-0.26	-1.05	0.40	0.56	0.41	405.00
GOBP_REGULATION_OF_LIPID_TRANSPORT	13.00	-0.32	-1.05	0.39	0.56	0.40	614.00
GOBP_ORGANIC_HYDROXY_COMPOUND_METABOLIC_PROCESS	48.00	-0.22	-1.05	0.38	0.54	0.39	489.00
GOBP_DEPHOSPHORYLATION	36.00	-0.24	-1.05	0.41	0.57	0.41	155.00
GOBP_ACTIVATION_OF_IMMUNE_RESPONSE	39.00	-0.24	-1.05	0.40	0.56	0.40	616.00
GOBP_PLATELET_AGGREGATION	10.00	-0.35	-1.05	0.41	0.57	0.41	575.00
GOBP_LIPID_BIOSYNTHETIC_PROCESS	57.00	-0.21	-1.05	0.40	0.57	0.41	213.00
GOBP_REGULATION_OF_PROTEIN_DEPHOSPHORYLATION	10.00	-0.35	-1.05	0.41	0.57	0.41	507.00
GOBP_MEMBRANE_ORGANIZATION	71.00	-0.20	-1.05	0.41	0.57	0.41	306.00
GOBP_RESPONSE_TO_PEPTIDE_HORMONE	35.00	-0.24	-1.05	0.41	0.57	0.41	82.00
GOBP_POST_EMBRYONIC_DEVELOPMENT	10.00	-0.35	-1.04	0.41	0.57	0.41	358.00
GOBP_POSITIVE_REGULATION_OF_MYELOID_CELL_DIFFERENTIATION	10.00	-0.35	-1.04	0.41	0.57	0.41	664.00
GOBP_NEGATIVE_REGULATION_OF_DEFENSE_RESPONSE	32.00	-0.25	-1.04	0.41	0.57	0.41	449.00
GOBP_POSITIVE_REGULATION_OF_BIOSYNTHETIC_PROCESS	153.00	-0.17	-1.04	0.41	0.57	0.41	619.00
GOBP_REGULATION_OF_RAS_PROTEIN_SIGNAL_TRANSDUCTION	22.00	-0.27	-1.04	0.39	0.56	0.40	651.00
GOBP_CELLULAR_RESPONSE_TO_OXYGEN_LEVELS	19.00	-0.29	-1.04	0.41	0.57	0.41	104.00
GOBP_REGULATION_OF_DNA_BINDING	13.00	-0.32	-1.03	0.41	0.57	0.41	236.00
GOBP_ORGANIC_ACID_TRANSMEMBRANE_TRANSPORT	11.00	-0.34	-1.03	0.43	0.59	0.42	592.00
GOBP_VACUOLE_ORGANIZATION	21.00	-0.28	-1.03	0.41	0.57	0.41	610.00

GOBP_INTRINSIC_APOPTOTIC_SIGNALING_PATHWAY	24.00	-0.27	-1.03	0.42	0.58	0.42	320.00
GOBP_DEVELOPMENTAL_CELL_GROWTH	19.00	-0.28	-1.03	0.43	0.59	0.42	540.00
GOBP_REGULATION_OF_DNA_METABOLIC_PROCESS	24.00	-0.27	-1.03	0.42	0.58	0.42	52.00
GOBP_CELLULAR_RESPONSE_TO_STEROID_HORMONE_STIMULUS	14.00	-0.31	-1.03	0.44	0.59	0.43	631.00
GOBP_NEGATIVE_REGULATION_OF_IMMUNE_RESPONSE	22.00	-0.27	-1.03	0.40	0.57	0.41	547.00
GOBP_MULTICELLULAR_ORGANISM_REPRODUCTION	50.00	-0.22	-1.03	0.42	0.57	0.41	286.00
GOBP_POSITIVE_REGULATION_OF_IMMUNE_EFFECTOR_PROCESS	24.00	-0.26	-1.02	0.44	0.59	0.43	547.00
GOBP_NUCLEOSIDE_DIPHOSPHATE_METABOLIC_PROCESS	10.00	-0.34	-1.02	0.43	0.59	0.42	601.00
GOBP_REGULATION_OF_CELL_JUNCTION_ASSEMBLY	21.00	-0.27	-1.02	0.42	0.58	0.41	466.00
GOBP_ANTIGEN_PROCESSING_AND_PRESENTATION_OF_EXOGENOUS_PEPTIDE_ANTIGEN_VIA_MHC_CLASS_I	13.00	-0.31	-1.02	0.43	0.59	0.42	939.00
GOBP_ANTIGEN_PROCESSING_AND_PRESENTATION_OF_PEPTIDE_ANTIGEN_VIA_MHC_CLASS_I	13.00	-0.31	-1.02	0.43	0.59	0.42	939.00
GOBP_NEGATIVE_REGULATION_OF_PHOSPHORUS_METABOLIC_PROCESS	42.00	-0.22	-1.02	0.47	0.61	0.44	627.00
GOBP_REGULATION_OF_MRNA_METABOLIC_PROCESS	20.00	-0.27	-1.01	0.42	0.57	0.41	993.00
GOBP_NEGATIVE_REGULATION_OF_CYTOSKELETON_ORGANIZATION	10.00	-0.34	-1.01	0.44	0.59	0.43	541.00
GOBP_REGULATION_OF_CATION_CHANNEL_ACTIVITY	15.00	-0.30	-1.01	0.44	0.59	0.43	426.00
GOBP_MUCOPOLYSACCHARIDE_METABOLIC_PROCESS	11.00	-0.33	-1.01	0.46	0.61	0.44	136.00
GOBP_NEGATIVE_REGULATION_OF_TRANSCRIPTION_BY_RNA_POLYMERASE_II	70.00	-0.19	-1.01	0.47	0.61	0.44	735.00
GOBP_BIOMINERALIZATION	13.00	-0.31	-1.01	0.45	0.60	0.43	347.00
GOBP_FOREBRAIN_DEVELOPMENT	27.00	-0.25	-1.01	0.45	0.60	0.43	462.00
GOBP_PROTEIN_HOMOOLIGOMERIZATION	18.00	-0.28	-1.01	0.46	0.61	0.43	438.00
GOBP_LEUKOCYTE_MEDIATED_CYTOTOXICITY	15.00	-0.30	-1.00	0.46	0.61	0.43	242.00
GOBP_CELLULAR_KETONE_METABOLIC_PROCESS	16.00	-0.29	-1.00	0.45	0.60	0.43	210.00
GOBP_REGULATION_OF_CIRCADIAN_RHYTHM	11.00	-0.33	-1.00	0.47	0.61	0.44	287.00
GOBP_REGULATION_OF_DEFENSE_RESPONSE	75.00	-0.19	-1.00	0.49	0.63	0.45	650.00
GOBP_REGULATION_OF_PROTEIN_MATURATION	10.00	-0.34	-1.00	0.45	0.60	0.43	551.00
GOBP_MONOCARBOXYLIC_ACID_BIOSYNTHETIC_PROCESS	12.00	-0.32	-1.00	0.46	0.61	0.44	210.00
GOBP_CELL_DEATH_IN_RESPONSE_TO_OXIDATIVE_STRESS	10.00	-0.34	-1.00	0.45	0.60	0.43	136.00
GOBP_LYMPHOCYTE_ACTIVATION_INVOLVED_IN_IMMUNE_RESPONSE	19.00	-0.27	-0.99	0.47	0.62	0.44	526.00
GOBP_CENTRAL_NERVOUS_SYSTEM_DEVELOPMENT	82.00	-0.18	-0.99	0.49	0.64	0.46	432.00
GOBP_ESTABLISHMENT_OR_MAINTENANCE_OF_CELL_POLARITY	21.00	-0.26	-0.99	0.45	0.60	0.43	425.00
GOBP_NEGATIVE_REGULATION_OF_SUPRAMOLECULAR_FIBER_ORGANIZATION	12.00	-0.32	-0.99	0.47	0.61	0.44	541.00
GOBP_HEMATOPOIETIC_PROGENITOR_CELL_DIFFERENTIATION	20.00	-0.27	-0.99	0.46	0.61	0.43	192.00
GOBP_PEPTIDYL_AMINO_ACID_MODIFICATION	98.00	-0.18	-0.99	0.49	0.64	0.46	525.00
GOBP_HEAD_DEVELOPMENT	64.00	-0.19	-0.99	0.50	0.64	0.46	441.00
GOBP_NEGATIVE_REGULATION_OF_CELLULAR_CATABOLIC_PROCESS	21.00	-0.26	-0.99	0.45	0.60	0.43	189.00
GOBP_VENTRICULAR_SEPTUM_DEVELOPMENT	10.00	-0.33	-0.98	0.47	0.61	0.44	666.00
GOBP_POSITIVE_REGULATION_OF_PROTEOLYSIS	38.00	-0.22	-0.98	0.50	0.64	0.46	526.00

GOBP_FC_EPSILON_RECEPTOR_SIGNALING_PATHWAY	12.00	-0.31	-0.98	0.48	0.62	0.45	939.00
GOBP_CELLULAR_RESPONSE_TO_PEPTIDE	29.00	-0.24	-0.98	0.49	0.63	0.46	82.00
GOBP_CARDIAC_MUSCLE_TISSUE_DEVELOPMENT	24.00	-0.25	-0.98	0.49	0.63	0.46	211.00
GOBP_ANTIGEN_PROCESSING_AND_PRESENTATION	30.00	-0.24	-0.98	0.48	0.62	0.45	568.00
GOBP_STEROID_METABOLIC_PROCESS	24.00	-0.25	-0.98	0.49	0.64	0.46	177.00
GOBP_REGULATION_OF_PROTEIN_LOCALIZATION	75.00	-0.19	-0.98	0.53	0.67	0.48	436.00
GOBP_NEGATIVE_REGULATION_OF_CANONICAL_WNT_SIGNALING_PATHWAY	14.00	-0.30	-0.97	0.49	0.63	0.45	343.00
GOBP_CARBOHYDRATE_DERIVATIVE_METABOLIC_PROCESS	94.00	-0.18	-0.97	0.53	0.67	0.48	311.00
GOBP_CELL_CYCLE_G1_S_PHASE_TRANSITION	20.00	-0.26	-0.96	0.49	0.64	0.46	336.00
GOBP_STRESS_FIBER_ASSEMBLY	10.00	-0.32	-0.96	0.50	0.64	0.46	351.00
GOBP_REGULATION_OF_TRANSFERASE_ACTIVITY	92.00	-0.17	-0.96	0.55	0.68	0.49	560.00
GOBP_PROTEIN_LOCALIZATION_TO_PLASMA_MEMBRANE	27.00	-0.24	-0.96	0.51	0.65	0.47	569.00
GOBP_INORGANIC_ION_IMPORT_ACROSS_PLASMA_MEMBRANE	12.00	-0.30	-0.95	0.51	0.65	0.47	212.00
GOBP_EXPORT_ACROSS_PLASMA_MEMBRANE	15.00	-0.28	-0.95	0.51	0.65	0.47	590.00
GOBP_NEGATIVE_REGULATION_OF_CATABOLIC_PROCESS	26.00	-0.24	-0.95	0.51	0.65	0.47	189.00
GOBP_REGULATION_OF_RESPONSE_TO_CYTOKINE_STIMULUS	17.00	-0.27	-0.95	0.53	0.67	0.48	653.00
GOBP_VITAMIN_METABOLIC_PROCESS	10.00	-0.32	-0.95	0.52	0.66	0.47	614.00
GOBP_REGULATION_OF_MUSCLE_CELL_DIFFERENTIATION	11.00	-0.31	-0.95	0.53	0.67	0.48	720.00
GOBP_REGULATION_OF_MRNA_CATABOLIC_PROCESS	14.00	-0.29	-0.94	0.52	0.66	0.48	976.00
GOBP_PYRUVATE_METABOLIC_PROCESS	10.00	-0.32	-0.94	0.52	0.66	0.48	311.00
GOBP_REGULATION_OF_INTRINSIC_APOPTOTIC_SIGNALING_PATHWAY	15.00	-0.28	-0.94	0.53	0.67	0.48	561.00
GOBP_GLIAL_CELL_DIFFERENTIATION	18.00	-0.26	-0.94	0.55	0.68	0.49	376.00
GOBP_MULTICELLULAR_ORGANISM_GROWTH	12.00	-0.30	-0.94	0.52	0.66	0.48	153.00
GOBP_REGULATION_OF_BIOMINERALIZATION	11.00	-0.31	-0.94	0.54	0.68	0.49	347.00
GOBP_TRANSFORMING_GROWTH_FACTOR_BETA_RECEPTOR_SIGNALING_PATHWAY	27.00	-0.23	-0.93	0.53	0.67	0.48	636.00
GOBP_PROTEIN_TARGETING_TO_MEMBRANE	14.00	-0.28	-0.93	0.54	0.68	0.49	86.00
GOBP_APOPTOTIC_MITOCHONDRIAL_CHANGES	11.00	-0.31	-0.93	0.55	0.68	0.49	356.00
GOBP_SKELETAL_MUSCLE_ORGAN_DEVELOPMENT	11.00	-0.31	-0.93	0.55	0.68	0.49	701.00
GOBP_MESODERM_DEVELOPMENT	11.00	-0.31	-0.93	0.55	0.68	0.49	588.00
GOBP_IN_UTERO_EMBRYONIC_DEVELOPMENT	41.00	-0.20	-0.93	0.57	0.70	0.50	316.00
GOBP_REGULATION_OF_ANIMAL_ORGAN_MORPHOGENESIS	10.00	-0.31	-0.93	0.55	0.68	0.49	939.00
GOBP_POSITIVE_REGULATION_OF_NUCLEOBASE_CONTAINING_COMPOUND_METABOLIC_PROCESS	131.00	-0.16	-0.93	0.60	0.72	0.52	681.00
GOBP_REGULATION_OF_JNK_CASCADE	10.00	-0.31	-0.92	0.55	0.68	0.49	248.00
GOBP_RESPONSE_TO_CALCIUM_ION	17.00	-0.26	-0.92	0.55	0.68	0.49	381.00
GOBP_NON_CANONICAL_WNT_SIGNALING_PATHWAY	10.00	-0.31	-0.92	0.55	0.68	0.49	273.00
GOBP_ENDOPLASMIC_RETICULUM_TO_GOLGI_VESICLE_MEDIATED_TRANSPORT	10.00	-0.31	-0.92	0.55	0.68	0.49	91.00
GOBP_LIPID_METABOLIC_PROCESS	107.00	-0.16	-0.92	0.59	0.72	0.51	507.00
GOBP_SKELETAL_SYSTEM_MORPHOGENESIS	18.00	-0.26	-0.92	0.57	0.70	0.51	533.00
GOBP_PROTEIN_COMPLEX_OLIGOMERIZATION	22.00	-0.24	-0.92	0.54	0.68	0.49	579.00
GOBP_RESPONSE_TO_INSULIN	22.00	-0.24	-0.91	0.55	0.68	0.49	523.00
GOBP_CARBOHYDRATE_METABOLIC_PROCESS	49.00	-0.19	-0.91	0.61	0.73	0.53	348.00

GOBP_NEGATIVE_REGULATION_OF_PROTEIN_SERINE_THREONINE_KINASE_ACTIVITY	13.00	-0.28	-0.90	0.57	0.71	0.51	613.00
GOBP_FATTY_ACID_TRANSPORT	13.00	-0.28	-0.90	0.58	0.71	0.51	592.00
GOBP_NEGATIVE_REGULATION_OF_INNATE_IMMUNE_RESPONSE	14.00	-0.27	-0.90	0.57	0.70	0.50	272.00
GOBP_POSITIVE_REGULATION_OF_PROTEIN_LOCALIZATION_TO_MEMBRANE	14.00	-0.27	-0.90	0.57	0.70	0.50	388.00
GOBP_POSITIVE_REGULATION_OF_CYSTEINE_TYPE_ENDOPEPTIDASE_ACTIVITY	18.00	-0.25	-0.90	0.59	0.72	0.51	505.00
GOBP_POSITIVE_REGULATION_OF_LIPASE_ACTIVITY	10.00	-0.30	-0.89	0.58	0.71	0.51	324.00
GOBP_REGULATION_OF_PHOSPHOLIPASE_ACTIVITY	10.00	-0.30	-0.89	0.58	0.71	0.51	324.00
GOBP_REGULATION_OF_WNT_SIGNALING_PATHWAY	28.00	-0.22	-0.89	0.57	0.71	0.51	273.00
GOBP_NEGATIVE_REGULATION_OF_RESPONSE_TO_BIOTIC_STIMULUS	17.00	-0.25	-0.89	0.60	0.72	0.52	424.00
GOBP_REGULATION_OF_BINDING	29.00	-0.22	-0.89	0.59	0.72	0.52	236.00
GOBP_REGULATION_OF_PROTEIN_LOCALIZATION_TO_CELL_PERIPHERY	13.00	-0.27	-0.89	0.60	0.73	0.52	651.00
GOBP_IMMUNE_RESPONSE_REGULATING_SIGNALING_PATHWAY	34.00	-0.21	-0.88	0.60	0.73	0.52	700.00
GOBP_SIGNAL_TRANSDUCTION_BY_P53_CLASS_MEDIATOR	20.00	-0.24	-0.88	0.62	0.74	0.53	286.00
GOBP_GLYCOPROTEIN_BIOSYNTHETIC_PROCESS	31.00	-0.21	-0.88	0.58	0.71	0.51	154.00
GOBP_LYTIC_VACUOLE_ORGANIZATION	13.00	-0.27	-0.88	0.61	0.73	0.53	527.00
GOBP_TRANSITION_METAL_ION_TRANSPORT	17.00	-0.25	-0.88	0.61	0.73	0.53	612.00
GOBP_MUSCLE_TISSUE_DEVELOPMENT	34.00	-0.20	-0.88	0.61	0.73	0.53	619.00
GOBP_REGULATION_OF_AXONOGENESIS	14.00	-0.27	-0.88	0.59	0.72	0.52	501.00
GOBP_POSTTRANSCRIPTIONAL_REGULATION_OF_GENE_EXPRESSION	42.00	-0.19	-0.87	0.66	0.76	0.55	1001.00
GOBP_TYPE_I_INTERFERON_PRODUCTION	14.00	-0.26	-0.87	0.60	0.73	0.52	819.00
GOBP_CARTILAGE_DEVELOPMENT	17.00	-0.25	-0.87	0.62	0.74	0.53	292.00
GOBP_STRIATED_MUSCLE_CELL_DIFFERENTIATION	24.00	-0.22	-0.87	0.62	0.73	0.53	553.00
GOBP_NEPHRON_EPITHELIUM_DEVELOPMENT	14.00	-0.26	-0.87	0.60	0.73	0.52	330.00
GOBP_REGULATION_OF_CELLULAR_AMIDE_METABOLIC_PROCESS	31.00	-0.21	-0.87	0.61	0.73	0.52	526.00
GOBP_REGULATION_OF_PROTEIN_BINDING	15.00	-0.26	-0.87	0.61	0.73	0.52	351.00
GOBP_SYNAPSE_ORGANIZATION	30.00	-0.21	-0.87	0.62	0.74	0.53	422.00
GOBP_DICARBOXYLIC_ACID_TRANSPORT	10.00	-0.29	-0.87	0.63	0.74	0.53	226.00
GOBP_ORGANIC_HYDROXY_COMPOUND_BIOSYNTHETIC_PROCESS	26.00	-0.22	-0.87	0.63	0.74	0.53	422.00
GOBP_AMINE_METABOLIC_PROCESS	17.00	-0.25	-0.87	0.63	0.74	0.53	379.00
GOBP_LIPID_MODIFICATION	14.00	-0.26	-0.86	0.61	0.73	0.52	530.00
GOBP_INTRACELLULAR_RECEPTOR_SIGNALING_PATHWAY	16.00	-0.25	-0.86	0.63	0.74	0.53	702.00
GOBP_NEGATIVE_REGULATION_OF_PHOSPHORYLATION	31.00	-0.20	-0.86	0.61	0.73	0.53	537.00
GOBP_CELLULAR_MODIFIED_AMINO_ACID_METABOLIC_PROCESS	11.00	-0.28	-0.86	0.61	0.73	0.53	530.00
GOBP_CANONICAL_WNT_SIGNALING_PATHWAY	27.00	-0.21	-0.86	0.63	0.75	0.54	273.00
GOBP_COVALENT_CHROMATIN_MODIFICATION	19.00	-0.24	-0.85	0.66	0.76	0.55	3.00
GOBP_RESPONSE_TO_CATECHOLAMINE	10.00	-0.29	-0.85	0.65	0.76	0.55	416.00
GOBP_NEGATIVE_REGULATION_OF_MITOTIC_CELL_CYCLE	26.00	-0.21	-0.85	0.65	0.76	0.55	67.00
GOBP_IMPORT_INTO_CELL	26.00	-0.21	-0.85	0.65	0.76	0.55	592.00
GOBP_HOMOTYPIC_CELL_CELL_ADHESION	12.00	-0.27	-0.85	0.64	0.75	0.54	496.00
GOBP_RESPIRATORY_GASEOUS_EXCHANGE_BY_RESPIRATORY_SYSTEM	10.00	-0.29	-0.84	0.65	0.76	0.55	348.00
GOBP_OLEFINIC_COMPOUND_METABOLIC_PROCESS	13.00	-0.26	-0.84	0.66	0.76	0.55	422.00

GOBP_LIPID_STORAGE	11.00	-0.28	-0.84	0.64	0.76	0.54	614.00
GOBP_RESPONSE_TO_ALKALOID	12.00	-0.27	-0.84	0.65	0.76	0.55	224.00
GOBP_CELL_CELL_SIGNALING_BY_WNT	39.00	-0.19	-0.84	0.69	0.79	0.56	273.00
GOBP_DETECTION_OF_STIMULUS	34.00	-0.19	-0.84	0.67	0.78	0.56	275.00
GOBP_RESPONSE_TO_TOPOLOGICALLY_INCORRECT_PROTEIN	20.00	-0.23	-0.83	0.67	0.77	0.55	174.00
GOBP_KIDNEY_EPITHELIUM_DEVELOPMENT	15.00	-0.25	-0.83	0.65	0.76	0.55	250.00
GOBP_REGULATION_OF_CELL_PROJECTION_ASSEMBLY	20.00	-0.23	-0.83	0.67	0.77	0.56	403.00
GOBP_REGULATION_OF_T_CELL_MEDIATED_IMMUNITY	12.00	-0.27	-0.83	0.65	0.76	0.55	547.00
GOBP_ENDOMEMBRANE_SYSTEM_ORGANIZATION	39.00	-0.19	-0.83	0.70	0.79	0.57	789.00
GOBP_POSITIVE_REGULATION_OF_DNA_METABOLIC_PROCESS	17.00	-0.24	-0.83	0.67	0.77	0.55	3.00
GOBP_CELLULAR_RESPONSE_TO_CALCIUM_ION	12.00	-0.26	-0.83	0.66	0.76	0.55	381.00
GOBP_SIGNAL_TRANSDUCTION_IN_RESPONSE_TO_DNA_DAMAGE	12.00	-0.26	-0.83	0.66	0.76	0.55	135.00
GOBP_POSITIVE_REGULATION_OF_CATABOLIC_PROCESS	40.00	-0.18	-0.83	0.71	0.80	0.57	586.00
GOBP_NEGATIVE_REGULATION_OF_CELL_DEVELOPMENT	12.00	-0.26	-0.82	0.67	0.77	0.55	4.00
GOBP_NIK_NF_KAPPAB_SIGNALING	19.00	-0.23	-0.82	0.69	0.79	0.57	645.00
GOBP_ORGANIC_ACID_METABOLIC_PROCESS	77.00	-0.15	-0.82	0.74	0.82	0.59	259.00
GOBP_AXON_EXTENSION	13.00	-0.25	-0.81	0.69	0.79	0.56	501.00
GOBP_NEGATIVE_REGULATION_OF_BINDING	14.00	-0.25	-0.81	0.69	0.78	0.56	286.00
GOBP_PROTEIN_LOCALIZATION_TO_CELL_PERIPHERY	30.00	-0.20	-0.81	0.69	0.79	0.57	617.00
GOBP_NEGATIVE_REGULATION_OF_CELL_PROJECTION_ORGANIZATION	16.00	-0.24	-0.81	0.69	0.79	0.57	416.00
GOBP_SMALL_MOLECULE_BIOSYNTHETIC_PROCESS	45.00	-0.17	-0.81	0.73	0.82	0.59	422.00
GOBP_POST_TRANSLATIONAL_PROTEIN_MODIFICATION	45.00	-0.17	-0.81	0.74	0.82	0.59	939.00
GOBP_CELLULAR_GLUCAN_METABOLIC_PROCESS	11.00	-0.27	-0.80	0.71	0.80	0.57	348.00
GOBP_REGULATION_OF_PROTEIN_LOCALIZATION_TO_PLASMA_MEMBRANE	12.00	-0.26	-0.80	0.68	0.78	0.56	651.00
GOBP_MUSCLE_CELL_DEVELOPMENT	14.00	-0.24	-0.80	0.71	0.80	0.57	225.00
GOBP_VESICLE_ORGANIZATION	29.00	-0.20	-0.80	0.71	0.80	0.58	568.00
GOBP_NUCLEOBASE_CONTAINING_COMPOUND_TRANSPORT	15.00	-0.23	-0.79	0.71	0.80	0.58	207.00
GOBP_GLYCEROLIPID_METABOLIC_PROCESS	32.00	-0.19	-0.79	0.72	0.80	0.58	507.00
GOBP_RESPONSE_TO_ENDOPLASMIC_RETICULUM_STRESS	23.00	-0.21	-0.79	0.71	0.80	0.57	63.00
GOBP_ESTABLISHMENT_OF_ORGANELLE_LOCALIZATION	43.00	-0.17	-0.79	0.76	0.84	0.60	587.00
GOBP_REGULATION_OF_MICROTUBULE_BASED_PROCESS	10.00	-0.27	-0.79	0.71	0.80	0.58	139.00
GOBP_NEGATIVE_REGULATION_OF_CELL_CYCLE_PHASE_TRANSITION	21.00	-0.21	-0.79	0.72	0.81	0.58	46.00
GOBP_POLYSACCHARIDE_METABOLIC_PROCESS	16.00	-0.23	-0.79	0.72	0.81	0.58	417.00
GOBP_SEXUAL_REPRODUCTION	53.00	-0.16	-0.78	0.81	0.88	0.63	243.00
GOBP_CELLULAR_AMINO_ACID_METABOLIC_PROCESS	26.00	-0.19	-0.78	0.76	0.83	0.60	154.00
GOBP_REGULATION_OF_OSTEOBLAST_DIFFERENTIATION	16.00	-0.22	-0.77	0.74	0.83	0.59	357.00
GOBP_ORGANIC_CYCLIC_COMPOUND_CATABOLIC_PROCESS	41.00	-0.17	-0.77	0.79	0.86	0.62	1137.00
GOBP_ORGANONITROGEN_COMPOUND_BIOSYNTHETIC_PROCESS	132.00	-0.13	-0.77	0.82	0.89	0.64	176.00
GOBP_DETECTION_OF_STIMULUS_INVOLVED_IN_SENSORY_PERCEPTION	20.00	-0.21	-0.77	0.75	0.83	0.60	234.00

GOBP_ESTABLISHMENT_OF_PROTEIN_LOCALIZATION_TO_MEMBRANE	28.00	-0.19	-0.77	0.76	0.83	0.60	286.00
GOBP_CELLULAR_RESPONSE_TO_INORGANIC_SUBSTANCE	21.00	-0.21	-0.77	0.76	0.83	0.60	381.00
GOBP_REGULATION_OF_PROTEIN_LOCALIZATION_TO_MEMBRANE	18.00	-0.21	-0.77	0.74	0.82	0.59	388.00
GOBP_POSITIVE_REGULATION_OF_ESTABLISHMENT_OF_PROTEIN_LOCALIZATION	27.00	-0.19	-0.76	0.77	0.84	0.61	526.00
GOBP_REGULATION_OF_POTASSIUM_ION_TRANSPORT	16.00	-0.22	-0.76	0.75	0.83	0.60	416.00
GOBP_MONOCARBOXYLIC_ACID_METABOLIC_PROCESS	47.00	-0.16	-0.76	0.81	0.88	0.63	449.00
GOBP_PROTEIN_MODIFICATION_BY_SMALL_PROTEIN_REMOVAL	31.00	-0.18	-0.76	0.74	0.82	0.59	966.00
GOBP_GAMETE_GENERATION	41.00	-0.17	-0.76	0.80	0.87	0.62	204.00
GOBP_ESTABLISHMENT_OF_CELL_POLARITY	14.00	-0.23	-0.75	0.76	0.83	0.60	425.00
GOBP_MAINTENANCE_OF_CELL_NUMBER	11.00	-0.25	-0.75	0.76	0.83	0.60	1028.00
GOBP_POSITIVE_REGULATION_OF_TRANSPORTER_ACTIVITY	11.00	-0.25	-0.75	0.76	0.83	0.60	239.00
GOBP_STRESS_ACTIVATED_PROTEIN_KINASE_SIGNALING_CASCADE	23.00	-0.19	-0.74	0.77	0.84	0.61	700.00
GOBP_ESTABLISHMENT_OF_PROTEIN_LOCALIZATION	151.00	-0.12	-0.74	0.88	0.92	0.66	69.00
GOBP_NEGATIVE_REGULATION_OF_BIOSYNTHETIC_PROCESS	118.00	-0.13	-0.74	0.84	0.90	0.65	529.00
GOBP_PEPTIDE_METABOLIC_PROCESS	60.00	-0.15	-0.74	0.82	0.88	0.63	1144.00
GOBP_AMINOGLYCAN_BIOSYNTHETIC_PROCESS	14.00	-0.22	-0.74	0.78	0.85	0.61	143.00
GOBP_REGULATION_OF_CELLULAR_AMINE_METABOLIC_PROCESS	10.00	-0.25	-0.73	0.78	0.85	0.61	163.00
GOBP_NEGATIVE_REGULATION_OF_KINASE_ACTIVITY	18.00	-0.20	-0.73	0.78	0.85	0.61	272.00
GOBP_SULFUR_COMPOUND_METABOLIC_PROCESS	24.00	-0.19	-0.73	0.80	0.87	0.62	147.00
GOBP_POSITIVE_REGULATION_OF_CELLULAR_PROTEIN_LOCALIZATION	31.00	-0.17	-0.72	0.79	0.86	0.62	667.00
GOBP_PROTEIN_LOCALIZATION_TO_MEMBRANE	58.00	-0.14	-0.72	0.85	0.91	0.65	651.00
GOBP_PROCESS_UTILIZING_AUTOPHAGIC_MECHANISM	57.00	-0.14	-0.71	0.87	0.92	0.66	651.00
GOBP_RNA_LOCALIZATION	17.00	-0.20	-0.70	0.81	0.87	0.63	1001.00
GOBP_NEGATIVE_REGULATION_OF_NEURON_PROJECTION_DEVELOPMENT	10.00	-0.23	-0.70	0.82	0.88	0.63	416.00
GOBP_RESPONSE_TO_RADIATION	41.00	-0.15	-0.69	0.87	0.92	0.66	199.00
GOBP_CELLULAR_RESPONSE_TO_PEPTIDE_HORMONE_STIMULUS	22.00	-0.18	-0.69	0.84	0.90	0.64	78.00
GOBP_PROTEIN_CONTAINING_COMPLEX_DISASSEMBLY	23.00	-0.18	-0.68	0.85	0.90	0.65	1125.00
GOBP_JNK_CASCADE	16.00	-0.20	-0.68	0.84	0.90	0.64	700.00
GOBP_SPHINGOLIPID_METABOLIC_PROCESS	17.00	-0.19	-0.68	0.83	0.89	0.64	286.00
GOBP_POSITIVE_REGULATION_OF_RESPONSE_TO_BIOTIC_STIMULUS	32.00	-0.16	-0.67	0.87	0.92	0.66	714.00
GOBP_PROTEIN_STABILIZATION	16.00	-0.19	-0.67	0.85	0.90	0.65	825.00
GOBP_ICOSANOID_METABOLIC_PROCESS	13.00	-0.21	-0.67	0.86	0.92	0.66	449.00
GOBP_SPHINGOLIPID_BIOSYNTHETIC_PROCESS	11.00	-0.22	-0.66	0.86	0.91	0.66	97.00
GOBP_POSITIVE_REGULATION_OF_CELL_GROWTH	13.00	-0.20	-0.66	0.87	0.92	0.66	402.00
GOBP_POSITIVE_REGULATION_OF_AUTOPHAGY	15.00	-0.19	-0.66	0.87	0.92	0.66	571.00
GOBP_POLYOL_METABOLIC_PROCESS	12.00	-0.21	-0.66	0.87	0.92	0.66	487.00
GOBP_ELECTRON_TRANSPORT_CHAIN	10.00	-0.22	-0.66	0.86	0.91	0.65	163.00
GOBP_PEPTIDE_BIOSYNTHETIC_PROCESS	47.00	-0.14	-0.65	0.91	0.95	0.68	1144.00
GOBP_REGULATION_OF_SIGNAL_TRANSDUCTION_BY_P53_CLASS_MEDIATOR	14.00	-0.20	-0.65	0.88	0.92	0.66	215.00

GOBP_CELLULAR_CARBOHYDRATE_METABOLIC_PROCESS	28.00	-0.16	-0.65	0.89	0.93	0.67	348.00
GOBP_REGULATION_OF_EXTENT_OF_CELL_GROWTH	11.00	-0.21	-0.65	0.88	0.92	0.66	1073.00
GOBP_GLYCEROPHOSPHOLIPID_METABOLIC_PROCESS	25.00	-0.16	-0.64	0.89	0.93	0.67	573.00
GOBP_GLYCOSYLATION	25.00	-0.16	-0.64	0.89	0.93	0.67	154.00
GOBP_NEGATIVE_REGULATION_OF_NUCLEOBASE_CONTAINING_COMPOUND_METABOLIC_PROCESS	106.00	-0.11	-0.64	0.94	0.96	0.69	529.00
GOBP_REGULATION_OF_SMALL_MOLECULE_METABOLIC_PROCESS	38.00	-0.14	-0.64	0.90	0.94	0.67	311.00
GOBP_FATTY_ACID_METABOLIC_PROCESS	27.00	-0.16	-0.63	0.90	0.94	0.67	530.00
GOBP_ORGANONITROGEN_COMPOUND_CATABOLIC_PROCESS	97.00	-0.11	-0.63	0.95	0.97	0.69	211.00
GOBP_POSITIVE_REGULATION_OF_CANONICAL_WNT_SIGNALING_PATHWAY	15.00	-0.18	-0.62	0.90	0.94	0.67	15.00
GOBP_AMIDE_BIOSYNTHETIC_PROCESS	61.00	-0.12	-0.62	0.94	0.96	0.69	176.00
GOBP_PROTEIN_LOCALIZATION_TO_CELL_JUNCTION	13.00	-0.19	-0.62	0.90	0.94	0.67	650.00
GOBP_ANTIGEN_PROCESSING_AND_PRESENTATION_OF_PEPTIDE_OR_POLYSACCHARIDE_ANTIGEN_VIA_MHC_CLASS_II	10.00	-0.21	-0.62	0.89	0.93	0.67	174.00
GOBP_DENDRITE_MORPHOGENESIS	13.00	-0.19	-0.62	0.90	0.94	0.67	409.00
GOBP_CELLULAR_PROTEIN_COMPLEX_DISASSEMBLY	17.00	-0.18	-0.62	0.88	0.93	0.67	1125.00
GOBP_REGULATION_OF_CELLULAR_CARBOHYDRATE_METABOLIC_PROCESS	10.00	-0.21	-0.62	0.89	0.93	0.67	311.00
GOBP_ATP_METABOLIC_PROCESS	18.00	-0.17	-0.61	0.89	0.93	0.67	370.00
GOBP_ESTABLISHMENT_OF_RNA_LOCALIZATION	13.00	-0.19	-0.61	0.91	0.94	0.68	1109.00
GOBP_NEGATIVE_REGULATION_OF_PROTEIN_MODIFICATION_PROCESS	40.00	-0.13	-0.60	0.93	0.95	0.68	537.00
GOBP_NEGATIVE_REGULATION_OF_DEVELOPMENTAL_GROWTH	10.00	-0.20	-0.60	0.91	0.94	0.68	184.00
GOBP_CARDIOCYTE_DIFFERENTIATION	13.00	-0.19	-0.60	0.92	0.95	0.68	534.00
GOBP_REGULATION_OF_MACROAUTOPHAGY	17.00	-0.17	-0.60	0.90	0.94	0.67	92.00
GOBP_PROTEIN_PHOSPHOPANTETHEINYLATION	24.00	-0.15	-0.60	0.93	0.96	0.69	1087.00
GOBP_CELL_PROJECTION_ASSEMBLY	47.00	-0.13	-0.59	0.94	0.96	0.69	416.00
GOBP_ENERGY_RESERVE_METABOLIC_PROCESS	13.00	-0.18	-0.59	0.92	0.95	0.68	348.00
GOBP_CELLULAR_LIPID_CATABOLIC_PROCESS	13.00	-0.18	-0.59	0.93	0.95	0.69	369.00
GOBP_LIPID_CATABOLIC_PROCESS	18.00	-0.17	-0.59	0.91	0.94	0.68	530.00
GOBP_REGULATION_OF_DNA_TEMPLATED_TRANSCRIPTION_IN_RESPONSE_TO_STRESS	11.00	-0.19	-0.59	0.92	0.95	0.68	316.00
GOBP_ENDOPLASMIC_RETICULUM_UNFOLDED_PROTEIN_RESPONSE	11.00	-0.19	-0.59	0.92	0.95	0.68	523.00
GOBP_METAL_ION_EXPORT	11.00	-0.19	-0.58	0.92	0.95	0.68	375.00
GOBP_CYTOSKELETON_DEPENDENT_INTRACELLULAR_TRANSPORT	21.00	-0.16	-0.58	0.94	0.96	0.69	568.00
GOBP_NEGATIVE_REGULATION_OF_TRANSFERASE_ACTIVITY	21.00	-0.15	-0.57	0.95	0.97	0.69	9.00
GOBP_POSITIVE_REGULATION_OF_PROTEIN_CATABOLIC_PROCESS	20.00	-0.15	-0.57	0.93	0.96	0.69	372.00
GOBP_SMALL_MOLECULE_METABOLIC_PROCESS	142.00	-0.10	-0.57	0.98	0.99	0.71	259.00
GOBP_PROTEIN_ACYLATION	10.00	-0.19	-0.57	0.94	0.96	0.69	920.00
GOBP_NEGATIVE_REGULATION_OF_PROTEIN_CONTAINING_COMPLEX_ASSEMBLY	12.00	-0.18	-0.56	0.94	0.96	0.69	139.00
GOBP_NUCLEIC_ACID_PHOSPHODIESTER_BOND_HYDROLYSIS	18.00	-0.15	-0.55	0.94	0.96	0.69	1155.00
GOBP_POSITIVE_REGULATION_OF_WNT_SIGNALING_PATHWAY	18.00	-0.15	-0.55	0.94	0.96	0.69	273.00
GOBP_REGULATION_OF_CELLULAR_LOCALIZATION	79.00	-0.10	-0.55	0.97	0.98	0.70	553.00
GOBP_RESPONSE_TO_IONIZING_RADIATION	14.00	-0.16	-0.54	0.96	0.98	0.70	372.00

GOBP_ANTIGEN_PROCESSING_AND_PRESENTATION_OF_PEPTIDE_ANTIGEN	21.00	-0.14	-0.52	0.97	0.98	0.70	952.00
GOBP_PHOSPHOLIPID_BIOSYNTHETIC_PROCESS	24.00	-0.13	-0.51	0.97	0.98	0.71	573.00
GOBP_DETECTION_OF_CHEMICAL_STIMULUS	17.00	-0.14	-0.51	0.96	0.98	0.70	1168.00
GOBP_REGULATION_OF_CELL_SIZE	19.00	-0.14	-0.51	0.97	0.98	0.70	214.00
GOBP_REGULATION_OF_CELLULAR_CATABOLIC_PROCESS	71.00	-0.10	-0.50	0.99	0.99	0.71	586.00
GOBP_REGULATION_OF_CATABOLIC_PROCESS	84.00	-0.09	-0.50	0.99	0.99	0.71	416.00
GOBP_CELLULAR_AMIDE_METABOLIC_PROCESS	84.00	-0.09	-0.50	0.99	0.99	0.71	313.00
GOBP_CELLULAR_MACROMOLECULE_LOCALIZATION	152.00	-0.08	-0.49	1.00	1.00	0.72	667.00
GOBP_CELLULAR_RESPONSE_TO_TOPOLOGICALLY_INCORRECT_PROTEIN	13.00	-0.15	-0.47	0.99	0.99	0.71	1165.00
GOBP_PROTEIN_CONTAINING_COMPLEX_SUBUNIT_ORGANIZATION	134.00	-0.08	-0.47	1.00	1.00	0.72	296.00
GOBP_MACROAUTOPHAGY	31.00	-0.10	-0.43	0.99	1.00	0.72	963.00
GOBP_MACROMOLECULE_CATABOLIC_PROCESS	101.00	-0.07	-0.41	1.00	1.00	0.72	69.00
GOBP_ORGANELLE_LOCALIZATION	59.00	0.08	0.50	0.99	1.00	0.72	159.00
GOBP_PROTEIN_LOCALIZATION_TO_ORGANELLE	89.00	0.08	0.52	1.00	1.00	0.72	968.00
GOBP_PHOSPHOLIPID_METABOLIC_PROCESS	34.00	0.10	0.55	0.97	0.98	0.70	1089.00
GOBP_PIGMENTATION	13.00	0.15	0.57	0.97	0.98	0.70	29.00
GOBP_INTRACELLULAR_TRANSPORT	123.00	0.08	0.58	0.99	1.00	0.72	989.00
GOBP_REGULATION_OF_AUTOPHAGY	35.00	0.11	0.59	0.94	0.96	0.69	43.00
GOBP_PROTEIN_CATABOLIC_PROCESS	73.00	0.09	0.60	0.97	0.98	0.70	158.00
GOBP_DENDRITE_DEVELOPMENT	18.00	0.14	0.60	0.94	0.96	0.69	125.00
GOBP_POSITIVE_REGULATION_OF_DNA_BIOSYNTHETIC_PROCESS	10.00	0.19	0.63	0.91	0.94	0.68	265.00
GOBP_GOLGI_VESICLE_TRANSPORT	26.00	0.14	0.65	0.92	0.95	0.68	889.00
GOBP_CERAMIDE_METABOLIC_PROCESS	10.00	0.20	0.65	0.89	0.93	0.67	95.00
GOBP_CARBOHYDRATE_BIOSYNTHETIC_PROCESS	13.00	0.18	0.66	0.89	0.93	0.67	1123.00
GOBP_CELLULAR_MONOVALENT_INORGANIC_CATION_HOMEOSTASIS	16.00	0.17	0.67	0.90	0.94	0.67	202.00
GOBP_FAT_CELL_DIFFERENTIATION	12.00	0.18	0.67	0.87	0.92	0.66	16.00
GOBP_CELLULAR_RESPONSE_TO_INSULIN_STIMULUS	15.00	0.17	0.68	0.90	0.94	0.67	202.00
GOBP_REGULATION_OF_GENERATION_OF_PRECURSOR_METABOLITES_AND_ENERGY	12.00	0.19	0.70	0.84	0.90	0.64	1103.00
GOBP_IRE1_MEDIATED_UNFOLDED_PROTEIN_RESPONSE	10.00	0.21	0.70	0.84	0.90	0.65	1076.00
GOBP_POSITIVE_REGULATION_OF_INTRACELLULAR_PROTEIN_TRANSPORT	16.00	0.17	0.71	0.87	0.92	0.66	983.00
GOBP_REGULATION_OF_CARBOHYDRATE_METABOLIC_PROCESS	16.00	0.18	0.71	0.86	0.91	0.66	1123.00
GOBP_AEROBIC_RESPIRATION	10.00	0.21	0.72	0.83	0.89	0.64	1070.00
GOBP_POSITIVE_REGULATION_OF_BINDING	14.00	0.19	0.74	0.80	0.87	0.62	255.00
GOBP_PROTEIN_LOCALIZATION_TO_NUCLEUS	26.00	0.15	0.74	0.84	0.90	0.65	69.00
GOBP_LONG_CHAIN_FATTY_ACID_METABOLIC_PROCESS	11.00	0.21	0.74	0.82	0.88	0.63	156.00
GOBP_PURINE_CONTAINING_COMPOUND_BIOSYNTHETIC_PROCESS	12.00	0.20	0.74	0.78	0.85	0.61	154.00
GOBP_ACTIVATION_OF_CYSTEINE_TYPE_ENDOPEPTIDASE_ACTIVITY_INVOLVED_IN_APOPTOTIC_PROCESS	11.00	0.21	0.74	0.82	0.88	0.63	1076.00
GOBP_PURINE_CONTAINING_COMPOUND_METABOLIC_PROCESS	29.00	0.15	0.75	0.82	0.88	0.63	253.00
GOBP_MONOVALENT_INORGANIC_CATION_HOMEOSTASIS	19.00	0.18	0.75	0.78	0.85	0.61	202.00
GOBP_RIBOSE_PHOSPHATE_BIOSYNTHETIC_PROCESS	11.00	0.22	0.77	0.78	0.85	0.61	154.00

GOBP_REGULATION_OF_NUCLEOCYTOPLASMIC_TRANSPORT	10.00	0.23	0.77	0.75	0.83	0.60	411.00
GOBP_EMBRYONIC_SKELETAL_SYSTEM_DEVELOPMENT	10.00	0.23	0.78	0.74	0.82	0.59	74.00
GOBP_PROTEIN_TARGETING	36.00	0.14	0.78	0.76	0.84	0.60	868.00
GOBP_CELLULAR_PROTEIN_CONTAINING_COMPLEX_ASSEMBLY	72.00	0.12	0.78	0.83	0.89	0.64	479.00
GOBP_MITOCHONDRIAL_MEMBRANE_ORGANIZATION	12.00	0.21	0.78	0.71	0.80	0.58	606.00
GOBP_ORGANOPHOSPHATE_BIOSYNTHETIC_PROCESS	43.00	0.14	0.78	0.78	0.85	0.61	226.00
GOBP_MONOSACCHARIDE_METABOLIC_PROCESS	17.00	0.19	0.79	0.76	0.84	0.60	1105.00
GOBP_SPECIFICATION_OF_SYMMETRY	12.00	0.22	0.79	0.70	0.79	0.57	1070.00
GOBP_REGULATION_OF_PH	11.00	0.23	0.79	0.75	0.83	0.59	1056.00
GOBP_VESICLE_LOCALIZATION	23.00	0.17	0.80	0.70	0.80	0.57	112.00
GOBP_POSITIVE_REGULATION_OF_CELLULAR_PROTEIN_CATABOLIC_PROCESS	13.00	0.21	0.81	0.70	0.79	0.57	84.00
GOBP_SMALL_MOLECULE_CATABOLIC_PROCESS	29.00	0.16	0.81	0.74	0.82	0.59	245.00
GOBP_REGULATION_OF_PROTEIN_STABILITY	20.00	0.19	0.82	0.70	0.79	0.57	986.00
GOBP_MALE_SEX_DIFFERENTIATION	18.00	0.20	0.82	0.73	0.82	0.59	28.00
GOBP_MALE_GAMETE_GENERATION	27.00	0.17	0.82	0.71	0.80	0.57	185.00
GOBP_CELLULAR_LIPID_METABOLIC_PROCESS	75.00	0.13	0.82	0.77	0.84	0.60	205.00
GOBP_MEMBRANE_DOCKING	14.00	0.22	0.82	0.70	0.80	0.57	581.00
GOBP_MEMBRANE_LIPID_METABOLIC_PROCESS	20.00	0.19	0.83	0.69	0.79	0.57	154.00
GOBP_SENSORY_PERCEPTION_OF_SMELL	14.00	0.22	0.83	0.69	0.79	0.57	478.00
GOBP_STRIATED_MUSCLE_CELL_DEVELOPMENT	10.00	0.25	0.84	0.65	0.76	0.55	85.00
GOBP_POTASSIUM_ION_TRANSPORT	27.00	0.17	0.84	0.69	0.79	0.57	251.00
GOBP_ORGANOPHOSPHATE_CATABOLIC_PROCESS	12.00	0.23	0.84	0.64	0.76	0.54	244.00
GOBP_ENDOSOMAL_TRANSPORT	20.00	0.19	0.84	0.68	0.78	0.56	43.00
GOBP_POSITIVE_REGULATION_OF_INTRACELLULAR_TRANSPORT	20.00	0.19	0.85	0.68	0.78	0.56	983.00
GOBP_DNA_TEMPLATED_TRANSCRIPTION_INITIATION	13.00	0.23	0.85	0.66	0.76	0.55	902.00
GOBP_CARBOHYDRATE_DERIVATIVE_BIOSYNTHETIC_PROCESS	54.00	0.14	0.86	0.72	0.80	0.58	197.00
GOBP_REGULATION_OF_CELLULAR_RESPONSE_TO_TRANSFORMING_GROWTH_FACTOR_BETA_STIMULUS	16.00	0.21	0.86	0.65	0.76	0.55	99.00
GOBP_CARDIAC_MUSCLE_CELL_DIFFERENTIATION	10.00	0.26	0.86	0.61	0.73	0.53	108.00
GOBP_INTRACELLULAR_PROTEIN_TRANSPORT	85.00	0.13	0.86	0.70	0.79	0.57	983.00
GOBP_MEMBRANE_LIPID_BIOSYNTHETIC_PROCESS	14.00	0.23	0.87	0.62	0.74	0.53	154.00
GOBP_ORGANIC_ACID_CATABOLIC_PROCESS	14.00	0.23	0.87	0.62	0.74	0.53	838.00
GOBP_REGULATION_OF_CELLULAR_PROTEIN_LOCALIZATION	52.00	0.14	0.87	0.67	0.77	0.55	267.00
GOBP_REGULATION_OF_INTRACELLULAR_TRANSPORT	33.00	0.17	0.88	0.63	0.75	0.54	267.00
GOBP_HINDBRAIN_DEVELOPMENT	12.00	0.24	0.89	0.57	0.71	0.51	41.00
GOBP_PROTEIN_AUTOPHOSPHORYLATION	29.00	0.18	0.89	0.63	0.74	0.53	121.00
GOBP_CALCIUM_MEDIATED_SIGNALING	18.00	0.21	0.89	0.65	0.76	0.54	43.00
GOBP_CELLULAR_MACROMOLECULE_CATABOLIC_PROCESS	80.00	0.14	0.90	0.62	0.74	0.53	1186.00
GOBP_GLUCOSE_METABOLIC_PROCESS	12.00	0.24	0.90	0.56	0.69	0.50	442.00
GOBP_NUCLEOBASE_CONTAINING_SMALL_MOLECULE_METABOLIC_PROCESS	38.00	0.16	0.90	0.62	0.73	0.53	253.00
GOBP_ESTABLISHMENT_OF_PROTEIN_LOCALIZATION_TO_ORGANELLE	54.00	0.15	0.90	0.61	0.73	0.53	966.00
GOBP_NEGATIVE_REGULATION_OF_TRANSMEMBRANE_RECEPTOR_PROTEIN_SERINE_THREONINE_KINASE_SIGNALING_PATHWAY	15.00	0.23	0.91	0.58	0.71	0.51	99.00

GOBP_UNSATURATED_FATTY_ACID_METABOLIC_PROCESS	12.00	0.25	0.91	0.53	0.67	0.48	484.00
GOBP_GENERATION_OF_PRECURSOR_METABOLITES_AND_ENERGY	40.00	0.16	0.91	0.60	0.72	0.52	411.00
GOBP_RENAL_TUBULE_DEVELOPMENT	10.00	0.27	0.91	0.55	0.68	0.49	235.00
GOBP_REGULATION_OF_CELL_CYCLE	107.00	0.13	0.92	0.67	0.77	0.55	483.00
GOBP_REGULATION_OF_DNA_BIOSYNTHETIC_PROCESS	13.00	0.24	0.92	0.53	0.67	0.48	281.00
GOBP_ENERGY_DERIVATION_BY_OXIDATION_OF_ORGANIC_COMPOUNDS	26.00	0.19	0.92	0.59	0.71	0.51	441.00
GOBP_REGULATION_OF_PROTEIN_LOCALIZATION_TO_NUCLEUS	14.00	0.24	0.92	0.54	0.68	0.49	132.00
GOBP_NEGATIVE_REGULATION_OF_CELLULAR_COMPONENT_ORGANIZATION	62.00	0.15	0.93	0.64	0.76	0.54	305.00
GOBP_LYSOSOMAL_TRANSPORT	11.00	0.26	0.93	0.52	0.66	0.47	1004.00
GOBP_ORGANOPHOSPHATE_METABOLIC_PROCESS	71.00	0.14	0.93	0.60	0.72	0.52	270.00
GOBP_REGULATION_OF_RESPONSE_TO_BIOTIC_STIMULUS	47.00	0.16	0.93	0.58	0.71	0.51	30.00
GOBP_PROTEIN_LOCALIZATION_TO_MITOCHONDRION	18.00	0.22	0.93	0.57	0.71	0.51	606.00
GOBP_METENCEPHALON_DEVELOPMENT	10.00	0.28	0.94	0.52	0.66	0.48	41.00
GOBP_SENSORY_PERCEPTION_OF_CHEMICAL_STIMULUS	15.00	0.24	0.95	0.51	0.65	0.47	478.00
GOBP_NUCLEAR_EXPORT	10.00	0.28	0.95	0.50	0.64	0.46	579.00
GOBP_ORGANELLE_ASSEMBLY	78.00	0.15	0.96	0.52	0.66	0.47	347.00
GOBP_FEMALE_GAMETE_GENERATION	10.00	0.29	0.97	0.48	0.62	0.45	157.00
GOBP_POSITIVE_REGULATION_OF_PROTEIN_LOCALIZATION_TO_NUCLEUS	10.00	0.29	0.97	0.47	0.62	0.44	968.00
GOBP_ISOPRENOID_METABOLIC_PROCESS	11.00	0.28	0.97	0.46	0.61	0.43	556.00
GOBP_NEGATIVE_REGULATION_OF_AUTOPHAGY	13.00	0.26	0.97	0.45	0.60	0.43	43.00
GOBP_NEGATIVE_REGULATION_OF_ESTABLISHMENT_OF_PROTEIN_LOCALIZATION	11.00	0.28	0.97	0.45	0.60	0.43	43.00
GOBP_NCRNA_TRANSCRIPTION	10.00	0.29	0.98	0.46	0.61	0.44	735.00
GOBP_NOTCH_SIGNALING_PATHWAY	24.00	0.21	0.98	0.50	0.64	0.46	74.00
GOBP_MICROTUBULE_BASED_TRANSPORT	15.00	0.25	0.98	0.46	0.61	0.44	889.00
GOBP_TRANSPORT_ALONG_MICROTUBULE	15.00	0.25	0.98	0.46	0.61	0.44	889.00
GOBP_PROTON_TRANSMEMBRANE_TRANSPORT	11.00	0.28	0.99	0.44	0.59	0.43	359.00
GOBP_POSITIVE_REGULATION_OF_CELL_KILLING	13.00	0.26	0.99	0.44	0.59	0.43	1.00
GOBP_POSITIVE_REGULATION_OF_CELL_CYCLE	33.00	0.19	0.99	0.50	0.64	0.46	185.00
GOBP_POSTSYNAPSE_ORGANIZATION	12.00	0.27	0.99	0.42	0.58	0.41	125.00
GOBP_REGULATION_OF_PROTEIN_CATABOLIC_PROCESS	32.00	0.19	0.99	0.48	0.63	0.45	158.00
GOBP_NUCLEUS_ORGANIZATION	11.00	0.28	0.99	0.43	0.59	0.42	979.00
GOBP_POSITIVE_REGULATION_OF_PROTEOLYSIS_INVOLVED_IN_CELLULAR_PROTEIN_CATABOLIC_PROCESS	10.00	0.30	1.00	0.44	0.59	0.43	84.00
GOBP_POSITIVE_REGULATION_OF_UBIQUITIN_DEPENDENT_PROTEIN_CATABOLIC_PROCESS	10.00	0.30	1.00	0.44	0.59	0.43	84.00
GOBP_RESPONSE_TO_STARVATION	15.00	0.25	1.00	0.43	0.59	0.42	43.00
GOBP_ALCOHOL_METABOLIC_PROCESS	34.00	0.19	1.00	0.49	0.63	0.45	244.00
GOBP_ALCOHOL_BIOSYNTHETIC_PROCESS	19.00	0.23	1.00	0.44	0.59	0.43	244.00
GOBP_REGULATION_OF_MITOTIC_CELL_CYCLE	54.00	0.16	1.01	0.47	0.62	0.44	449.00
GOBP_CELLULAR_RESPIRATION	13.00	0.27	1.01	0.42	0.57	0.41	403.00
GOBP_LIPOSACCHARIDE_METABOLIC_PROCESS	11.00	0.29	1.01	0.42	0.58	0.41	150.00
GOBP_ANTERIOR_POSTERIOR_PATTERN_SPECIFICATION	13.00	0.27	1.01	0.42	0.57	0.41	74.00
GOBP_NEGATIVE_REGULATION_OF_CELL_CYCLE	58.00	0.16	1.02	0.45	0.60	0.43	185.00

GOBP_ANATOMICAL_STRUCTURE_MATURATION	18.00	0.24	1.02	0.42	0.58	0.42	216.00
GOBP_MITOCHONDRION_ORGANIZATION	50.00	0.17	1.02	0.44	0.60	0.43	440.00
GOBP_REGULATION_OF_INNATE_IMMUNE_RESPONSE	42.00	0.18	1.03	0.42	0.57	0.41	30.00
GOBP_PEPTIDYL_SERINE_MODIFICATION	32.00	0.20	1.03	0.45	0.60	0.43	104.00
GOBP_REGULATION_OF_CELLULAR_PROTEIN_CATABOLIC_PROCESS	19.00	0.24	1.03	0.40	0.57	0.41	158.00
GOBP_PROTEIN_FOLDING	15.00	0.26	1.04	0.38	0.55	0.40	1008.00
GOBP_VACUOLAR_TRANSPORT	15.00	0.26	1.05	0.37	0.54	0.39	1004.00
GOBP_MICROTUBULE_BASED_PROCESS	65.00	0.16	1.05	0.41	0.57	0.41	252.00
GOBP_SPINDLE_ORGANIZATION	18.00	0.25	1.06	0.38	0.55	0.39	347.00
GOBP_NUCLEOSIDE_PHOSPHATE_BIOSYNTHETIC_PROCESS	13.00	0.28	1.06	0.36	0.53	0.38	226.00
GOBP_MITOCHONDRIAL_TRANSPORT	22.00	0.23	1.06	0.41	0.57	0.41	533.00
GOBP_RNA_CATABOLIC_PROCESS	26.00	0.22	1.06	0.40	0.56	0.40	1065.00
GOBP_DEVELOPMENTAL_MATURATION	22.00	0.24	1.07	0.41	0.57	0.41	216.00
GOBP_CHROMATIN_ORGANIZATION	37.00	0.20	1.07	0.39	0.55	0.40	199.00
GOBP_SULFUR_COMPOUND_BIOSYNTHETIC_PROCESS	14.00	0.28	1.08	0.34	0.51	0.37	286.00
GOBP_DNA_BIOSYNTHETIC_PROCESS	19.00	0.25	1.09	0.34	0.51	0.37	299.00
GOBP_NUCLEAR_TRANSCRIBED_MRNA_CATABOLIC_PROCESS	14.00	0.29	1.10	0.33	0.50	0.36	975.00
GOBP_GENE_SILENCING	15.00	0.28	1.10	0.32	0.49	0.35	987.00
GOBP_MEMBRANE_FUSION	16.00	0.27	1.11	0.31	0.49	0.35	112.00
GOBP_CHROMATIN_ASSEMBLY_OR_DISASSEMBLY	14.00	0.29	1.11	0.31	0.49	0.35	493.00
GOBP_REGULATION_OF_PROTEIN_TARGETING	12.00	0.30	1.12	0.30	0.47	0.34	440.00
GOBP_SODIUM_ION_TRANSMEMBRANE_TRANSPORT	10.00	0.34	1.12	0.30	0.47	0.34	77.00
GOBP_PROTEIN_IMPORT	14.00	0.29	1.12	0.31	0.48	0.35	966.00
GOBP_NUCLEAR_TRANSPORT	25.00	0.24	1.13	0.33	0.50	0.36	966.00
GOBP_GLYCOSYL_COMPOUND_METABOLIC_PROCESS	11.00	0.32	1.13	0.27	0.43	0.31	928.00
GOBP_ACTIVATION_OF_INNATE_IMMUNE_RESPONSE	17.00	0.27	1.13	0.27	0.43	0.31	1.00
GOBP_STEROL_METABOLIC_PROCESS	13.00	0.30	1.14	0.26	0.43	0.31	205.00
GOBP_NEGATIVE_REGULATION_OF_TRANSFORMING_GROWTH_FACTOR_BETA_RECEPTOR_SIGNALING_PATHWAY	12.00	0.31	1.14	0.28	0.45	0.32	99.00
GOBP_MEMORY	12.00	0.31	1.15	0.27	0.44	0.31	31.00
GOBP_REGULATION_OF_CELL_KILLING	16.00	0.28	1.15	0.26	0.43	0.31	14.00
GOBP_IMPORT_INTO_NUCLEUS	15.00	0.29	1.16	0.26	0.43	0.31	966.00
GOBP_REGULATION_OF_ORGANELLE_ASSEMBLY	19.00	0.27	1.16	0.30	0.47	0.34	737.00
GOBP_POSITIVE_REGULATION_OF_MITOTIC_CELL_CYCLE	10.00	0.35	1.16	0.27	0.44	0.31	662.00
GOBP_POSITIVE_REGULATION_OF_LEUKOCYTE_MEDIATED_IMMUNITY	15.00	0.29	1.17	0.26	0.43	0.31	144.00
GOBP_CELLULAR_PROCESS_INVOLVED_IN_REPRODUCTION_IN_MULTICELLULAR_ORGANISM	17.00	0.28	1.17	0.25	0.41	0.30	175.00
GOBP_CELLULAR_RESPONSE_TO_STARVATION	11.00	0.33	1.17	0.24	0.40	0.29	43.00
GOBP_REGULATION_OF_LEUKOCYTE_MEDIATED_IMMUNITY	26.00	0.24	1.17	0.25	0.42	0.30	144.00
GOBP_INNATE_IMMUNE_RESPONSE_ACTIVATING_SIGNAL_TRANSDUCTION	14.00	0.31	1.17	0.26	0.43	0.31	1.00
GOBP_REGULATION_OF_SYNAPSE_STRUCTURE_OR_ACTIVITY	17.00	0.29	1.18	0.24	0.40	0.29	43.00
GOBP_CELL_SURFACE_RECEPTOR_SIGNALING_PATHWAY_INVOLVED_IN_CELL_CELL_SIGNALING	50.00	0.20	1.18	0.22	0.39	0.28	109.00

GOBP_REGULATION_OF_ALCOHOL_BIOSYNTHETIC_PROCESS	12.00	0.32	1.19	0.24	0.40	0.29	216.00
GOBP_MICROTUBULE_BASED_MOVEMENT	20.00	0.27	1.19	0.28	0.45	0.32	889.00
GOBP_REGULATION_OF_ORGANELLE_ORGANIZATION	113.00	0.17	1.20	0.16	0.32	0.23	250.00
GOBP_POSITIVE_REGULATION_OF_EXOCYTOSIS	14.00	0.31	1.20	0.24	0.40	0.29	128.00
GOBP_REGULATION_OF_PROTEIN_MODIFICATION_BY_SMALL_PROTEIN_CONJUGATION_OR_REMOVAL	18.00	0.29	1.20	0.24	0.40	0.29	501.00
GOBP_SPINDLE_ASSEMBLY	11.00	0.34	1.20	0.21	0.38	0.27	347.00
GOBP_FERTILIZATION	15.00	0.31	1.21	0.21	0.38	0.27	128.00
GOBP_CYTOKINESIS	17.00	0.30	1.22	0.20	0.36	0.26	244.00
GOBP_CELL_CYCLE	167.00	0.16	1.22	0.10	0.23	0.16	311.00
GOBP_NEGATIVE_REGULATION_OF_MAPK_CASCADE	12.00	0.33	1.22	0.22	0.39	0.28	475.00
GOBP_SODIUM_ION_TRANSPORT	17.00	0.30	1.22	0.19	0.36	0.26	963.00
GOBP_DNA_PACKAGING	13.00	0.33	1.22	0.22	0.39	0.28	191.00
GOBP_REGULATION_OF_MITOCHONDRION_ORGANIZATION	19.00	0.29	1.23	0.23	0.40	0.29	247.00
GOBP_MICROTUBULE_CYTOSKELETON_ORGANIZATION	46.00	0.22	1.23	0.17	0.33	0.24	242.00
GOBP_INTERLEUKIN_1_MEDIATED_SIGNALING_PATHWAY	11.00	0.35	1.23	0.18	0.35	0.25	887.00
GOBP_REGULATION_OF_ESTABLISHMENT_OF_PROTEIN_LOCALIZATION_TO_MITOCHONDRION	11.00	0.35	1.24	0.18	0.34	0.25	247.00
GOBP_CYTOSOLIC_TRANSPORT	16.00	0.31	1.25	0.18	0.34	0.25	946.00
GOBP_REGULATION_OF_INTRACELLULAR_PROTEIN_TRANSPORT	24.00	0.27	1.25	0.18	0.35	0.25	252.00
GOBP_REGULATION_OF_LEUKOCYTE_MEDIATED_CYTOTOXICITY	13.00	0.33	1.25	0.20	0.37	0.27	14.00
GOBP_CELL_DIVISION	58.00	0.20	1.25	0.16	0.33	0.23	426.00
GOBP_METANEPHROS_DEVELOPMENT	10.00	0.37	1.25	0.20	0.36	0.26	16.00
GOBP_DNA_RECOMBINATION	23.00	0.27	1.25	0.20	0.36	0.26	522.00
GOBP_RNA_3_END_PROCESSING	10.00	0.38	1.26	0.19	0.36	0.26	852.00
GOBP_CELLULAR_RESPONSE_TO_RADIATION	12.00	0.34	1.26	0.18	0.35	0.25	166.00
GOBP_COGNITION	19.00	0.30	1.27	0.20	0.37	0.26	146.00
GOBP_NEGATIVE_REGULATION_OF_ORGANELLE_ORGANIZATION	33.00	0.24	1.28	0.16	0.32	0.23	305.00
GOBP_NEGATIVE_REGULATION_OF_CELL_CYCLE_PROCESS	32.00	0.25	1.29	0.16	0.32	0.23	185.00
GOBP_POSITIVE_REGULATION_OF_REGULATED_SECRETORY_PATHWAY	11.00	0.37	1.30	0.15	0.31	0.22	128.00
GOBP_CELLULAR_PROTEIN_CATABOLIC_PROCESS	59.00	0.21	1.30	0.11	0.25	0.18	776.00
GOBP_MITOTIC_CELL_CYCLE	105.00	0.18	1.30	0.10	0.23	0.17	267.00
GOBP_REGULATION_OF_REPRODUCTIVE_PROCESS	13.00	0.35	1.31	0.16	0.33	0.23	185.00
GOBP_NERVOUS_SYSTEM_PROCESS	97.00	0.19	1.31	0.07	0.20	0.14	97.00
GOBP_NEGATIVE_REGULATION_OF_NERVOUS_SYSTEM_DEVELOPMENT	11.00	0.37	1.31	0.15	0.31	0.22	69.00
GOBP_CELL_CYCLE_PROCESS	122.00	0.18	1.32	0.07	0.19	0.13	271.00
GOBP_RESPONSE_TO_LIGHT_STIMULUS	25.00	0.28	1.33	0.15	0.31	0.22	178.00
GOBP_ORGANELLE_FUSION	10.00	0.40	1.33	0.14	0.30	0.21	112.00
GOBP_PROTEIN_CONTAINING_COMPLEX_LOCALIZATION	23.00	0.29	1.33	0.14	0.30	0.21	631.00
GOBP_CILIUM_ORGANIZATION	30.00	0.26	1.33	0.13	0.28	0.20	581.00
GOBP_REGULATION_OF_TRANS_SYNAPTIC_SIGNALING	30.00	0.27	1.34	0.13	0.27	0.20	192.00
GOBP_CELL_MATURATION	12.00	0.37	1.35	0.14	0.30	0.21	194.00
GOBP_PROTEIN_TARGETING_TO_MITOCHONDRION	10.00	0.41	1.37	0.12	0.27	0.19	533.00
GOBP_DNA_CONFORMATION_CHANGE	20.00	0.31	1.37	0.12	0.26	0.19	281.00

GOBP_REGULATION_OF_UBIQUITIN_DEPENDENT_PROTEIN_CATABOLIC_PROCESS	13.00	0.37	1.37	0.13	0.28	0.20	84.00
GOBP_GERM_CELL_DEVELOPMENT	10.00	0.41	1.38	0.12	0.26	0.19	157.00
GOBP_PROTEIN_MODIFICATION_BY_SMALL_PROTEIN_CONJUGATION_OR_REMOVAL	82.00	0.21	1.38	0.07	0.20	0.14	737.00
GOBP_CHROMATIN_REMODELING	16.00	0.34	1.38	0.12	0.26	0.19	254.00
GOBP_NATURAL_KILLER_CELL_MEDIATED_IMMUNITY	11.00	0.40	1.41	0.11	0.24	0.17	14.00
GOBP_LEARNING	12.00	0.39	1.42	0.11	0.25	0.18	97.00
GOBP_RNA_SPLICING_VIA_TRANSESTERIFICATION_REACTIONS	22.00	0.31	1.42	0.10	0.23	0.16	940.00
GOBP_VESICLE_MEDIATED_TRANSPORT_IN_SYNAPSE	19.00	0.34	1.44	0.08	0.21	0.15	248.00
GOBP_VIRAL_GENE_EXPRESSION	14.00	0.38	1.44	0.08	0.21	0.15	726.00
GOBP_CELL_CYCLE_PHASE_TRANSITION	56.00	0.23	1.45	0.06	0.18	0.13	447.00
GOBP_CELLULAR_RESPONSE_TO_DNA_DAMAGE_STIMULUS	68.00	0.22	1.45	0.04	0.13	0.10	541.00
GOBP_REGULATION_OF_PROTEASOMAL_UBIQUITIN_DEPENDENT_PROTEIN_CATABOLIC_PROCESS	12.00	0.40	1.45	0.10	0.23	0.17	84.00
GOBP_POSITIVE_REGULATION_OF_CELL_CYCLE_PROCESS	22.00	0.32	1.46	0.08	0.21	0.15	267.00
GOBP_OLIGODENDROCYTE_DIFFERENTIATION	11.00	0.41	1.46	0.09	0.22	0.16	108.00
GOBP_MICROTUBULE_CYTOSKELETON_ORGANIZATION_INVOLVED_IN_MITOSIS	18.00	0.35	1.46	0.09	0.22	0.16	238.00
GOBP_PROTEIN_MODIFICATION_BY_SMALL_PROTEIN_CONJUGATION	62.00	0.23	1.46	0.05	0.16	0.11	726.00
GOBP_CYTOSKELETON_DEPENDENT_CYTOKINESIS	12.00	0.40	1.47	0.10	0.23	0.17	396.00
GOBP_MITOTIC_CYTOKINESIS	12.00	0.40	1.47	0.10	0.23	0.17	396.00
GOBP_REGULATION_OF_NATURAL_KILLER_CELL_MEDIATED_IMMUNITY	10.00	0.44	1.47	0.08	0.20	0.14	14.00
GOBP_SYNAPTIC_SIGNALING	46.00	0.26	1.49	0.06	0.16	0.12	268.00
GOBP_REGULATION_OF_LYMPHOCYTE_MEDIATED_IMMUNITY	19.00	0.35	1.49	0.08	0.21	0.15	144.00
GOBP_TELOMERE_ORGANIZATION	12.00	0.41	1.50	0.09	0.22	0.16	587.00
GOBP_PROTEIN_POLYUBIQUITINATION	31.00	0.30	1.51	0.06	0.17	0.12	726.00
GOBP_RNA_SPLICING	26.00	0.31	1.51	0.05	0.15	0.11	940.00
GOBP_SINGLE_FERTILIZATION	12.00	0.42	1.52	0.08	0.21	0.15	128.00
GOBP_NEUROTRANSMITTER_SECRETION	12.00	0.42	1.54	0.08	0.20	0.15	183.00
GOBP_REGULATION_OF_PROTEASOMAL_PROTEIN_CATABOLIC_PROCESS	14.00	0.41	1.56	0.07	0.19	0.14	598.00
GOBP_MRNA_METABOLIC_PROCESS	51.00	0.26	1.57	0.03	0.11	0.08	975.00
GOBP_REGULATION_OF_SYNAPTIC_PLASTICITY	14.00	0.41	1.57	0.07	0.19	0.14	125.00
GOBP_TRANSCRIPTION_COUPLED_NUCLEOTIDE_EXCISION_REPAIR	10.00	0.47	1.57	0.07	0.19	0.14	726.00
GOBP_REGULATION_OF_CELL_CYCLE_PROCESS	67.00	0.24	1.58	0.01	0.07	0.05	480.00
GOBP_POSITIVE_REGULATION_OF_CHROMOSOME_ORGANIZATION	11.00	0.45	1.60	0.04	0.13	0.09	66.00
GOBP_MITOCHONDRIAL_TRANSLATIONAL_TERMINATION	11.00	0.45	1.60	0.04	0.13	0.09	586.00
GOBP_TRANSLATIONAL_ELONGATION	11.00	0.45	1.60	0.04	0.13	0.09	586.00
GOBP_TRANSLATIONAL_TERMINATION	11.00	0.45	1.60	0.04	0.13	0.09	586.00
GOBP_MRNA_PROCESSING	30.00	0.32	1.60	0.03	0.10	0.07	940.00
GOBP_MITOTIC_SPINDLE_ORGANIZATION	13.00	0.43	1.60	0.05	0.15	0.11	307.00
GOBP_MODIFICATION_DEPENDENT_MACROMOLECULE_CATABOLIC_PROCESS	50.00	0.27	1.61	0.02	0.08	0.05	776.00
GOBP_DNA_METABOLIC_PROCESS	66.00	0.25	1.61	0.01	0.06	0.04	541.00
GOBP_REGULATION_OF_MEMBRANE_POTENTIAL	38.00	0.30	1.62	0.03	0.11	0.08	137.00
GOBP_REGULATION_OF_DNA_REPLICATION	10.00	0.49	1.62	0.05	0.16	0.11	317.00

GOBP_CHROMOSOME_ORGANIZATION	75.00	0.25	1.64	0.02	0.07	0.05	503.00
GOBP_CELLULAR_DEFENSE_RESPONSE	13.00	0.44	1.65	0.04	0.13	0.09	9.00
GOBP_NUCLEOTIDE_EXCISION_REPAIR	11.00	0.47	1.65	0.03	0.10	0.07	726.00
GOBP_MACROMOLECULE_METHYLATION	13.00	0.44	1.66	0.04	0.13	0.09	766.00
GOBP_REGULATION_OF_CELL_CYCLE_PHASE_TRANSITION	39.00	0.30	1.66	0.02	0.07	0.05	447.00
GOBP_REGULATION_OF_NEUROTRANSMITTER_LEVELS	18.00	0.40	1.68	0.03	0.11	0.08	112.00
GOBP_REGULATION_OF_EXOCYTOSIS	21.00	0.38	1.69	0.02	0.08	0.06	128.00
GOBP_REGULATION_OF_REGULATED_SECRETORY_PATHWAY	18.00	0.41	1.72	0.03	0.10	0.07	128.00
GOBP_TRANSLATIONAL_INITIATION	12.00	0.47	1.73	0.04	0.13	0.09	726.00
GOBP_PROTEASOMAL_PROTEIN_CATABOLIC_PROCESS	38.00	0.32	1.75	0.01	0.06	0.05	689.00
GOBP_REGULATION_OF_MITOTIC_NUCLEAR_DIVISION	15.00	0.44	1.76	0.02	0.10	0.07	246.00
GOBP_RNA_PROCESSING	53.00	0.29	1.77	0.01	0.04	0.03	940.00
GOBP_MITOCHONDRIAL_TRANSLATION	13.00	0.47	1.78	0.02	0.08	0.06	586.00
GOBP_MITOCHONDRIAL_GENE_EXPRESSION	16.00	0.44	1.78	0.01	0.06	0.04	664.00
GOBP_NEGATIVE_REGULATION_OF_CELLULAR_PROTEIN_LOCALIZATION	11.00	0.51	1.79	0.01	0.07	0.05	250.00
GOBP_METHYLATION	16.00	0.44	1.79	0.01	0.06	0.04	766.00
GOBP_NCRNA_PROCESSING	17.00	0.44	1.81	0.02	0.07	0.05	774.00
GOBP_NEUROTRANSMITTER_TRANSPORT	17.00	0.44	1.81	0.02	0.07	0.05	112.00
GOBP_DNA_INTEGRITY_CHECKPOINT	13.00	0.49	1.85	0.01	0.06	0.04	483.00
GOBP_REGULATION_OF_NERVOUS_SYSTEM_PROCESS	14.00	0.49	1.87	0.02	0.08	0.06	87.00
GOBP_NCRNA_METABOLIC_PROCESS	25.00	0.39	1.87	0.01	0.05	0.04	836.00
GOBP_CHEMICAL_SYNAPTIC_TRANSMISSION_POSTSYNAPTIC	10.00	0.56	1.87	0.01	0.07	0.05	87.00
GOBP_REGULATION_OF_NUCLEAR_DIVISION	17.00	0.46	1.90	0.01	0.05	0.04	246.00
GOBP_DNA_REPLICATION_INDEPENDENT_NUCLEOSOME_ORGANIZATION	10.00	0.57	1.91	0.01	0.06	0.04	476.00
GOBP_NUCLEOSOME_ASSEMBLY	10.00	0.57	1.91	0.01	0.06	0.04	476.00
GOBP_NUCLEOSOME_ORGANIZATION	10.00	0.57	1.91	0.01	0.06	0.04	476.00
GOBP_RIBOSOME_BIOGENESIS	13.00	0.51	1.92	0.01	0.04	0.03	672.00
GOBP_PEPTIDYL_LYSINE_MODIFICATION	19.00	0.45	1.93	0.01	0.05	0.03	495.00
GOBP_DOUBLE_STRAND_BREAK_REPAIR	18.00	0.46	1.93	0.01	0.05	0.03	541.00
GOBP_PROTEIN_DNA_COMPLEX_SUBUNIT_ORGANIZATION	15.00	0.49	1.93	0.01	0.05	0.04	476.00
GOBP_CELL_CYCLE_CHECKPOINT	18.00	0.47	1.95	0.01	0.04	0.03	426.00
GOBP_REGULATION_OF_POSTSYNAPTIC_MEMBRANE_POTENTIAL	12.00	0.53	1.95	0.01	0.05	0.04	123.00
GOBP_REGULATION_OF_CHROMOSOME_ORGANIZATION	23.00	0.43	1.97	0.00	0.03	0.02	281.00
GOBP_MITOTIC_CELL_CYCLE_CHECKPOINT	12.00	0.55	2.04	0.00	0.03	0.02	334.00
GOBP_RIBONUCLEOPROTEIN_COMPLEX_SUBUNIT_ORGANIZATION	16.00	0.51	2.07	0.00	0.01	0.01	672.00
GOBP_DNA_REPAIR	43.00	0.37	2.08	0.00	0.01	0.01	541.00
GOBP_CELL_CYCLE_G2_M_PHASE_TRANSITION	25.00	0.45	2.14	0.00	0.01	0.01	666.00
GOBP_SISTER_CHROMATID_SEGREGATION	23.00	0.47	2.16	0.00	0.01	0.01	307.00
GOBP_MITOTIC_NUCLEAR_DIVISION	34.00	0.41	2.16	0.00	0.01	0.00	311.00
GOBP_REGULATION_OF_CELL_CYCLE_G2_M_PHASE_TRANSITION	18.00	0.53	2.23	0.00	0.01	0.01	581.00
GOBP_MEIOTIC_CELL_CYCLE_PROCESS	15.00	0.56	2.24	0.00	0.01	0.01	477.00
GOBP_NUCLEAR_CHROMOSOME_SEGREGATION	26.00	0.47	2.24	0.00	0.00	0.00	365.00

GOBP_DNA_REPLICATION	18.00	0.54	2.25	0.00	0.01	0.01	317.00
GOBP_ORGANELLE_FISSION	50.00	0.38	2.28	0.00	0.00	0.00	367.00
GOBP_MEIOSIS_I_CELL_CYCLE_PROCESS	11.00	0.65	2.30	0.00	0.00	0.00	477.00
GOBP_MITOTIC_SISTER_CHROMATID_SEGREGATION	20.00	0.53	2.32	0.00	0.00	0.00	266.00
GOBP_RIBONUCLEOPROTEIN_COMPLEX_BIOGENESIS	23.00	0.51	2.36	0.00	0.00	0.00	672.00
GOBP_MEIOTIC_CELL_CYCLE	17.00	0.58	2.39	0.00	0.00	0.00	477.00
GOBP_CHROMOSOME_SEGREGATION	31.00	0.53	2.69	0.00	0.00	0.00	365.00
