# Supplementary figures and images for: A Novel Pseudogene Methylation Signature to Predict Temozolomide Outcome in Non-G-CIMP Glioblastomas
Source: J Oncol. 2022 Jun 6;2022:6345160. doi: 10.1155/2022/6345160 (PMC9194959; doi:10.1155/2022/6345160)

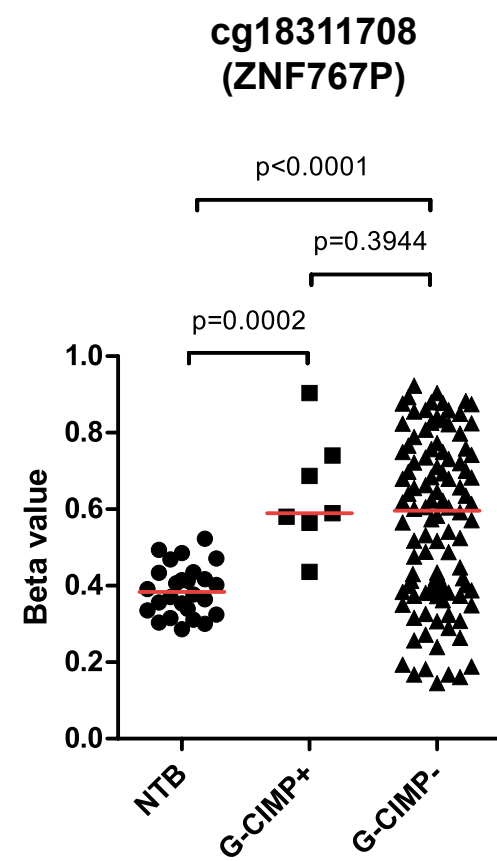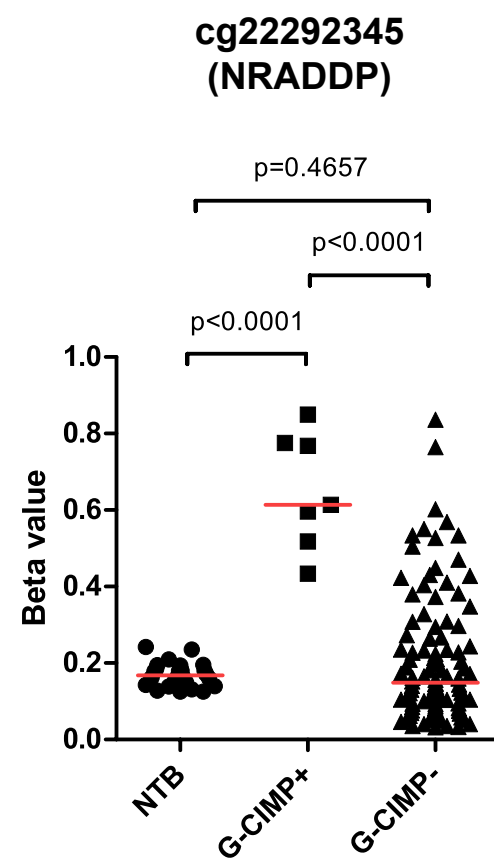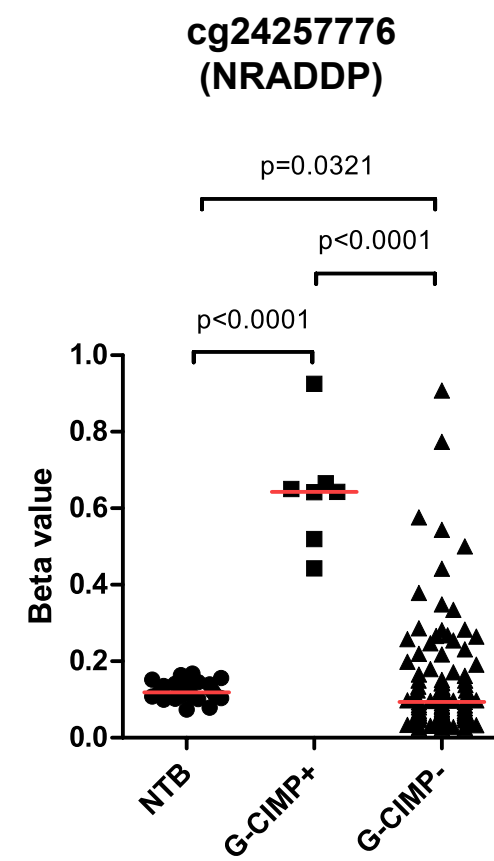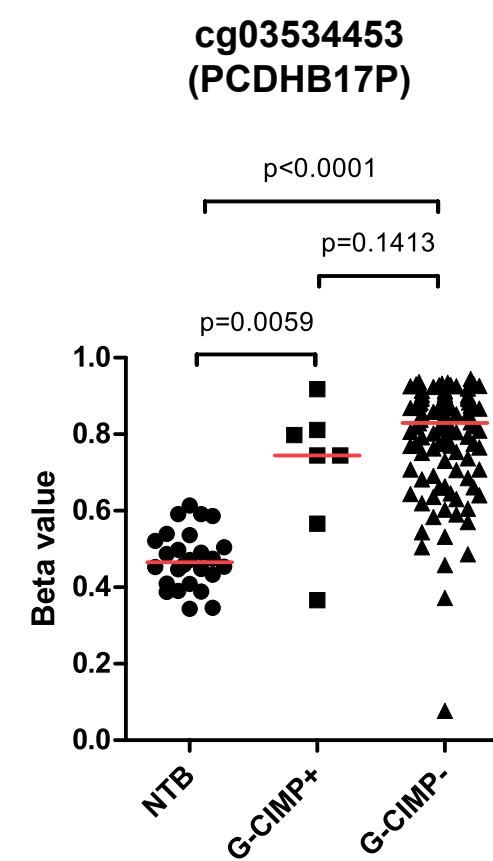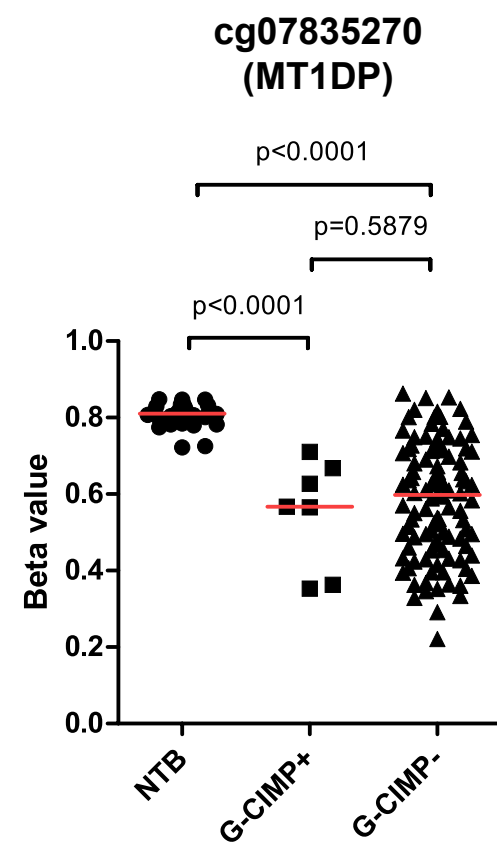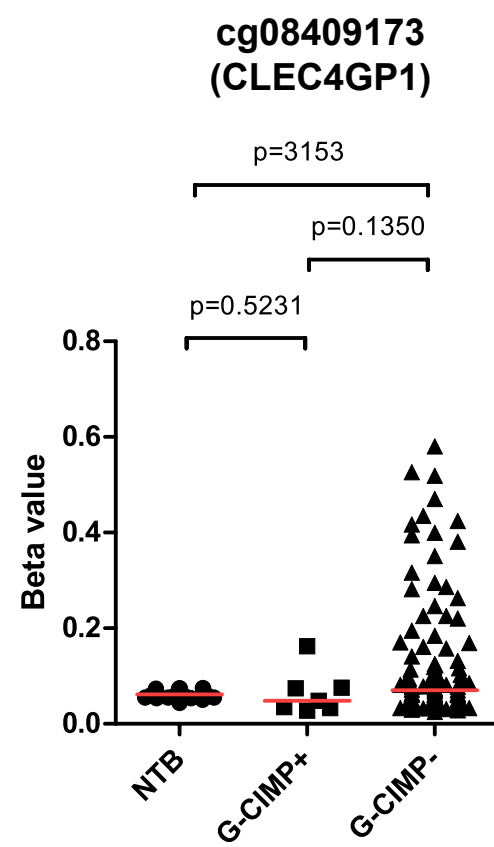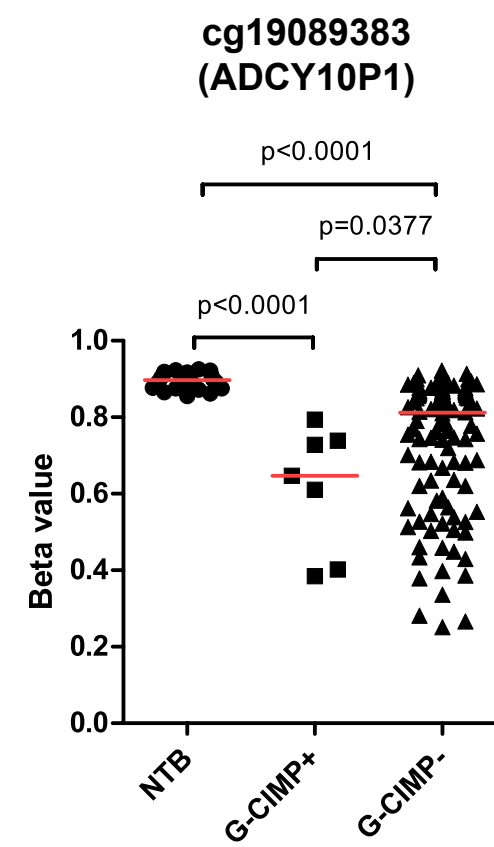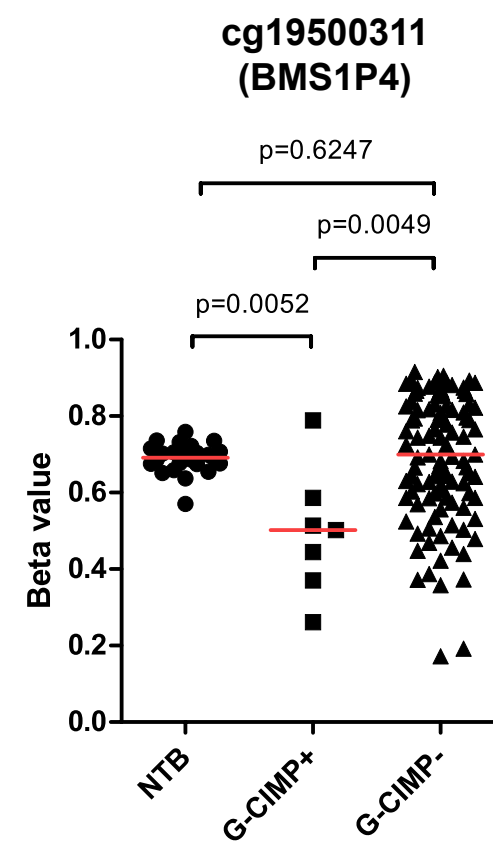

Supplement: Supplementary Materials — Figure S1: Comparison of the methylation states of the 8 CpGs between GBMs of each G-CIMP phenotype and NTBs; NTBs were obtained from GSE63347 and GBMs of each G-CIMP phenotype from TCGA; NTB = nontumor brains; GBM = glioblastoma; and G-CIMP = glioma CpGs island methylator phenotype. Figure S2: Comparison of the expression states of the 5 available pseudogenes between GBMs of each G-CIMP phenotype and NTBs from CGGA; GBM = glioblastoma; G-CIMP = glioma CpGs island methylator phenotype; and CGGA = China Glioma Genome Atlas. Figure S3: Transcriptional levels of CLEC4GP1 and ZNF767P in common GBM cell lines. Table S1: Univariate and multivariate Cox regression analyses in low-risk group of non-G-CIMP GBMs with RT/TMZ or RT alone. Table S2: GSEA analysis of low-risk and high-risk non-CIMP GBMs from TCGA. [file 6345160.f5.zip › 6345160.f2.pdf]

**cg18311708**  
**(ZNF767P)**

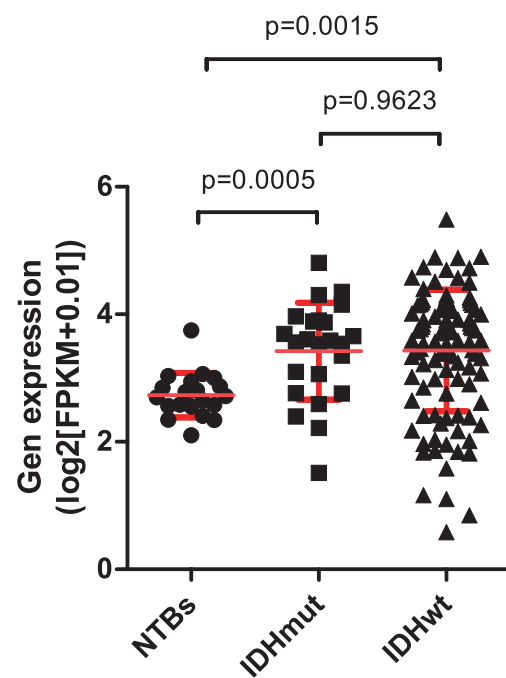

**cg03534453**  
**(PCDHB17P)**

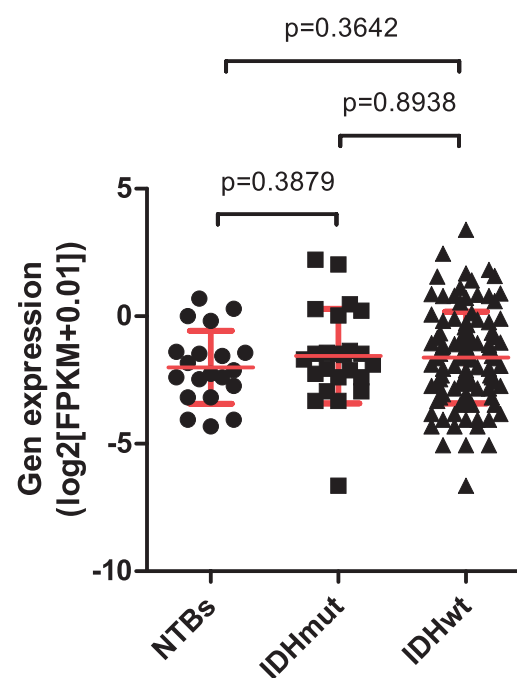

**cg08409173**  
**(CLEC4GP1)**

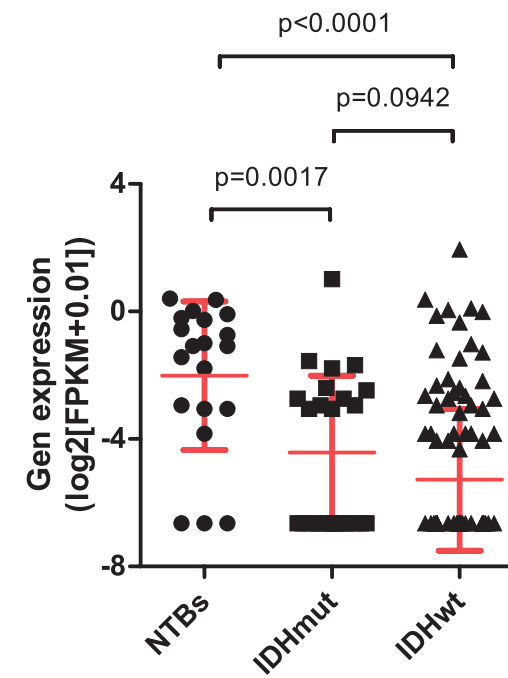

**cg19089383**  
**(ADCY10P1)**

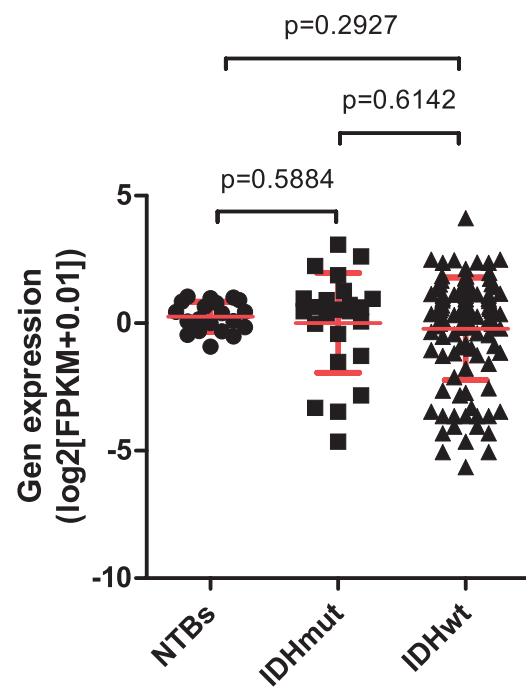

**cg195000311**  
**(BMS1P4)**

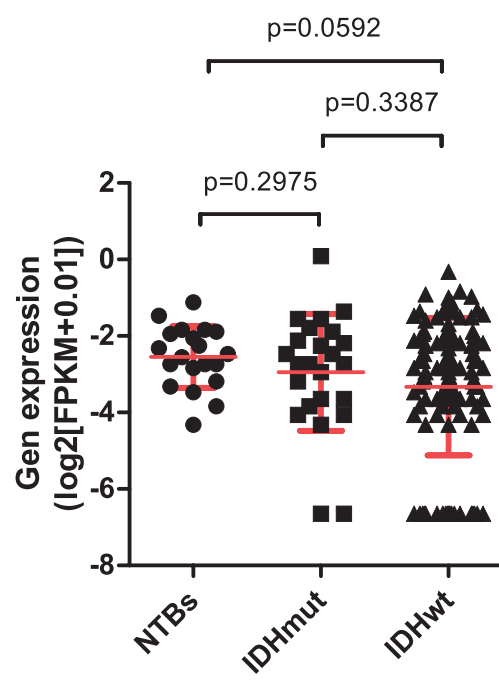

Supplement: Supplementary Materials — Figure S1: Comparison of the methylation states of the 8 CpGs between GBMs of each G-CIMP phenotype and NTBs; NTBs were obtained from GSE63347 and GBMs of each G-CIMP phenotype from TCGA; NTB = nontumor brains; GBM = glioblastoma; and G-CIMP = glioma CpGs island methylator phenotype. Figure S2: Comparison of the expression states of the 5 available pseudogenes between GBMs of each G-CIMP phenotype and NTBs from CGGA; GBM = glioblastoma; G-CIMP = glioma CpGs island methylator phenotype; and CGGA = China Glioma Genome Atlas. Figure S3: Transcriptional levels of CLEC4GP1 and ZNF767P in common GBM cell lines. Table S1: Univariate and multivariate Cox regression analyses in low-risk group of non-G-CIMP GBMs with RT/TMZ or RT alone. Table S2: GSEA analysis of low-risk and high-risk non-CIMP GBMs from TCGA. [file 6345160.f5.zip › 6345160.f3.pdf]

## ZNF767P

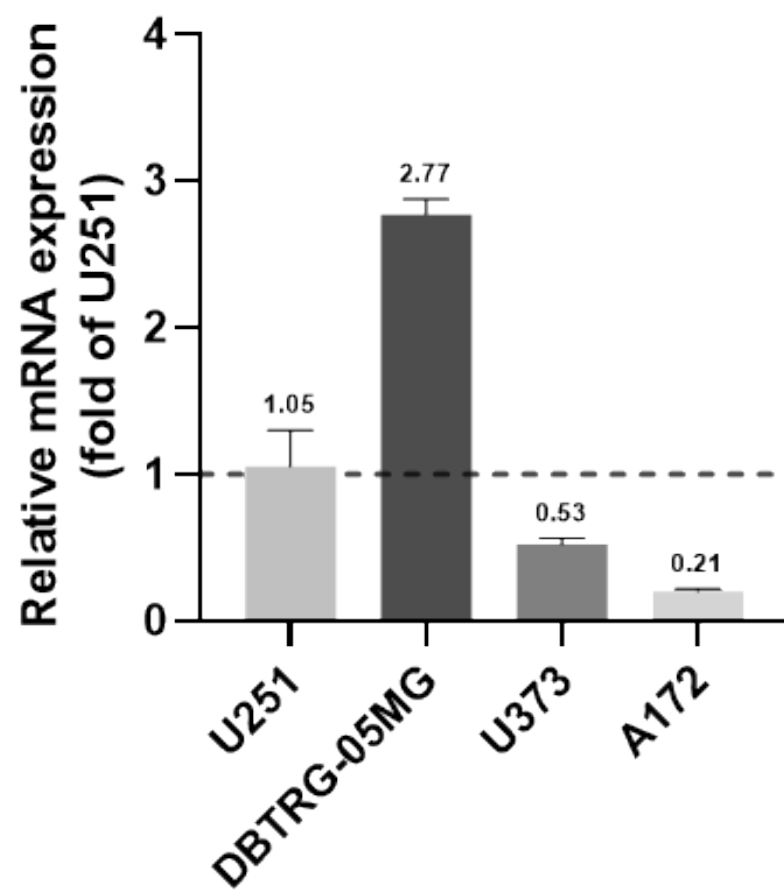

## CLEC4GP1

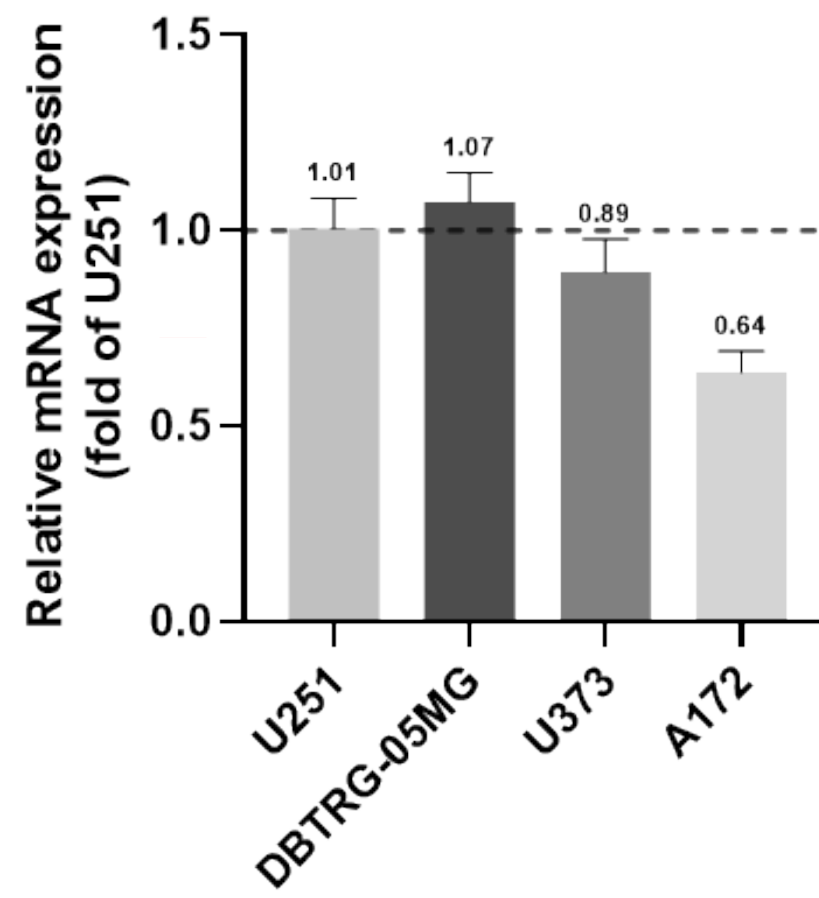

Supplement: Supplementary Materials — Figure S1: Comparison of the methylation states of the 8 CpGs between GBMs of each G-CIMP phenotype and NTBs; NTBs were obtained from GSE63347 and GBMs of each G-CIMP phenotype from TCGA; NTB = nontumor brains; GBM = glioblastoma; and G-CIMP = glioma CpGs island methylator phenotype. Figure S2: Comparison of the expression states of the 5 available pseudogenes between GBMs of each G-CIMP phenotype and NTBs from CGGA; GBM = glioblastoma; G-CIMP = glioma CpGs island methylator phenotype; and CGGA = China Glioma Genome Atlas. Figure S3: Transcriptional levels of CLEC4GP1 and ZNF767P in common GBM cell lines. Table S1: Univariate and multivariate Cox regression analyses in low-risk group of non-G-CIMP GBMs with RT/TMZ or RT alone. Table S2: GSEA analysis of low-risk and high-risk non-CIMP GBMs from TCGA. [file 6345160.f5.zip › 6345160.f4.pdf]
